# Complete Mitochondrial DNA Sequences of the Threadfin Cichlid (*Petrochromis trewavasae*) and the Blunthead Cichlid (*Tropheus moorii*) and Patterns of Mitochondrial Genome Evolution in Cichlid Fishes

**DOI:** 10.1371/journal.pone.0067048

**Published:** 2013-06-24

**Authors:** Christoph Fischer, Stephan Koblmüller, Christian Gülly, Christian Schlötterer, Christian Sturmbauer, Gerhard G. Thallinger

**Affiliations:** 1 Institute for Genomics and Bioinformatics, Graz University of Technology, Graz, Austria; 2 Department of Zoology, Karl-Franzens University of Graz, Graz, Austria; 3 Center for Medical Research, Medical University of Graz, Graz, Austria; 4 Institute of Population Genetics, University of Veterinary Medicine Vienna, Vienna, Austria; BiK-F Biodiversity and Climate Research Center, Germany

## Abstract

The cichlid fishes of the East African Great Lakes represent a model especially suited to study adaptive radiation and speciation. With several African cichlid genome projects being in progress, a promising set of closely related genomes is emerging, which is expected to serve as a valuable data base to solve questions on genotype-phenotype relations. The mitochondrial (mt) genomes presented here are the first results of the assembly and annotation process for two closely related but eco-morphologically highly distinct Lake Tanganyika cichlids, *Petrochromis trewavasae* and *Tropheus moorii*. The genomic sequences comprise 16,588 bp (*P. trewavasae*) and 16,590 bp (*T. moorii),* and exhibit the typical mitochondrial structure, with 13 protein-coding genes, 2 rRNA genes, 22 tRNA genes, and a non-coding control region. Analyses confirmed that the two species are very closely related with an overall sequence similarity of 96%. We analyzed the newly generated sequences in the phylogenetic context of 21 published labroid fish mitochondrial genomes. Consistent with other vertebrates, the D-loop region was found to evolve faster than protein-coding genes, which in turn are followed by the rRNAs; the tRNAs vary greatly in the rate of sequence evolution, but on average evolve the slowest. Within the group of coding genes, ND6 evolves most rapidly. Codon usage is similar among examined cichlid tribes and labroid families; although a slight shift in usage patterns down the gene tree could be observed. Despite having a clearly different nucleotide composition, ND6 showed a similar codon usage. C-terminal ends of Cox1 exhibit variations, where the varying number of amino acids is related to the structure of the obtained phylogenetic tree. This variation may be of functional relevance for Cox1 synthesis.

## Background

With at least 2,200 known species, cichlid fishes (Cichlidae), the freshwater-offshoot of the predominantly marine perciform suborder Labroidei, are among the most species-rich families of all teleost fishes. Their center of radiation lies in the Great Lakes of East Africa where an enormous diversity has evolved within the past 10 million years. Hence these lakes provided an environment for a stunningly rapid sequence of speciation events that generated one of today’s most diverse endemic species assemblages, in terms of morphology, behavior and ecology [1], [2]. With an estimated age of 9–12 million years, Lake Tanganyika is the oldest of the East African Great Lakes [3] and harbors a cichlid species flock that has reached a mature state of radiation in which species exhibit highly distinct eco-morphological characteristics [2].

The study species *Petrochromis trewavasae* (PT) and *Tropheus moorii* (TM) belong to the tribe Tropheini, a monophyletic group of littoral cichlid fishes endemic to Lake Tanganyika [4], [5]. They have been chosen for comparative genome analysis because of their markedly different trophic morphology despite an expected very close genetic relatedness (they diversified within 2–3 million years from a common generalized ancestor [5]). The results presented in this report are the first outcome of an ongoing whole-genome assembly, annotation and comparative analyses project for these two species.

The typical mitochondrial genome exclusively encodes a set of 13 core proteins, which are essential parts of the electron transport chain and oxidative phosphorylation units, and all RNA genes necessary for their translation within the mitochondrion. Mitochondrial genes show an elevated rate of mutation as compared with nuclear genes; this is probably because mtDNA is surrounded by free radicals and reactive oxygen species generated by the respiratory chain operating in the very close neighborhood, and the activity of a less sophisticated DNA repair system, as compared with the nuclear equipment [6], [7]. Although the vital necessity of mitochondrial genes puts strong purifying selection on these sequences, the high mutation rate causes not only somatic mutations accumulating with age, but also germline mutations giving rise to regional variation within and divergence between species [8]. Over the last decades, the characteristics of mitochondrial DNA (mtDNA), in particular the high mutation rate and the near-absence of genetic recombination, have made it the most widely used marker for estimating genetic diversity among species [9]. There is a wealth of literature on mtDNA evolution covering diverse species (reviewed in [9]); however, to our knowledge this is the first study, which compares the available complete mt genomes of cichlids, beyond the construction of phylogenetic trees, in the context of their related families. *P. trewavasae* and *T. moorii* are placed in a phylogeny which is subsequently used as reference for comparative analyses on sequence patterns and codon usage. Aside from comparative analyses, relative substitution rate analysis has been performed for all mt genes or regions in order to get a more comprehensive view of the dynamics of sequence divergence. In the course of comparative analyses, an unexpected major variation in one of the subunits of the cytochrome c oxidase (CcO) has been discovered.

## Results

### Assembly and Genome Organization

In the course of whole-genome sequencing the mitochondria (PT/TM) were recorded with ∼3,600-fold/∼2,500-fold coverage on average (where the lowest per base coverage did not fall below 1,600-fold/860-fold); the respective reads could be assembled into single contigs for both species. Analysis of the integrity and of distances of paired-end reads rules out misassemblies. Variant detection (CLC Workbench: Quality-based Variant Detection; 3% variant frequency threshold) also rules out ambiguous base calls in the consensus sequences; with a 3% threshold heteroplasmy (the presence of more than one mtDNA variant in an individual) was not detectable in the TM genome and only one single site in the PT genome exhibited an insertion/deletion (InDel) variant with a frequency of 6% (position 10,773 in the mtDNA or position 384 within ND4; codon change: **ATT** → **AT-**). The induced frame shift causes a stop codon at 10,783.10,785 leading to a truncated 132 amino acid (aa) protein as compared with the 460 aa ND4.

**Figure 1:**
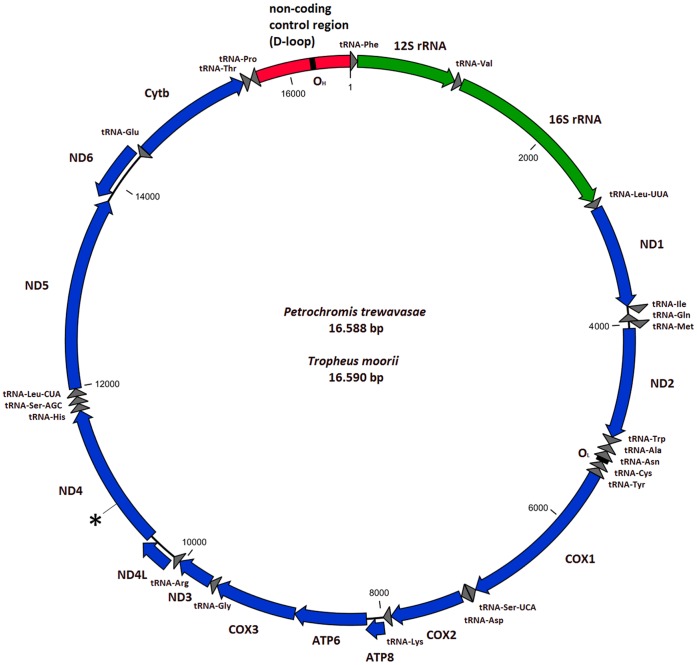
Structure of the mitochondrial genomes of*P. trewavasae* and *T. moorii*. As the differences in genomic structures of both species are too minor to be resolved in this view, only one representative figure is shown. Direction of arrows denotes the strand on which a particular feature resides (H-strand clockwise); staggered arrows indicate an overlap of neighbouring features (for details see **Table 1**). The size of the genome is 16,588 bp for *P. trewavasae* and 16,590 bp for *T. moorii*, where both contain the known 13 protein-coding genes of the respiratory chain (ND1, ND2, COX1, COX2, ATP8, ATP6, COX3, ND3, ND4L, ND4, ND5, ND6 and CYTB), 22 interspersed transfer RNA genes, 2 ribosomal RNA genes (12S and 16S rRNA) and the non-coding control region (D-loop region). The marked position indicates the mutation in the ND4 gene of *P. trewavasae* (causing a frame shift), and the location of the almost immediately following induced stop codon.

As expected, the structure of the mitochondrial genome of each of the newly sequenced species is similar to those of other cichlids characterized so far (**Table 1** **,** **Figure 1**); the same types, number and order of genomic features are present. The size of the genome is 16,588 bp for *P. trewavasae* and 16,590 bp for *T. moorii*, where both contain the known 13 protein-coding genes (in order of occurrence: ND1, ND2, COX1, COX2, ATP8, ATP6, COX3, ND3, ND4L, ND4, ND5, ND6, CYTB), 22 interspersed transfer RNA genes, 2 ribosomal RNA genes (12S and 16S rRNA) and the non-coding control region (CR; also termed displacement loop region or D-loop). The overall base composition of the two H-strand sequences is highly similar with A = 27.5%/27.3%, T = 26.5%/26.4%, G = 15.7%/15.9% and C = 30.3%/30.4% (PT/TM); the low G and increased A+T (54%/53.7%) contents are consistent with the patterns observed in other vertebrates. Also the overall sequence similarity is high with 96% of nucleotides being identical between *P. trewavasae* and *T. moorii*. Details on feature sequence similarities can be found in the Supplementary Information (**Table S2 in File S1**). Sequence data has been deposited in EMBL under accession numbers [EMBL:HE961974] (PT) and [EMBL:HE961975] (TM).

**Table 1:** Organization of the mitochondrial genome of*P. trewavasae/T. moorii*.

	Position		Size (bp)	Codon		Intergenicnucleotides	Strand
Name	Start	Stop		Start	Stop		
tRNA^Phe^	**1**/1	**69**/69	**69**/69			**0**/0	**H**/H
12S rRNA	**70**/70	**1012**/1012	**943**/943			**0**/0	**H**/H
tRNA^Val^	**1013**/1013	**1084**/1084	**72**/72			**0**/0	**H**/H
16S rRNA	**1085**/1085	**2776**/2777	**1692**/1693			**0**/0	**H**/H
tRNA^Leu^	**2777**/2778	**2850**/2851	**74**/74			**0**/0	**H**/H
ND1	**2851**/2852	**3825**/3826	**975**/975	**ATG**/ATG	**TAG**/TAA	**3**/3	**H**/H
tRNA^Ile^	**3829**/3830	**3898**/3899	**70**/70			**−1**/−1	**H**/H
tRNA^Gln^	**3898**/3899	**3968**/3969	**71**/71			**−1**/−1	**L**/L
tRNA^Met^	**3968**/3969	**4036**/4037	**69**/69			**0**/0	**H**/H
ND2	**4037**/4038	**5082**/5083	**1046**/1046	**ATG**/ATG	**TA+**/TA+	**0**/0	**H**/H
tRNA^Trp^	**5083**/5084	**5154**/5155	**72**/72			**1**/1	**H**/H
tRNA^Ala^	**5156**/5157	**5224**/5225	**69**/69			**1**/1	**L**/L
tRNA^Asn^	**5226**/5227	**5298**/5299	**73**/73			**5**/5	**L**/L
OL	**5304**/5305	**5333**/5334	**30**/30			**0**/0	**L**/L
tRNA^Cys^	**5334**/5335	**5399**/5400	**66**/66			**0**/0	**L**/L
tRNA^Tyr^	**5400**/5401	**5469**/5470	**70**/70			**1**/1	**L**/L
COX1	**5471**/5472	**7066**/7067	**1596**/1596	**GTG**/GTG	**TAA**/TAA	**0**/0	**H**/H
tRNA^Ser^	**7067**/7068	**7137**/7138	**71**/71			**3**/3	**L**/L
tRNA^Asp^	**7141**/7142	**7213**/7214	**73**/73			**5**/5	**H**/H
COX2	**7219**/7220	**7909**/7910	**691**/691	**ATG**/ATG	**T++**/T++	**0**/0	**H**/H
tRNA^Lys^	**7910**/7911	**7983**/7984	**74**/74			**1**/1	**H**/H
ATP8	**7985**/7986	**8152**/8153	**168**/168	**ATG**/ATG	**TAA**/TAA	**−10**/−10	**H**/H
ATP6	**8143**/8144	**8825**/8826	**683**/683	**ATG**/ATG	**TA+**/TA+	0/0	**H**/H
COX3	**8826**/8827	**9609**/9610	**784**/784	**ATG**/ATG	**T++**/T++	**0**/0	**H**/H
tRNA^Gly^	**9610**/9611	**9681**/9682	**72**/72			**0**/0	**H**/H
ND3	**9682**/9683	**10030**/10031	**349**/349	**ATG**/ATG	**T++**/T++	**0**/0	**H**/H
tRNA^Arg^	**10031**/10032	**10099**/10100	**69**/69			**0**/0	**H**/H
ND4L	**10100**/10101	**10396**/10397	**297**/297	**ATG**/ATG	**TAA**/TAA	**−7**/−7	**H**/H
ND4	**10390**/10391	**11770**/11771	**1381**/1381	**ATG**/ATG	**T++**/T++	**0**/0	**H**/H
tRNA^His^	**11771**/11772	**11839**/11840	**69**/69			**0**/0	**H**/H
tRNA^Ser^	**11840**/11841	**11906**/11907	**67**/67			4/4	**H**/H
tRNA^Leu^	**11911**/11912	**11983**/11984	**73**/73			**0**/0	**H**/H
ND5	**11984**/11985	**13822**/13823	**1839**/1839	**ATG**/ATG	**TAA**/TAA	**−4**/−4	**H**/H
ND6	**13819**/13820	**14340**/14341	**522**/522	**ATG**/ATG	**TAG**/TAA	**0**/0	**L**/L
tRNA^Glu^	**14341**/14342	**14409**/14410	**69**/69			**4**/4	**L**/L
Cytb	**14414**/14415	**15554**/15555	**1141**/1141	**ATG**/ATG	**T++**/T++	**0**/0	**H**/H
tRNA^Thr^	**15555**/15556	**15626**/15627	**72**/72			**0**/0	**H**/H
tRNA^Pro^	**15627**/15628	**15696**/15697	**70**/70			**0**/0	**L**/L
CR (D-loop)	**15697**/15698	**16588**/16590	**892**/893			**0**/0	**H**/H

For feature identity details see Supplementary **Table S2 in File S1**. Annotations of tRNA anticodons and origins of replication are omitted in this table but are available in the EMBL entries.

#### Protein-coding genes

In both P. trewavasae and T. moorii 13 protein-coding genes in the same size, orientation and relative position can be identified. They are built from 11,473 nucleotides in total and thus make up ∼ 69.16% of the respective genome. The average similarity of coding sequences is 96% at the nucleotide and 99% at the amino acid level. Except for ND6, being encoded on the light strand, all coding genes are located on the heavy strand; ND6 shows the typical shift to G and T in nucleotide content. In both species ATP8 and ATP6 overlap by 10 nucleotides, ND5 and ND6 (opposite strand) by 4, and ND4L and ND4 share 7 nucleotides. Also in both species, all coding genes use **ATG** as start codon with the exception of COX1 initiating with **GTG.** In P. trewavasae the stop codons are: **TAA** as translation terminator for COX1, ATP8, ND4L and ND5; **TAG** is used in ND1 and ND6; the truncated codons **TA+** and **T++** appear in ND2 and ATP6, and in COX2, COX3, ND3, ND4 and CYTB, respectively. The situation in T. moorii is quite similar but not identical, as ND1 and ND6 use **TAA** instead of **TAG**. Reading frame overlaps and incomplete stop codons have previously been observed in mitochondria [10]–[12].

#### RNA genes

The typical small (12S rRNA) and large (16S rRNA) ribosomal subunits were identified, where the 12S rRNA has a length of 943/943 bp (99% id.) and the 16S measures 1,692/1,693 bp (97% id.) (PT/TM). As with some coding genes, 3 of the 22 tRNA genes also show overlaps; in both species tRNA-Gln-CAA formally shares one nucleotide at either end, upstream with tRNA-Ile-AUC and downstream with tRNA-Met-AUG. Overall sequence similarity of tRNA genes is 99%.

#### Control region

The length of the non-coding D-loop region is 892 bp in P. trewavasae and 893 bp in T. moorii, with a sequence similarity of 93%. Dividing these regions based on variability [10], [13] yields: i) a hypervariable domain containing a termination-associated sequence (TAS) [14], ii) a central conserved domain containing the conserved sequence blocks CSB-E and CSB-F and the origin of heavy strand replication (O_H_), and iii) a variable domain comprising the three conserved blocks CSB1, CSB2 and CSB3 (**Table 2**).

**Table 2:** Variable regions and conserved blocks in the non-coding D-loop region.

Species	Item	Sequence	Start	End
PT/TM	**Domain 1**	–	1/1	279/280
PT	**TAS**	ACGCAATGCATATATGTATTAACACCATTGTTTTATATTAAACAT	23/23	67/67
TM	**TAS**	ACGCAATGCATATATGTATTA**T**CACCATT**A**TTTTATAT**C**AAACAT		
PT/TM	**Domain 2**	–	280/281	637/637
PT	**CSB-F**	ATGTAGTAAGAGCCCACC	280/281	297/298
TM	**CSB-F**	ATGTAGTAAGAGCCCACC		
PT	**CSB-E**	AGCGTGTGGGGGGT	502/503	515/516
TM	**CSB-E**	AG**A**GTGTGG**C**GGGT		
PT	**OH**	CTTTTTTTTTTTCCTTTCACTTGACATCTCAGAGTG	519/520	554/555
TM	**OH**	**T** TTTTTTTTTTTCCTTTCA**T**TTGACATC**C**CAGAGTG		
PT/TM	**Domain 3**	–	638/638	892/893
PT	**CSB1**	ATTGCATAACTGATATCATGAGCATA	638/638	663/663
TM	**CSB1**	ATTGCATAACTGATATCATGAGCATA		
PT	**CSB2**	AAACCCCCCCTACCCCC	728/728	744/744
TM	**CSB2**	AAACCCCCCCTACCCCC		
PT	**CSB3**	TGTAAACCCCCCGGAAACAG	773/773	792/792
TM	**CSB3**	TG**C**AAACCCCCCGGAAACAG		

### Phylogenetic Relationships

**Figure 2:**
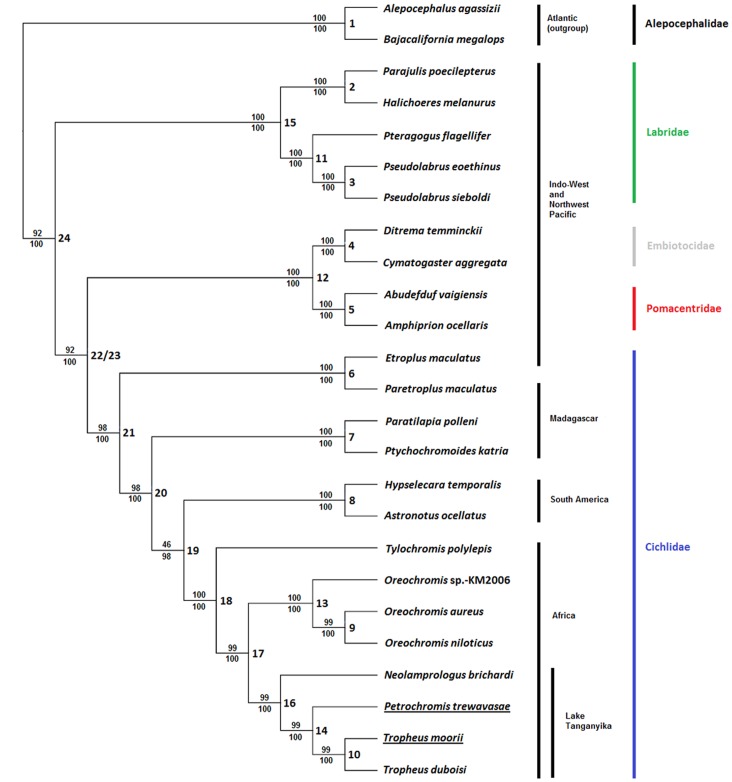
Phylogenetic relationships among labroid families analyzed in this study. Shown is a representative cladogram based on all sequences except those of tRNAs (i.e., data set #3), with numbers representing RAxML bootstrap values above and Bayesian posterior probabilities below the branches. Numbers at internal nodes relate to entries in the Supplementary **Table S6 in File S1**, where all calculated support values are given. The tree was rooted with the sequences of two outgroup species: *Bajacalifornia megalops* and *Alepocephalus agassizii*.

Trees generated based on the sequences of coding and rRNA genes (i.e., data set #2) were topologically identical to those additionally based on D-loop regions (i.e., data set #3). However, topologies of trees based on coding sequences only (i.e., data set #1) or amino acid sequences differed in some aspects (see Supplementary **Figure S2 in File S1**). As expected, *P. trewavasae* and *T. moorii* were joined with *T. duboisi* to form the tribe Tropheini. The remaining branching pattern resembles previous results [10], [15], [16]; the four examined families are monophyletic, where Labridae represent the most distantly related. A representative cladogram constructed from all sequence information except that of tRNAs (i.e., data set #3), and a representative phylogram with branch lengths as determined by maximum likelihood (ML) on a gene-wise partitioned data set (#3) are shown in **Figure 2** and **Figure S3 in File S1**, respectively. An overview of obtained support values is given in the Supplementary Information (**Table S6 in File S1**).

### Relative Rate of Gene Evolution

**Figure 3:**
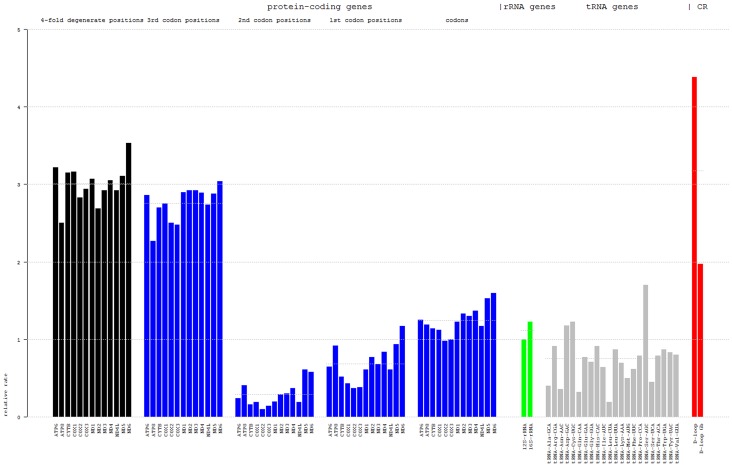
Relative rate of molecular evolution. Bars represent the coefficients of linear least squares regression, where regressions of the pairwise distances of all genes were calculated against the distances of the 12S rRNAs (for an example see Supplementary **Figure S1 in File S1**). Horizontal lines indicate group mean values (from left to right: **3.01, 2.76, 0.29, 0.68, 1.25, 1.12, 0.75**). D-loop (**4.38**) represents the full non-coding region, whereas D-loop Gb (**1.97**) is based on a Gblocks-filtered multiple sequence alignment (InDels removed). For individual bar heights see **Table S4A in File S1**.

When insertions and deletions were considered, in the pairwise distance and regression-based approach (DR), the highest relative rates were observed for the D-loop regions (DR: 4.38 over the reference 12S rRNA) (**Figure 3**); whereas in the Bayesian mean rates approach (BMR) (**Figure S5 in File S1**) D-loops fell back (BMR: 3.21). D-loop regions contain several conserved sequence blocks and clover-leaf (tRNA-like) structures presumably functional in the regulation of transcription and replication [17], [18], but the remaining regions are variable to highly variable. The respective sequence alignment also exhibits major insertions and deletions, which were entirely removed with Gblocks (D-loop Gb; CR: 1.97; BMR: 2.42) in order to provide a comparative view on the effect of large scale variation.

tRNAs vary greatly in their substitution rates (DR: 0.19–1.7, mean 0.75; BMR: 0.20–1.69, mean 0.84), from being nearly stable to exhibiting rates above those of the rRNAs; but on average they have low rates comparable to 1^st^ codon positions. Interestingly, in cases where two tRNAs anticode for the same amino acid, the relative rates within these pairs are markedly different (DR: tRNA-Ser: UCA 0.45/AGC 1.7 and tRNA-Leu: CUA 0.19/UUA 0.87; BMR: 0.39/1.28 and 0.20/0.76). The 16S rRNA (DR: 1.23; BMR: 1.27) as well as the protein-coding genes (DR: 0.98–1.6, 1.25; BMR: 2.17–5.23, 3.14) were found to evolve faster than the 12S rRNA. Using the BMR approach, coding genes were comparable to or even faster than the D-loop (due to 3^rd^ codon positions). The neutral theory of molecular evolution [19] predicts that synonymous sites in protein-coding genes will evolve faster than non-synonymous sites due to the difference in selection pressure. The position dependent rate differences shown in **Figure 3** (and **Figure S5 in File S1** as well) are consistent with this theory and with examples in literature; any mutation at the second position (DR: 0.10–0.61, 0.29; BMR: 0.06–0.72, 0.28) is non-synonymous and should be under strong purifying selection, whereas many mutations at the third codon position (DR: 2.27–3.04, 2.76; BMR: 6.95–26.72, 11.49) and at least some at the first position (DR: 0.37–1.17, 0.68; BMR: 0.31–1.94, 0.95) are synonymous and should therefore be more likely to be fixed in a population. Taking substitution rates of 4-fold degenerate codon positions (DR: 2.5–3.53, 3.01; these rates could not reliably be estimated with the model-based approach) as approximation of the mutation rate (although there are indications that synonymous sites in vertebrate genomes are also subject to selection [20]), indicates that purifying selection is acting on all coding and RNA genes to some extent (**Figure 3**). This is expected by the vital necessity of mitochondrial genes, and is for coding genes also backed by codon selection analysis results (**Table S9 in File S1**).

### Codon Usage

**Figure 4:**
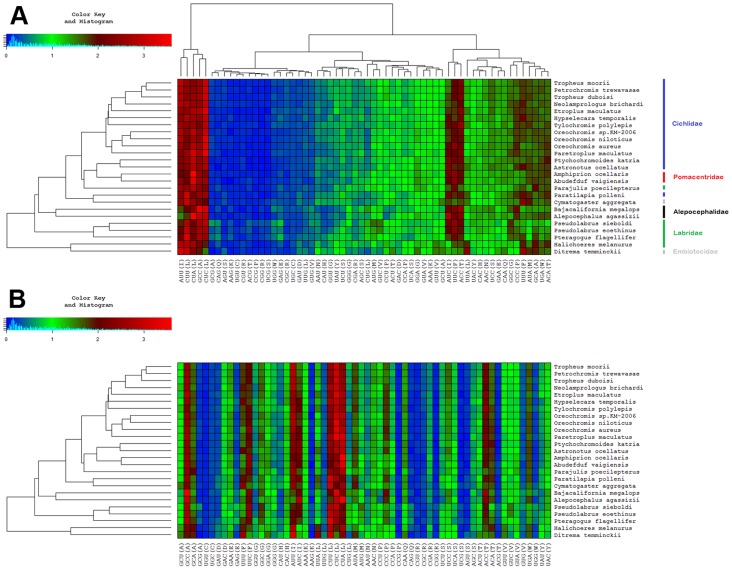
Codon usage. Shown is the codon distribution of all merged protein-coding genes for all considered species (for gene-wise analysis results see Supplementary **Figure S6 in File S1**). Color key: The green region around one implies that a codon was observed about 1 time in 60 codons, higher red and lower blue values indicate deviations of uniform distribution (as factors); thereby also amino acid distribution is implicitly visible. **A** Hierarchical clustering (Lance-Williams [85]; average linkage method) of codon patterns (y-axis) approximately groups together the examined families. Closely related species (i.e. from the same tribe) generally have highly similar codon usage. Clustering on codon frequencies (x-axis) facilitates the visual identification of deviations. **B** Synonymous codons are listed sequentially to allow for quick evaluation of relative synonymous codon usage. For instance, the first 4 columns depict the codon usage for Alanine, where obviously the codon GCG is hardly used and codons GCU, GCC and GCA are used by all species with a preference for GCC.

Our analysis shows that also in the present data set the codon usage does vary to some degree between any two species, even the closest related. Furthermore, each pair of genes from within the same genome exhibits shifts in codon preferences (for gene-wise analysis results see **Figure S6 in File S1**). However, in general (i.e. looking at all coding genes concatenated) codon bias in *P. trewavasae* and *T. moorii* is very similar to that observed in other cichlids, and indeed, there is also not much of a difference as compared to the other families considered (**Figure 4**); the same holds true for amino acid composition, which is implicitly shown. There are only minor deviations among cichlid tribes and also among labroid families; though, a shift in usage patterns down the gene tree could be observed. In concordance with the results obtained from phylogenetic analysis, codon usage patterns of species belonging to the families Labridae, Embiotocidae or Alepocephalidae (outgroup) exhibit stronger deviations as compared with the families Cichlidae or Pomacentridae. Without the averaging effect of pooling, variations are, as expected, more pronounced in some gene-wise comparisons. Interestingly, although the nucleotide distribution of ND6 is clearly different from the distributions of the remaining genes with a shift towards T and G (ND6∶37.4% T, 12.5% C, 14.9% A, 35.2% G; other genes: 24.8–31.4% T, 28.0–35.7% C, 21.2–30.4% A, 11.2–18.0% G; values are averages over all species), there is no marked deviation in its codon usage (**Figure S6 in File S1**) as compared with the merged genes shown in **Figure 4**.

### C-terminal end Variation in Cox1

**Figure 5:**
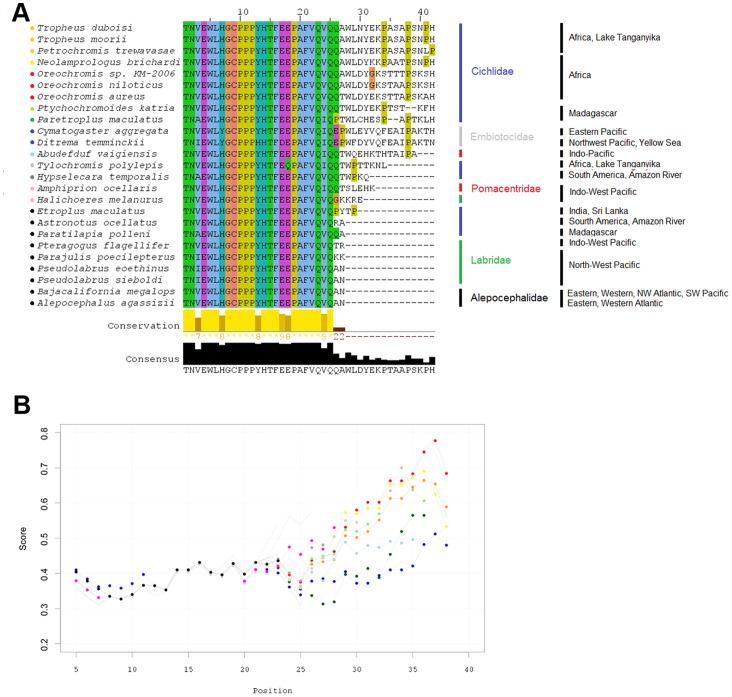
C-terminal end variation in Cox1. **A** C-terminal amino acid sequences of Cox1 exhibit noticeable variations compared to the rather minor differences in all other mitochondrial genes examined; different variant groups can be identified. **B** Amino acid accessibility scale profiles indicate the potential for functional interaction sites in this region (for comparison see examples for full-length Cox1 amino acid accessibility in Supplementary **Figure S4 in File S1**). Position values are related to residue positions in the alignment, scores are normalized (0,1) with a higher score indicating higher accessibility.

For the species studied we have found a variation in the 3′ end of the *COX1* gene. The C-terminal region of its protein Cox1 is hydrophilic and presumably exposed on the matrix side of the inner membrane – and at least in yeast it contains residues interacting with other proteins [21]. The sequences shown in **Figure 5A** are ordered according to a BLOSUM62-based average distance tree; there is a clear relationship with the obtained phylogenetic gene tree (**Figure 2**) and hence with tribe and family relations. Amino acid accessibility scale profiles indicate the potential for functional interaction sites in this terminal region (**Figure 5B**). Cichlid tribes endemic to Africa apparently have gained some residues at the Cox1 C-terminus (*Tylochromis polylepis* to a lesser extent), where 2 variant blocks are obvious (#1: *Tropheus moorii*, *Tropheus duboisi*, *Petrochromis trewavasae*| *Neolamprologus brichardi* a little more distantly related; #2: *Oreochromis* sp. KM-2006, *Oreochromis niloticus* | *Oreochromis aureus* a little more distant). *Ptychochromoides katria* and *Paretroplus maculatus* (both cichlids endemic to Madagascar) show relatedness to these groups, but exhibit two gaps in the alignment each. The Embiotocidae species *Cymatogaster aggregata* and *Ditrema temminckii* (Yellow Sea/East Pacific) show a third variant of similar length but quite different sequence. In the remaining species or tribes (including cichlids in South America, India, and Madagascar) the C-terminus is shorter by up to 15 amino acids.

## Discussion

In line with expectations, the genome structures of *P. trewavasae* and *T. moorii* have been found to be similar to those of other fish species [10]–[12], and vertebrates in general [18]. The genomes comprise the 13 genes coding for proteins of the respiratory chain and the 2 rRNAs and 22 tRNAs necessary for their translation within the mitochondrial matrix; typical properties of mtDNA such as overlaps of adjacent genes and the bias in base compositions between strands are apparent. The nucleotide sequences of these two species turned out to be very similar (see **Table S2 in File S1**), and, under the mutation-friendly conditions in the mitochondrial matrix, the D-loop sequence identity of 93% in particular is rather high and underpins the close relationship. The D-loop region carries most of the regulatory elements, which of course may show effects when mutated, as shown for instance by Suissa *et al*. [22] for humans. Still, this non-coding region is generally expected to be subject to the least selective pressure; the results on relative rates of sequence evolution clearly conform to this expectation when insertions and deletions (DR) are considered. Also in the model-based approach (BMR) the D-loop changes faster than all other regions except for 3^rd^ codon positions (and consequently some coding genes). Generally, genes with different functions, or parts of a gene with different functions, have different functional (and structural) constraints and hence appear to evolve at different rates [23]; moreover, there may be systemic determinants of gene evolution such as dispensability or expression level [24]. The latter point is irrelevant in this case as all mitochondrial genes can be regarded as essential and are known to be transcribed in a polycistronic fashion with a 1∶1 stoichiometry for all genes except rRNAs, whose amount can be separately modulated by a transcription termination point immediately after the two rRNAs [25], [26]. Consistent with results from other vertebrate species in literature [27]–[29], the analyzed fish genes have evolved at quite different rates with the order D-loop≥ CDS>rRNA>tRNA (for averages per group) (**Figures 3** **and S5**). Most genes do not deviate much from a clock-like model of evolution, only ATP8, ND5 and several tRNAs show an increased rate heterogeneity among lineages (for details see **Table S8 in File S1**). ND6 exhibits higher substitution rates than all other protein-coding genes; along with ATP8 and ND5 it is also clearly more susceptible to changes in amino acids (i.e., substitutions in 1^st^ and 2^nd^ codon positions).

The ML approach (PhyML) used by jModelTest apparently caused an overestimation of relative substitution rates (mostly related to low degrees or even lack of reference G-T substitutions [30]) in some cases (**Table S7 in File S1**), making it necessary to dismiss the models chosen by information criteria in favor of manually selected models. This clearly indicates a lack of information in the data which renders some of the results generated by the more sophisticated Bayesian method ambiguous. To be clear, this only concerns one codon partition (4-fold degenerate) and some tRNA sequence alignments, but not the remaining partitions, full coding and rRNA genes or the D-loop. Aside from this, the handling of gap positions in common model implementations does not allow the correct consideration of InDels (i.e. columns in the alignment mostly filled with gaps) present in the control region [31]; hence, there is implausibly little difference between filtered and non-filtered D-loop alignments. These circumstances made us make use of raw distance values with incorporation of fully weighted gaps in distance calculation and a regression approach in order to complement the model-based results (which do have value for assessment of the relative rates among coding and RNA genes).

Phylogenetic analysis was primarily conducted to place the new mitochondrial genomes in an established species tree. We used the complete cichlid mt genomes available from GenBank and complemented them with sequences from species used in previous studies, wherein Mabuchi *et al.* [15] and He *et al.* [10] have shown that each of the families Cichlidae, Pomacentridae, Embiotocidae and Labridae is monophyletic. Our analysis corroborates these monophylies and also the finding that Labridae are genetically more distantly related to the other three families. Likewise, the splitting of species into clades according to their geographic distribution is consistent with previous results (**Figure 2**). We found that the earlier reported, clear separation of Pomacentridae and Embiotocidae was only apparent when coding sequences alone were used in analyses (still with weak support values), otherwise this bifurcation collapsed and the two families formed a clade. However, for the most part the published tree topologies could be verified and *Petrochromis trewavasae* and *Tropheus moorii* fit in the gene tree at the expected positions in the Lake Tanganyika cichlid tribe Tropheini [32].

Generally, the frequencies of alternative synonymous codons have been found to vary, both among species and among genes from the same genome [33], [34]; this is referred to as codon bias or codon usage. Codon bias may exist because of non-randomness in the mutational patterns or due to its contributions to the efficiency and/or the accuracy of protein expression, in which case it would be generated and maintained by selection [33]. In the present data, codon usage also varies between species and also between genes within the same genome; however, looking at all protein-coding genes concatenated, usage patterns are not dramatically different between species (**Figure 4**). A somewhat unexpected finding is that codon usage of ND6 (L strand) is quite similar to the general usage pattern (H strand except ND6). Hierarchical clustering yielded slightly different results depending on the clustering method used. Indeed, the result with the best conformity of the hierarchical tree built on codon usage of all concatenated genes (**Figure 4**) and the ML or BI-based phylogenetic gene tree (**Figure 2**) was achieved using the median linkage algorithm; whereby the families were resolved more accurately, and the outgroup was placed most distantly. However, as it is difficult to interpret the emerging inversions [35] in the dendrograms (which were especially prominent in the gene-wise analyses) results based on the average linkage method are shown. The presence of similar usage patterns (within tribes and at least in most cases, also within families, **Figure 4** and **Figure S6 in File S1**) and the grouping in the respective hierarchical trees, which relates to the obtained gene tree (**Figure 2**), suggests an evolutionary basis of observable differences which is likely to be superimposed by stochastic substitution processes; selection analyses of codon alignments support this assumption, with most codons being under negative selection, but also a considerable number evolving neutrally (**Table S9 in File S1**). Across all coding genes, only one single codon was found to be under positive selection (codon 407 of *COX1*).

The *COX1* gene was also remarkable with respect to the variation in its 3′ end. Its protein Cox1 has been recognized as the foundation upon which further assembly of the cytochrome c oxidase (CcO) occurs; it is the largest subunit of the CcO, and contains heme centers for oxygen reduction [36]. Eukaryotic CcO catalyzes the last step of the mitochondrial respiratory chain, the transfer of electrons from *cytochrome c* to molecular oxygen. This electron transfer is coupled with a proton translocation over the inner mitochondrial membrane; thereby, CcO contributes to the storage of energy in the form of an electrochemical gradient that is used for ATP synthesis. The enzyme is a multimeric complex, in eukaryotes formed by 11–13 protein subunits (11 in yeast, 13 in vertebrates) of nuclear and mitochondrial origin, of which the large, hydrophobic transmembrane subunits Cox1-3 (forming the catalytic core of CcO) are encoded in the mitochondrial genome. CcO plays a central role in respiratory control as its activity is dependent on the intramitochondrial ATP/ADP ratio [36]. The sequence of Cox1 is well conserved across the examined fish species and *Bos. taurus* (and also *H. sapiens*) up to position P508 (relating to position P19 in **Figure 5A**); the remaining 6 (*B. taurus*) or 5 (*H. sapiens*) residues seem to be species specific (data not shown). H503 (H14) is strongly conserved and has been shown to act as a controlling site for dioxygen reduction and proton pumping in *Bos taurus* [37]; as the sequence TFEEP (504–508; or 15–19) is also strongly conserved, it could be relevant for the correct positioning of the histidine residue. Length of the Cox1 amino acid sequences of *B. taurus* and *H. sapiens* is close to that of the shortest versions in the examined fish species.

The observed variation in the C-terminal region might have an effect on the translational regulation of Cox1 synthesis; in yeast mitochondria (*S. cerevisiae*) it has been shown that deletion of the C-terminal 11 or 15 residues of Cox1 by site-directed mutagenesis does not prevent respiratory function but eliminates the assembly-feedback control of Cox1 synthesis. The C-terminal domain of Cox1 is necessary here for assembly-coupled translational down-regulation (for a mechanistic model of the translational regulation of Cox1 see [38]). However, the results obtained in yeast are not directly transferable to fish, especially as the C-terminal end of Cox1 is the least conserved region between yeast and the fish under study (data not shown). The observed high accessibility of amino acids in the C-terminal region does not argue against the assumption of a functional interaction site; a high accessibility of amino acids has been shown to be one of the more reliable predictors for protein interaction sites [39]. However, using sequence information (i.e. primary structure) and amino acid scales in a sliding window approach must be valued as an approximate estimate; moreover, high accessibility of terminal amino acid sequences is common in proteins [40]. Several studies on mammalian tissues suggest that levels of mitochondrial encoded (and not nuclear encoded) subunits are limiting for the assembly of the CcO complex, which is also supported by findings in fish [41]. Acclimation to temperature has an effect on CcO activity in fish tissues (see a review on a number of temperature-related effects on metabolism in [42]). During thermal acclimation to cold, CcO activity may be increased by modulation of the specific activity of the individual enzyme molecule or by a rise in the number of CcO molecules [42]. Concerning the latter, correlations of CcO activity and *COX1* mRNA levels suggest that CcO expression in fish may be regulated at the translational or post-translational level [41]. Considering this together with the findings in yeast of a C-terminal-related translational regulation of Cox1 synthesis, and the fact that Cox1 is the (limiting) basis for the assembly of CcO, it is conceivable that there is a connection between the observed differences in the structure of the C-terminal end of Cox1 and temperature. This is speculation, and indeed, based on information provided in FishBase [43], no clear relationship between natural habitat temperatures and sequence structure could be established for these data (but, there is a tendency). However, due to the central role of Cox1 in energy metabolism it may be worth following this track to investigate possible correlations between the structural differences and other environmental factors.

### Conclusions

The structures of the mitochondrial genomes of *P. trewavasae* and *T. moorii* are similar to those of other vertebrates. Similarly, their patterns of sequence evolution or more specifically the pattern of relative rates of evolution of gene groups is within the range of published results from other species. Despite some rather minor variation, codon usage appears to be highly conserved among all labroid taxa analyzed in this study, which is quite remarkable given the species richness and hence diversification rate of this group of perciform fishes. Particularly high levels of interspecific variation were identified in the 3′ region of the *COX1* gene and, considering sparse evidence from other distantly related organisms, may be of functional relevance for Cox1 synthesis.

## Materials and Methods

### Animal Welfare Statement

Animal treatment reported in this paper complies with the standards of the Animal Welfare Act in Austria and the European Community Directive 86/609. According to the Austrian Animal Experiments Acts (TVG, BGBI.Nr. 501/1989, last changed by BGBI. I Nr. 162/2005), approval was not required because no experimental treatment was performed. Fish were euthanized using an overdose of clove oil and decapitated conforming to the Austrian Animal Welfare legislation.

### Study Species

The sampled specimens of *Tropheus moorii* are F2 offspring of wild caught individuals from the Zambian section of the southwestern shore of Lake Tanganyika near the village Nakaku (08°38′S 30°52′E), which were brought to the University of Graz in 2005. The *Petrochromis trewavasae* specimens used in this study are F1 offspring of wild fish also from the southwestern shore, but further north near the village Katete (08°20′S 30°30′E) and were obtained from an ornamental fish importer. Collection of the parental generation of fish was carried out in the framework of a Memorandum of Understanding between the Department of Fisheries, Ministry of Agriculture and Cooperatives, Zambia, the Department of Biological Sciences at the University of Zambia in Lusaka, the Department of Zoology at the University of Graz, Austria, the Department of Behavioural Ecology at the University of Bern, Switzerland, and the Department of Zoology at the University of Basel, Switzerland, under the research permit issued to CSt by the Zambian Ministry of Home Affairs (permit number: SP006515). Sequence data presented here are based on DNA extractions of a single individual per species; both specimens were about one year old.

### DNA Extraction and Library Preparation

#### DNA extraction

From both species DNA was extracted by using a classic phenol-chloroform procedure [44]. About 40 mg of tissue from skeletal muscles and organs were homogenized using MagnaLyser Beads (Roche Diagnostics; Mannheim, Germany) and digested over night at 50°C with 5 µl of Proteinase K (20 mg/ml; Fermentas; St.Leon-Rot, Germany) in a total volume of 500 µl. For large insert libraries high-molecular weight DNA was extracted by homogenizing, tissue in liquid nitrogen using a pre-chilled mortar and pestle followed by enzymatic digestion (Proteinase K) for 3 hrs and a phenol/chloroform/isomylalcohol (25/24/1) extraction step [44]. Nucleic acids were precipitated from the aqueous phase by adding 1/10 volume of 3 M NaAc pH 5.2 and three volumes of pre-chilled 100% EtOH. After RNA digestion with 10 µl RNase A (10 mg/ml; Fermentas; St.Leon-Rot, Germany) at 37°C for 2 hrs the DNA was precipitated and washed twice with pre-chilled 70% EtOH. DNA was dried at room temperature and resuspended in molecular biology grade water (Roche Diagnostics; Indianapolis, IN, USA). The DNA quantity and quality was evaluated in a 0.8% agarose gel (in 1×TAE) by using a standardized Lambda DNA ladder (Roche Diagnostics; Mannheim, Germany).

#### 454 library preparation and emulsion PCR amplification

Standard shot-gun, 8 kb and 20 kb paired-end libraries were generated for pyrosequencing on a Genome Sequencer FLX (Roche 454 Life Science; Branford, CT, USA) according to the General Library Preparation Method Manual (April 2009) and the Paired End Library Preparation Method Manual –20 kb and 8 kb Span (Oct 2009). The libraries were analyzed and quantitated on a BioAnalyzer 2100 (Agilent Technologies; Waldbronn, Germany) by using a RNA Pico 6000 LabChip. The single stranded libraries were diluted with TE Buffer (10 mM TRIS, 0.1 mM EDTA, pH 8.0) and used to identify the optimal ratio of DNA molecules to capture beads as described in EmPCR Method Manual Lib-L SV (Jan 2010). According to the results from the emulsion titration, an optimized molecule to bead ratio (shotgun libraries: 1.2 and 0.75 molecules per bead; 8-kb paired-end libraries: 0.3 and 0.2 molec. per bead; 20-kb paired-end libraries: 0.2 and 0.4 molec. per bead for the Petrochromis and the Tropheus libraries, respectively) was used to prepare the capture beads following the EmPCR Method Manual Lib-L LV (Jan 2010). The optimal number of DNA molecules was added to the 34×10^6^ capture beads per cup. After adding the amplification mix to the capture beads containing the DNA library, the whole mix was transferred to the oil cup to generate the water in oil emulsion by shaking in a Qiagen TissueLyser II (Qiagen; Hilden, Germany). Subsequent to PCR, emulsions were broken and the beads recovered. The sequencing primer was annealed for final sequencing on the GS FLX instrument.

#### Illumina library preparation

120 bp and 410 bp (true mean insert sizes) paired-end libraries were generated as follows: We fragmented DNA using a Covaris S2 system (Covaris, Inc. Woburn, MA) and purified fragments using the QIAquick PCR purification kit (Qiagen; Hilden, Germany). Paired-end libraries were prepared using the NEBNext DNA Sample Prep modules (New England Biolabs, Ipswich, MA) following the manufacturer’s instructions. Briefly, fragments were end-repaired using Klenow and T4 DNA polymerases and phosphorylated with T4 polynucleotide kinase. Fragments were then 3′-adenylated using Klenow exo-DNA polymerase, and Illumina adapters were added using DNA ligase. Ligation products of ∼400 bp and ∼700 bp were gel-purified using the Qiagen gel extraction kit (Qiagen; Hilden, Germany). To avoid guanine-cytosine (GC) bias introduced during the gel-purification step in the standard Illumina library preparation protocol, the gel slice was dissolved at room temperature instead of heating. The size-selected, adapter-modified DNA fragments were PCR-amplified using PE PCR primers 1.0 and 2.0 (Illumina, San Diego, CA), Phusion DNA polymerase (New England Biolabs, Ipswich, MA) and the following protocol: polymerase activation (98°C for 30 s), followed by 10 cycles (denaturation at 98°C for 10 s, annealing at 65°C for 30 s, and extension at 72°C for 50 s) with a final, 5-min extension at 72°C. Libraries were purified and quantified using the Qubit HS Assay Kit (Invitrogen, Carlsbad, CA, USA).

### Sequencing

#### 454 sequencing

The enriched DNA beads of each individual library were sequenced on a Titanium PicoTiterplate (PTP), where the standard loading procedure was used: 2 million beads per region of a two region PTP device, according to the Sequencing Method Manual (Nov 2010). In the sequencing, run 200 cycles were performed to yield average sequence lengths of 340b for shotgun libraries and 287 to 345b for paired-end libraries. The runs achieved a range from 232 Mb to 459 Mb of sequence information. Data analysis was done using 454 Sequencing System Software 2.6.

#### Illumina sequencing

We performed cluster amplification using the TruSeq PE Cluster Kit v5 on a cluster station, and sequenced each library on two GAIIx lanes using TruSeq SBS 36 Cycle Kits v5 (Illumina, San Diego, CA) with a 2×101 bp paired-end protocol. Sequencing image files were processed using the Sequencing Control Software (SCS) Real Time Analysis (RTA) v2.6 and CASAVA v1.7 to generate base calls and phred-like base quality scores and to remove failed reads. The runs yielded ∼50 Gb of sequence information per species in total.

#### Assembly and annotation

454 and Illumina reads were filtered on quality values (cut-off: base-caller error probability p_error = _0.01; modified-Mott trimming algorithm), number of ambiguous bases per read (max. 2), minimum length (20 bp) and technically introduced sequences (adapters, primers) using the CLC Genomics Workbench 5.5 (CLC Bio; Arhus, Denmark), which was also applied in sequence assemblies besides Newbler 2.6 (454 Life Sciences; Branford, Connecticut, USA). In addition, the Genomics Workbench was used for preparing graphical representations of the annotated mt genomes, for prediction of open reading frames (ORFs) and for general annotation refinement. The ORF search tool was set to find ATG and GTG as start codons; it uses all applicable stop codons by default. According to previous findings [45], non-triplet 3′ ends of protein-coding genes immediately adjacent to the beginning of downstream features and reading T or TA were treated as truncated stop codons. The base annotations were done by BLAST-based [46] homology search. For this, custom R [47] scripts were used to take care of retrieval and parsing of reference mitochondrial sequences obtained from the NCBI nucleotide database (Table S1 in File S1), handling of BLAST searches of extracted reference features against the assembled genomes (using local BLAST+2.2.25 [48]), filtering of the generated results, consensus finding of the most likely feature identities and position coordinates in the genomes examined (based on percent identity, length coverage and a majority rule), and generation of the primary output files containing the annotated sequences in EMBL format, as well as files in FASTA format for subsequent comparative analysis and multiple alignments. As additional support for the reported non-coding RNA locations Infernal 1.0.2 (inference of RNA alignments) [49], LocARNA 1.7.2 [50] and COVE 2.4.4 [51] were used for model-based prediction of rRNA and tRNA sites and anticodons – relying not only on sequence similarities but also on RNA secondary structure. Reported positions of protein-coding genes were reviewed on the basis of predicted ORFs. The origins of replication of the light strand (O_L_) and the heavy strand (O_H_) were defined by multiple alignments with annotated sequences of other species and also by secondary structure search (hairpin; CLC Genomics Workbench). Conserved sequence blocks in the non-coding control region were identified by multiple sequence alignments.

### Phylogenetic Analysis and Multiple Sequence Alignment

The sequences of all genomic features were extracted from the two new sequences and 23 mitogenomes (21 labroid +2 outgroup) retrieved from GenBank (**Table S1 in File S1**) and subjected to phylogenetic analysis. Analyses were conducted with different data sets: #1 contains CDS only, #2 contains CDS and rRNAs, and #3 contains CDS, rRNA and D-loop region sequences. tRNA sequences were omitted as they have been shown to be not particularly suited for phylogenetic inference [52]. Multiple alignments of the individual features (nucleotide and amino acid sequences) were done using first MAFFT [53], [54] (default settings; L-INS-i algorithm for coding sequences and tRNAs, E-INS-i for D-loop regions and rRNAs) and then MUSCLE [55] (default settings) (see details on alignments in **Table S3A** and **S3B in File S1**). Alignments were manually revised using Jalview 2.6.1 [56] and concatenated [57] using R. Some obviously misaligned residues could be traced back to inconsistent annotations of start and stop positions in the NCBI database. The amino acid sequences of coding genes could be used without manual correction to produce nucleotide sequence alignments by back-translation (codon alignments; R), which were used in all analyses. To address the question of possible adverse effects of poorly aligned regions, especially in the D-loop region, data set #3 was additionally filtered with Gblocks 0.91b [58] (stringent standard settings). Phylogenetic trees were generated by applying algorithms based on maximum likelihood (ML) and Bayesian Markov-Chain-Monte-Carlo (MCMC) inference (BI), using the implementations PhyML-aBayes 3.0.1 [59]/RAxML 7.2.8 [60], and MrBayes 3.2 [61] */*MrBayes5D 3.1.2 [62], respectively. All free model parameters were estimated from data. Obtained phylogenetic trees were edited with Mesquite 2.75 [63]. jModelTest 2.0.2 [64] was used for statistical selection of the best-fit model for nucleotide substitution, where Akaike’s information criterion (AIC) [65] was applied as selection strategy [66] (for details see Supplementary Information **Table S5A in File S1**). PhyML was utilized with Shimodaira-Hasegawa approximate likelihood ratio tests (SH-aLRT) for more conservative calculation of branch support values; SH-aLRT is derived from the SH multiple tree comparison procedure [67] and as a nonparametric version of aLRT better suited when model assumptions are severely violated [59]. In any case, the proportion of invariable sites and the gamma shape parameter were estimated from data (8 rate categories) and subtree pruning and regrafting (SPR) was used as the method to estimate tree topologies; a parsimony and 5 random trees were generated for the beginning. The ML approach was also applied in the form of RAxML, employing the GTR+I+G model with 25 rate categories for all gene-wise partitions; 100 rapid bootstrap replicates (standard settings) were used to infer statistical support.

For Bayesian-MCMC analyses two independent runs of four million generations with one cold and three hot chains (T = 0.1) were conducted, sampling the chains every 100^th^ cycle and discarding the burn in samples (6000 trees) in parameter estimation. Analyses were run on gene-wise partitioned data sets, where for each partition the best available substitution model was fit with unlinked model parameters (under linked topology and branch length estimation) with 8 substitution rate categories used to approximate the gamma distribution; MCMC parameters were left at standard settings. In addition, MrBayes was used with the model-jumping approach to integrate out the uncertainty concerning the correct substitution model. Chain stationary and parameter convergence were checked using Tracer [68] and AWTY [69].

The above mentioned tree inference methods were used similarly on multiple alignments of amino acid sequences. Best-fit model selection for amino acid sequence-based analysis was done with ProtTest 3 [70] (for details on AIC-selected models see **Table S5A in File S1**). Due to a broader substitution model support, MrBayes5D was used for analysis of amino acid sequences.

### Relative Rate of Gene Evolution and Molecular Clock Test

To determine the differences in the rate of molecular evolution, p-distances for all genes were calculated against the respective consensus sequences using *cons* and *distmat* from the EMBOSS package [71] – for this analysis the outgroup species *B. megalops* and *A. agassizii* were removed from the data set. Based on the distance values, linear least squares regressions of the pairwise distances of all genes against the respective distances of the 12S rRNAs were calculated and visualized in R (see example in **Figure S1** and **Table S4A in File S1**); for scaling reasons an rRNA gene was used as reference (12S rRNA was arbitrarily chosen). In the present work, the regression coefficients of correlated distance values are referred to as relative rates. This rather unusual approach was taken in order to capture the effect of insertions and deletions, especially in the non-coding region. Rates were also determined in a Bayesian framework (**Table S4B in File S1**); BEAST 1.7.5 [72] was used for Bayesian MCMC-based estimation of mean rates for all genes or partitions, where for each partition the information criterion selected nucleotide substitution model was used (AIC/BIC, jModelTest 2.02; see **Table S7 in File S1**). The tree topology was linked across partitions, whereas parameters of substitution models and clock models were calculated independently. For +G substitution models, 8 gamma categories were used to approximate the gamma distribution. Clock rates were estimated using the lognormal relaxed clock model; clock-like behavior was assessed based on the ucld standard deviation (see Supplementary **Table S8** **in File S1** for details). The Yule process was chosen as tree prior; starting from a random tree, parameters have been calculated with a chain length of 50 MIO (burnin 5 MIO) being sampled every 1000^th^ round. Standard prior distributions were used for all parameters except for the rate priors (ucld.mean and indirectly meanRate) which were set to the gamma distribution (0.001,1000). Parameter distributions were evaluated using Tracer 1.5 [68] and R. Estimated sample sizes (ESS) of reported parameters sampled from the MCMC are all >100 and mostly >200.

### Codon Usage and Selection Analysis

Codon usage and building block distributions were determined for all protein-coding genes, gene-wise and merged, with MEGA 5.05 [73] and R; statistical analyses of distributions and visualization of codon usage in the form of heatmaps was done with R. Selection analysis was conducted using the web service datamonkey [74], which is based on the HyPhy [75] package; codons under positive or negative selection have been identified using several approaches: mixed effects model of evolution (MEME) [76], fixed and random effects likelihood methods (FEL and REL) [77], and the Bayesian MCMC-based fast unbiased approximate Bayesian analysis (FUBAR) [78].

### C-terminal end Variation in Cox1

C-terminal amino acid sequences of Cox1 have been aligned manually according to an average distance tree (BLOSUM62 matrix) using Jalview [56]. Amino acid accessibility was determined with ProtScale [79] using a window size of 9 with fully weighted edges, and amino acid scales (numerical values based on chemical and physical properties assigned to each type of amino acid) as given in [80].

### R

Besides the cited tools, R was generally used for data handling and computational tasks, where the R packages Biostrings [81], seqinr [82], ape [83] and igraph [84] were utilized.

## Supporting Information

File S1 **This file contains Figure S1–S6 and Tables S1–S9.**
(PDF)
