# Supplementary material for: Complete Mitochondrial DNA Sequences of the Threadfin Cichlid (Petrochromis trewavasae) and the Blunthead Cichlid (Tropheus moorii) and Patterns of Mitochondrial Genome Evolution in Cichlid Fishes
Source: PLoS One. 2013 Jun 24;8(6):e67048. doi: 10.1371/journal.pone.0067048 (PMC3691221; doi:10.1371/journal.pone.0067048)
Supplement: File S1 — This file contains Figure S1–S6 and Tables S1–S9. (PDF) [file pone.0067048.s001.pdf]

Supplementary Information

Complete mitochondrial DNA sequences of the threadfin cichlid (*Petrochromis trewavasae*) and the blunthead cichlid (*Tropheus moorii*) and patterns of mitochondrial genome evolution in cichlid fishes

Table S1 **Reference sequences.** The complete mitochondrial genomes listed were downloaded from the NCBI nucleotide database. Besides acting as source of annotated sequence data in the homology-based annotation process these sequences were also used for the construction of phylogenetic trees. Stated geographic ranges were retrieved from FishBase, where also detailed maps of sightings are available in most cases.

Perciformes

Accession number	Name	Classification	Geographic range
[GenBank:NC_009063]	***Tropheus duboisi***	Labroidei Cichlidae	Africa, Lake Tanganyika
[GenBank:NC_009062]	***Neolamprologus brichardi***	Labroidei Cichlidae	Africa, Lake Tanganyika
[GenBank:NC_013750]	***Oreochromis aureus***	Labroidei Cichlidae	Africa
[GenBank:NC_013663]	***Oreochromis niloticus***	Labroidei Cichlidae	Africa
[GenBank:NC_009057]	***Oreochromis sp. KM_2006***	Labroidei Cichlidae	Africa
[GenBank:NC_011171]	***Tylochromis polylepis***	Labroidei Cichlidae	Africa, Lake Tanganyika
[GenBank:NC_011168]	***Hypselecara temporalis***	Labroidei Cichlidae	South America
[GenBank:NC_009058]	***Astronotus ocellatus***	Labroidei Cichlidae	South America
[GenBank:NC_011169]	***Ptychochromoides katria***	Labroidei Cichlidae	Madagascar
[GenBank:NC_011170]	***Paratilapia polleni***	Labroidei Cichlidae	Madagascar
[GenBank:NC_011177]	***Paretroplus maculatus***	Labroidei Cichlidae	Madagascar
[GenBank:NC_011179]	***Etroplus maculatus***	Labroidei Cichlidae	India
[GenBank:NC_009064]	***Abudefduf vaigiensis***	Labroidei Pomacentridae	Indo-West Pacific
[GenBank:NC_009065]	***Amphiprion ocellaris***	Labroidei Pomacentridae	Indo-West Pacific
[GenBank:NC_009059]	***Cymatogaster aggregata***	Labroidei Embiotocidae	Eastern Pacific
[GenBank:NC_009060]	***Ditrema temminckii***	Labroidei Embiotocidae	Northwest Pacific
[GenBank:NC_012055]	***Pseudolabrus eoethinus***	Labroidei Labridae	Northwest Pacific
[GenBank:NC_009067]	***Pseudolabrus sieboldi***	Labroidei Labridae	Northwest Pacific
[GenBank:NC_010205]	***Pteragogus flagellifer***	Labroidei Labridae	Indo-West Pacific
[GenBank:NC_009066]	***Halichoeres melanurus***	Labroidei Labridae	Indo-West Pacific
[GenBank:NC_009459]	***Parajulis poecilepterus***	Labroidei Labridae	Northwest Pacific

Osmeriformes (outgroup)

Accession number	Name	Classification	Geographic range
[GenBank:NC_013564]	***Alepocephalus agassizii***	Alepocephalidae	Eastern & Western Atlantic
[GenBank:NC_013577]	***Bajacalifornia megalops***	Alepocephalidae	Eastern, Western & Northwest Atlantic; Southeast Pacific; Indian Ocean

Table S2 Sequence similarity of genomic features. Shown are percent and summed position-wise identities of nucleotide and amino acid sequences and the number of gaps occurring in total, as determined by BLAST-based comparison. Recognized and compared coding features: **ND1-6** (NADH dehydrogenase subunit 1-6), **COX1-3** (cytochrome c oxidase subunit 1-3), **ATP6/8** (ATP synthase F0 subunit 6/8), **CYTB** (cytochrome b); values on the right hand side of the **|** symbol refer to amino acid sequences, all other values to nucleotide sequences; **CR** (control region).

TYPE	FEATURE	% ID	NT ID | AA ID	GAPS
**CDS**	**ND1**	96% | 98%	939/ 975 | 319/*324*	0 | 0
	**ND2**	95% | 97%	998/1046 | 337/*348*	0 | 0
	**COX1**	96% | 99%	1539/1596 | 527/*531*	0 | 0
	**COX2**	98% | 99%	676/ 691 | 229/*230*	0 | 0
	**ATP8**	96% | 98%	162/ 168 | 54/ *55*	0 | 0
	**ATP6**	95% | 99%	647/ 683 | 223/*227*	0 | 0
	**COX3**	97% | 99%	758/ 784 | 259/*261*	0 | 0
	**ND3**	97% | 99%	337/ 349 | 115/*116*	0 | 0
	**ND4L**	97% | 100%	287/ 297 | 98/ *98*	0 | 0
	**ND4**	95% | 98%	1314/1381 | 450/*460*	0 | 0
	**ND5**	95% | 98%	1755/1839 | 602/*612*	0 | 0
	**ND6**	96% | 99%	501/ 522 | 172/*173*	0 | 0
	**CYTB**	96% | 99%	1090/1141 | 378/*380*	0 | 0
**rRNA**	**12S**	99%	930/ 943	0
	**16S**	97%	1648/1694	3
**tRNA**	**Phe**	100%	69/69	0
	**Val**	100%	72/72	0
	**Leu-UUA**	99%	73/74	0
	**Ile**	97%	68/70	0
	**Gln**	100%	71/71	0
	**Met**	100%	69/69	0
	**Trp**	97%	70/72	0
	**Ala**	100%	69/69	0
	**Asn**	99%	72/73	0
	**Cys**	100%	66/66	0
	**Tyr**	100%	70/70	0
	**Ser-UCA**	100%	71/71	0
	**Asp**	99%	72/73	0
	**Lys**	100%	74/74	0
	**Gly**	100%	72/72	0
	**Arg**	96%	66/69	0
	**His**	100%	69/69	0
	**Ser-AGC**	97%	65/67	0
	**Leu-CUA**	100%	73/73	0
	**Glu**	97%	67/69	0
	**Thr**	97%	70/72	0
	**Pro**	94%	66/70	0
**CR**	**D-loop**	93%	835/895	5

Table S3A Alignment conditions (with outgroup). Shown are conserved and parsimony informative sites in relation to the total number of sites in the respective alignments. Abbreviations: #, total amount of sites; C, conserved; PI, parsimony informative;

	nucleotides					amino acids				
	# sites	C sites	% C sites	PI sites	% PI sites	# sites	C sites	% C sites	PI sites	% PI sites
**ATP6**	684	326	47.66	318	46.49	227	144	63.44	59	25.99
**ATP8**	168	64	38.10	87	51.79	55	20	36.36	29	52.73
**CYTb**	1143	534	46.72	507	44.36	380	233	61.32	80	21.05
**COX1**	1596	897	56.20	635	39.79	531	421	79.28	76	14.31
**COX2**	693	372	53.68	273	39.39	230	162	70.43	42	18.26
**COX3**	786	430	54.71	325	41.35	261	199	76.25	45	17.24
**ND1**	975	471	48.31	456	46.77	324	221	68.21	72	22.22
**ND2**	1050	408	38.86	556	52.95	349	170	48.71	136	38.97
**ND3**	354	166	46.89	170	48.02	117	77	65.81	31	26.50
**ND4**	1383	582	42.08	714	51.63	460	258	56.09	160	34.78
**ND4L**	297	144	48.48	137	46.13	98	69	70.41	22	22.45
**ND5**	1866	650	34.83	1032	55.31	621	262	42.19	278	44.77
**ND6**	531	188	35.40	295	55.56	176	77	43.75	75	42.61
**12S rRNA**	986	558	56.59	326	33.06					
**16S rRNA**	1792	859	47.94	680	37.95					
**tRNA-Ala-GCA**	69	19	27.54	19	27.54					
**tRNA-Arg-CGA**	69	24	34.78	34	49.28					
**tRNA-Asn-AAC**	73	53	72.60	15	20.55					
**tRNA-Asp-GAC**	74	24	32.43	37	50.00					
**tRNA-Cys-UGC**	68	29	42.65	30	44.12					
**tRNA-Gln-CAA**	72	46	63.89	12	16.67					
**tRNA-Glu-GAA**	70	39	55.71	24	34.29					
**tRNA-Gly-GGA**	73	42	57.53	21	28.77					
**tRNA-His-CAC**	72	27	37.50	26	36.11					
**tRNA-Ile-AUC**	72	39	54.17	25	34.72					
**tRNA-Leu-CUA**	74	65	87.84	5	6.76					
**tRNA-Leu-UUA**	76	36	47.37	27	35.53					
**tRNA-Lys-AAA**	76	44	57.89	19	25.00					
**tRNA-Met-AUG**	70	46	65.71	18	25.71					
**tRNA-Phe-UUC**	69	34	49.28	16	23.19					
**tRNA-Ser-AGC**	73	21	28.77	39	53.42					
**tRNA-Ser-UCA**	71	51	71.83	16	22.54					
**tRNA-Thr-ACA**	73	33	45.21	29	39.73					
**tRNA-Trp-UGA**	75	37	49.33	23	30.67					
**tRNA-Tyr-UAC**	71	38	53.52	25	35.21					
**tRNA-Val-GUA**	73	31	42.47	26	35.62					
**D-loop**	1780	196	11.01	693	38.93					
**data set #1**	11526	5232	45.39	5505	47.76					
**data set #2**	14304	6649	46.48	6511	45.52					
**data set #3**	16084	6845	42.56	7204	44.79					
**data set #3 Gb**	13833	6608	47.77	6240	45.11					

Table S3B Alignment conditions (without outgroup). Shown are conserved and parsimony informative sites in relation to the total number of sites in the respective alignments. Abbreviations: #, total amount of sites; C, conserved; PI, parsimony informative; p-dist, p-distance (with gaps);

		nucleotides							amino acids				
		# sites	C sites	% C sites	PI sites	% PI sites	max p-dist	mean p-dist	# sites	C sites	% C sites	PI sites	% PI sites
**ATP6**		**684**	**330**	**48.25**	**313**	**45.76**	**29.53**	**21.29**	**227**	**145**	**63.88**	**53**	**23.35**
	cp1	228	131	57.46	72	31.58	23.68	12.01					
	cp2	228	194	85.09	23	10.09	8.77	3.79					
	cp3	228	5	2.19	218	95.61	64.04	51.40					
	4fd	79	1	1.27	79	100.00	75.95	54.92					
**ATP8**		**168**	**67**	**39.88**	**81**	**48.21**	**38.69**	**20.31**	**55**	**20**	**36.36**	**23**	**41.82**
	cp1	56	25	44.64	23	41.07	37.50	15.79					
	cp2	56	36	64.29	11	19.64	23.21	6.89					
	cp3	56	6	10.71	47	83.93	66.07	38.25					
	4fd	13	1	7.69	12	92.31	92.31	44.49					
**CYTb**		**1143**	**584**	**51.09**	**482**	**42.17**	**27.47**	**19.49**	**380**	**270**	**71.05**	**66**	**17.37**
	cp1	381	250	65.62	92	24.15	17.85	9.79					
	cp2	381	325	85.30	30	7.87	8.40	3.19					
	cp3	381	9	2.36	360	94.49	60.10	45.48					
	4fd	131	1	0.76	127	96.95	69.47	53.06					
**COX1**		**1596**	**912**	**57.14**	**615**	**38.53**	**25.38**	**18.95**	**531**	**434**	**81.73**	**62**	**11.68**
	cp1	532	405	76.13	95	17.86	12.97	7.57					
	cp2	532	495	93.05	21	3.95	5.45	3.06					
	cp3	325	2	0.62	319	98.15	60.71	46.22					
	4fd	230	5	2.17	218	94.78	68.70	52.57					
**COX2**		**693**	**382**	**55.12**	**262**	**37.81**	**24.68**	**16.61**	**230**	**167**	**72.61**	**37**	**16.09**
	cp1	231	163	70.56	46	19.91	15.58	6.83					
	cp2	231	209	90.48	10	4.33	6.06	1.54					
	cp3	231	10	4.33	206	89.18	60.17	41.46					
	4fd	119	0	0.00	116	97.48	70.59	52.30					
**COX3**		**786**	**441**	**56.11**	**308**	**39.19**	**24.55**	**17.07**	**261**	**205**	**78.54**	**40**	**15.33**
	cp1	262	192	73.28	56	21.37	13.74	7.17					
	cp2	262	234	89.31	19	7.25	4.96	2.33					
	cp3	262	15	5.73	233	88.93	57.63	41.72					
	4fd	104	4	3.85	97	93.27	69.23	49.58					
**ND1**		**975**	**484**	**49.64**	**444**	**45.54**	**30.36**	**20.63**	**324**	**227**	**70.06**	**65**	**20.06**
	cp1	325	197	60.62	97	29.85	19.38	10.86					
	cp2	325	285	87.69	28	8.62	7.08	2.95					
	cp3	325	2	0.62	319	98.15	67.69	48.08					
	4fd	121	1	0.83	121	100.00	76.03	52.13					
**ND2**		**1047**	**430**	**41.07**	**514**	**49.09**	**31.90**	**22.67**	**348**	**178**	**51.15**	**117**	**33.62**
	cp1	349	164	46.99	129	36.96	23.21	14.33					
	cp2	349	261	74.79	51	14.61	12.03	5.33					
	cp3	349	5	1.43	334	95.70	63.32	48.34					
	4fd	119	0	0.00	116	97.48	70.59	52.30					
**ND3**		**354**	**168**	**47.46**	**166**	**46.89**	**29.66**	**22.08**	**117**	**79**	**67.52**	**30**	**25.64**
	cp1	118	68	57.63	36	30.51	20.34	12.72					
	cp2	118	99	83.90	16	13.56	10.17	5.21					
	cp3	118	1	0.85	115	97.46	65.25	48.31					
	4fd	39	0	0.00	39	100.00	82.05	52.39					
**ND4**		**1383**	**596**	**43.09**	**686**	**49.60**	**31.89**	**22.75**	**460**	**266**	**57.83**	**147**	**31.96**
	cp1	461	239	51.84	172	37.31	25.60	14.61					
	cp2	461	349	75.70	73	15.84	14.75	6.08					
	cp3	461	8	1.74	441	95.66	63.34	47.55					
	4fd	140	0	0.00	136	97.14	72.86	52.98					
**ND4L**		**297**	**152**	**51.18**	**130**	**43.77**	**28.28**	**19.79**	**98**	**70**	**71.43**	**19**	**19.39**
	cp1	99	60	60.61	31	31.31	23.23	10.69					
	cp2	99	88	88.89	9	9.09	8.08	2.85					
	cp3	99	4	4.04	90	90.91	64.65	45.85					
	4fd	42	0	0.00	41	97.62	73.81	50.28					
**ND5**		**1866**	**662**	**35.48**	**1006**	**53.91**	**41.26**	**25.30**	**621**	**266**	**42.83**	**265**	**42.67**
	cp1	622	271	43.57	275	44.21	36.82	18.29					
	cp2	622	384	61.74	145	23.31	24.12	9.89					
	cp3	622	7	1.13	586	94.21	64.63	47.73					
	4fd	170	2	1.18	161	94.71	71.18	52.13					
**ND6**		**531**	**193**	**36.35**	**285**	**53.67**	**40.11**	**25.96**	**176**	**82**	**46.59**	**70**	**39.77**
	cp1	177	75	42.37	79	44.63	36.16	19.05					
	cp2	177	116	65.54	38	21.47	21.47	9.67					
	cp3	177	2	1.13	168	94.92	67.80	49.16					
	4fd	60	0	0.00	57	95.00	83.33	56.46					

Table S3B Alignment conditions (without outgroup) - continued.

nucleotides

	# sites	C sites	% C sites	PI sites	% PI sites	max p-dist	mean p-dist
**12S rRNA**	979	572	58.43	298	30.44	24.72	15.95
**16S rRNA**	1782	888	49.83	609	34.18	27.44	20.22
**tRNA-Ala-GCA**	69	20	28.99	17	24.64	57.97	11.32
**tRNA-Arg-CGA**	70	32	45.71	27	38.57	37.14	15.57
**tRNA-Asn-AAC**	73	55	75.34	11	15.07	13.70	6.82
**tRNA-Asp-GAC**	74	26	35.14	32	43.24	40.54	19.72
**tRNA-Cys-UGC**	68	33	48.53	29	42.65	38.24	20.19
**tRNA-Gln-CAA**	72	50	69.44	8	11.11	18.06	5.91
**tRNA-Glu-GAA**	70	41	58.57	21	30.00	24.29	11.85
**tRNA-Gly-GGA**	73	42	57.53	18	24.66	28.77	11.72
**tRNA-His-CAC**	70	30	42.86	25	35.71	35.71	14.73
**tRNA-Ile-AUC**	71	45	63.38	19	26.76	23.94	11.27
**tRNA-Leu-CUA**	74	67	90.54	4	5.41	6.76	3.15
**tRNA-Leu-UUA**	75	40	53.33	23	30.67	28.00	13.73
**tRNA-Lys-AAA**	76	49	64.47	16	21.05	23.68	11.62
**tRNA-Met-AUG**	70	48	68.57	16	22.86	21.43	8.23
**tRNA-Phe-UUC**	69	39	56.52	15	21.74	24.64	9.54
**tRNA-Pro-CCA**	72	38	52.78	26	36.11	29.17	14.82
**tRNA-Ser-AGC**	73	21	28.77	37	50.68	49.32	27.69
**tRNA-Ser-UCA**	71	53	74.65	15	21.13	16.90	7.60
**tRNA-Thr-ACA**	73	40	54.79	22	30.14	32.88	13.58
**tRNA-Trp-UGA**	75	39	52.00	20	26.67	33.33	14.37
**tRNA-Tyr-UAC**	71	44	61.97	21	29.58	25.35	14.30
**tRNA-Val-GUA**	73	35	47.95	23	31.51	31.51	13.67
**D-loop**	1694	167	9.86	621	36.66	79.04	64.54
**D-loop Gb**	777	135	17.37	448	57.66	58.30	33.44

	12S rRNA	ND6
Abudefduf vaigiensis	11.03	20.90
Amphiprion ocellaris	15.12	19.77
Astronotus ocellatus	11.03	17.33
Cymatogaster aggregata	12.16	24.48
Ditrema temminckii	13.07	23.92
Etroplus maculatus	12.67	18.64
Halichoeres melanurus	19.82	33.71
Hypselecara temporalis	12.36	18.08
Neolamprologus brichardi	9.09	13.37
Oreochromis aureus	7.97	10.73
Oreochromis niloticus	7.56	11.86
Oreochromis sp. KM2006	7.66	11.49
Parajulis poecilepterus	19.71	31.83
Paratilapia polleni	10.73	18.27
Paretroplus maculatus	10.52	16.01
Petrochromis trewavasae	7.25	9.42
Pseudolabrus eoethinus	19.82	31.26
Pseudolabrus sieboldi	19.71	29.94
Pteragogus flagellifer	19.00	31.26
Ptychochromoides katria	11.13	21.66
Tropheus duboisi	7.87	10.36
Tropheus moorii	7.66	11.30
Tylochromis polylepis	10.83	15.25

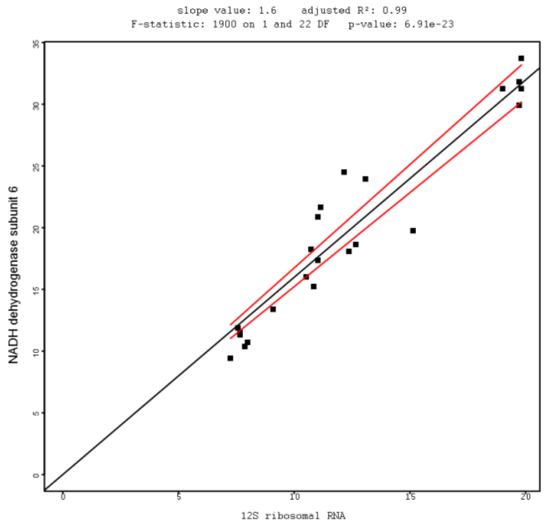

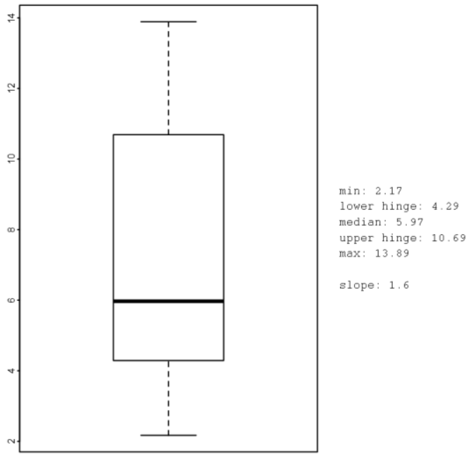

Figure S1 Example of a regression analysis result. For each gene a linear least squares regression was calculated against a reference gene. In this example, pairwise distances (each species-specific sequence against the consensus sequence) for the ND6 genes were related to the distances for the reference 12S rRNA genes. Dots represent distance value pairs per species; red lines indicate the confidence region. The slope value of the regression line is presented in the bar plot in **Figure 3**. Boxplots were used to examine the distribution of differences in the distance value pairs (i.e. the rate gradient or direction, showing which one of the two genes exhibits more normalized substitutions). In the case of ND6, in each observed species there were more substitutions in ND6 than in the 12S rRNA (only positive values). In tRNA vs. 12S rRNA comparisons the zero was crossed in several cases, hence, for certain species the direction of which gene evolves relatively faster is inverse to that stated for the general trend.

Table S4A Overview of linear regression statistics (p-distance based). Shown are adjusted R^2 values and p-values from F-statistics. R^2 is interpretable as the proportion of the total variability of the distance pairs that is accounted for by the linear model; as each additional model parameter (additional data point in the distance vector, i.e. an additional species) increases R^2, even if it has no statistical power, the adjusted version was applied (taking model complexity into account). F is the ratio of the variance explained by the parameters in the linear model and the residual or unexplained variance; p-values are calculated as the probability of achieving an F that large under the null hypothesis of 'no effect' (a bad model), from an F-distribution with degrees of freedom related to the number of observations/parameters**.** Regression results for rRNAs, coding-genes and the D-loop may be regarded as reliable; however, some distance pair scatter plots exhibited rather broad distributions lowering R^2 values and hampering a clear placement of the regression line, they are tagged (*).

feature		coefficient	adjusted R^2	p-value (F-statistic)	
**ATP6**	4-fold	3.22	0.90	9.96E-13	
**ATP8**		2.50	0.79	4.19E-09	*
**CYTB**		3.15	0.92	1.42E-13	
**COX1**		3.16	0.94	3.96E-15	
**COX2**		2.83	0.76	2.05E-08	*
**COX3**		2.94	0.91	5.72E-13	
**ND1**		3.07	0.91	2.91E-13	
**ND2**		2.69	0.68	3.79E-07	*
**ND3**		2.92	0.84	2.59E-10	
**ND4**		3.05	0.87	1.70E-11	
**ND4L**		2.92	0.90	1.28E-12	
**ND5**		3.11	0.93	9.30E-15	
**ND6**		3.53	0.97	8.63E-18	
**AVG**		**3.01**			
**ATP6**	1st	0.65	0.95	7.92E-16	
**ATP8**		0.92	0.83	2.90E-09	
**CYTB**		0.52	0.95	8.11E-16	
**COX1**		0.43	0.94	2.45E-15	
**COX2**		0.37	0.91	2.02E-13	
**COX3**		0.38	0.89	2.79E-12	
**ND1**		0.61	0.97	9.20E-18	
**ND2**		0.77	0.95	6.45E-16	
**ND3**		0.68	0.93	1.53E-14	
**ND4**		0.84	0.97	3.02E-18	
**ND4L**		0.61	0.90	6.66E-13	
**ND5**		0.94	0.98	1.91E-17	
**ND6**		1.17	0.95	2.64E-16	
**AVG**		**0.68**			
**ATP6**	2nd	0.24	0.82	6.46E-10	
**ATP8**		0.41	0.76	4.15E-08	*
**CYTB**		0.16	0.91	2.01E-12	
**COX1**		0.19	0.87	8.00E-11	
**COX2**		0.10	0.62	1.95E-04	*
**COX3**		0.14	0.90	1.45E-10	
**ND1**		0.20	0.93	8.53E-11	
**ND2**		0.29	0.94	9.03E-15	
**ND3**		0.30	0.81	1.50E-09	
**ND4**		0.37	0.82	7.76E-10	
**ND4L**		0.19	0.85	1.31E-09	
**ND5**		0.61	0.86	3.90E-11	
**ND6**		0.58	0.95	4.26E-16	
**AVG**		**0.29**			

feature		coefficient	adjusted R^2	p-value (F-statistic)	
**ATP6**	3rd	2.86	0.97	5.76E-18	
**ATP8**		2.27	0.94	1.71E-15	
**CYTB**		2.70	0.96	1.32E-16	
**COX1**		2.75	0.95	2.70E-16	
**COX2**		2.50	0.97	2.31E-18	
**COX3**		2.48	0.97	4.41E-18	
**ND1**		2.90	0.97	1.34E-18	
**ND2**		2.92	0.98	4.93E-20	
**ND3**		2.92	0.96	2.90E-17	
**ND4**		2.89	0.98	2.05E-21	
**ND4L**		2.74	0.95	1.27E-15	
**ND5**		2.88	0.98	7.24E-20	
**ND6**		3.04	0.97	4.04E-19	
**AVG**		**2.76**			
**ATP6**	codon	1.25	0.98	1.79E-20	
**ATP8**		1.19	0.93	1.42E-14	
**CYTB**		1.14	0.96	1.06E-16	
**COX1**		1.12	0.96	4.45E-17	
**COX2**		0.98	0.97	3.00E-18	
**COX3**		1.00	0.97	3.84E-18	
**ND1**		1.23	0.98	1.92E-20	
**ND2**		1.33	0.98	5.47E-20	
**ND3**		1.30	0.96	1.67E-17	
**ND4**		1.37	0.99	8.14E-26	
**ND4L**		1.17	0.97	7.06E-19	
**ND5**		1.53	0.99	4.41E-23	
**ND6**		1.60	0.99	6.91E-23	
**AVG**		**1.25**			
**tRNA-Ala-GCA**		0.40	0.85	5.61E-10	
**tRNA-Arg-CGA**		0.91	0.87	2.64E-11	
**tRNA-Asn-AAC**		0.36	0.73	1.19E-07	*
**tRNA-Asp-GAC**		1.18	0.93	2.29E-14	
**tRNA-Cys-UGC**		1.23	0.96	2.40E-17	
**tRNA-Gln-CAA**		0.32	0.70	2.19E-07	*
**tRNA-Glu-GAA**		0.77	0.89	1.95E-12	
**tRNA-Gly-GGA**		0.71	0.82	8.07E-10	
**tRNA-His-CAC**		0.91	0.87	2.70E-11	
**tRNA-Ile-AUC**		0.64	0.90	9.45E-13	
**tRNA-Leu-CUA**		0.19	0.70	2.12E-07	*
**tRNA-Leu-UUA**		0.87	0.96	3.88E-17	
**tRNA-Lys-AAA**		0.70	0.92	4.96E-14	
**tRNA-Met-AUG**		0.50	0.85	6.98E-11	
**tRNA-Phe-UUC**		0.62	0.86	1.97E-09	
**tRNA-Pro-CCA**		0.79	0.82	9.30E-10	
**tRNA-Ser-AGC**		1.70	0.95	3.67E-16	
**tRNA-Ser-UCA**		0.45	0.84	1.43E-10	
**tRNA-Thr-ACA**		0.79	0.84	2.14E-10	
**tRNA-Trp-UGA**		0.87	0.92	1.35E-13	
**tRNA-Tyr-UAC**		0.83	0.87	2.82E-11	
**tRNA-Val-GUA**		0.80	0.91	2.58E-13	
**AVG**		0.75			
**12S rRNA**		1.00	1.00	0.00E+00	ref
**16S rRNA**		1.23	0.97	5.90E-19	
**AVG**		**1.12**			
**D-loop Gb**		1.97	0.95	3.44E-16	
**D-loop**		4.38	0.91	5.71E-13	

Table S4B Overview of Bayesian-estimated mean rates. Shown are the mean rates (see **Figure S5** for the definition) along with the median and the lower and upper bound of the highest posterior density (HPD) interval (the credible set that contains 95% of the sampled values) for all genes or partitions.

feature		mean rate	median rate	95% HPD lower	95% HPD upper
**ATP6**	4-fold	50.12	33.46	7.40	144.82
**ATP8**		19.67	15.47	4.43	44.81
**CYTB**		53.16	46.46	16.95	100.77
**COX1**		16.31	15.58	9.74	23.83
**COX2**		74.74	59.30	16.26	165.01
**COX3**		10.72	9.69	5.56	17.93
**ND1**		29.82	27.40	14.47	49.85
**ND2**		35.99	30.78	13.53	67.99
**ND3**		48.23	37.35	10.86	115.31
**ND4**		44.73	38.07	15.15	85.51
**ND4L**		10.36	9.72	5.41	16.65
**ND5**		27.54	24.74	12.34	50.56
**ND6**		24.58	20.56	8.63	49.38
**AVG**		**13.34**			
**ATP6**	1st	1.32	1.27	0.77	1.97
**ATP8**		1.49	1.42	0.77	2.37
**CYTB**		0.70	0.69	0.48	0.94
**COX1**		0.40	0.40	0.28	0.54
**COX2**		0.31	0.31	0.23	0.40
**COX3**		0.37	0.36	0.25	0.49
**ND1**		0.84	0.83	0.56	1.15
**ND2**		1.01	1.00	0.74	1.29
**ND3**		0.79	0.77	0.49	1.14
**ND4**		1.06	1.05	0.78	1.36
**ND4L**		0.61	0.60	0.38	0.84
**ND5**		1.57	1.55	1.15	2.00
**ND6**		1.94	1.90	1.23	2.76
**AVG**		0.95			
**ATP6**	2nd	0.19	0.18	0.11	0.27
**ATP8**		0.58	0.55	0.25	0.99
**CYTB**		0.13	0.13	0.08	0.18
**COX1**		0.10	0.09	0.05	0.14
**COX2**		0.06	0.06	0.03	0.09
**COX3**		0.08	0.08	0.05	0.13
**ND1**		0.14	0.13	0.08	0.19
**ND2**		0.30	0.30	0.21	0.41
**ND3**		0.29	0.28	0.15	0.44
**ND4**		0.29	0.29	0.21	0.38
**ND4L**		0.20	0.18	0.07	0.35
**ND5**		0.72	0.71	0.51	0.94
**ND6**		0.58	0.56	0.35	0.86
**AVG**		0.28			

feature		mean rate	median rate	95% HPD lower	95% HPD upper
**ATP6**	3rd	12.13	11.88	8.18	16.73
**ATP8**		6.95	6.52	3.55	11.36
**CYTB**		13.89	13.76	9.67	18.36
**COX1**		7.40	7.35	5.43	9.31
**COX2**		9.08	8.90	5.97	12.52
**COX3**		10.72	10.45	7.19	14.89
**ND1**		10.69	10.55	7.60	13.98
**ND2**		11.41	11.19	7.97	15.37
**ND3**		10.50	10.28	6.93	14.33
**ND4**		11.20	11.11	8.17	14.36
**ND4L**		7.74	7.56	5.03	10.72
**ND5**		10.97	10.81	7.86	14.46
**ND6**		26.72	25.25	12.82	44.43
**AVG**		11.49			
**ATP6**	codon	3.85	3.8	2.77	4.97
**ATP8**		2.59	2.52	1.57	3.72
**CYTB**		3.47	3.44	2.59	4.39
**COX1**		2.17	2.15	1.65	2.71
**COX2**		2.45	2.41	1.77	3.25
**COX3**		2.47	2.44	1.83	3.18
**ND1**		3.28	3.24	2.49	4.16
**ND2**		2.98	2.96	2.29	3.74
**ND3**		3.75	3.66	2.5	5.16
**ND4**		2.99	2.96	2.3	3.73
**ND4L**		2.64	2.6	1.79	3.56
**ND5**		3.12	3.09	2.44	3.86
**ND6**		5.23	5.12	3.52	7.17
**AVG**		3.14			
**tRNA-Ala-GCA**		5.10	3.95	0.94	11.86
**tRNA-Arg-CGA**		0.88	0.85	0.51	1.29
**tRNA-Asn-AAC**		0.30	0.29	0.15	0.47
**tRNA-Asp-GAC**		1.69	1.62	0.92	2.62
**tRNA-Cys-UGC**		1.23	1.18	0.66	1.94
**tRNA-Gln-CAA**		0.42	0.4	0.19	0.73
**tRNA-Glu-GAA**		0.67	0.64	0.33	1.04
**tRNA-Gly-GGA**		1.09	0.97	0.43	2.07
**tRNA-His-CAC**		1.39	1.31	0.7	2.22
**tRNA-Ile-AUC**		2.14	1.73	0.6	4.84
**tRNA-Leu-CUA**		0.20	0.17	0.06	0.38
**tRNA-Leu-UUA**		0.76	0.73	0.4	1.17
**tRNA-Lys-AAA**		0.52	0.5	0.29	0.8
**tRNA-Met-AUG**		0.32	0.31	0.18	0.46
**tRNA-Phe-UUC**		0.56	0.54	0.31	0.87
**tRNA-Pro-CCA**		0.93	0.89	0.47	1.46
**tRNA-Ser-AGC**		1.28	1.25	0.81	1.82
**tRNA-Ser-UCA**		0.39	0.38	0.2	0.6
**tRNA-Thr-ACA**		1.31	1.23	0.63	2.12
**tRNA-Trp-UGA**		0.85	0.81	0.45	1.31
**tRNA-Tyr-UAC**		1.05	0.92	0.4	2.04
**tRNA-Val-GUA**		0.78	0.75	0.44	1.15
**AVG**		0.84			
**12S rRNA**		1.01	1.01	0.88	1.16
**16S rRNA**		1.27	1.26	0.99	1.56
**AVG**		1.14			
**D-loop**		3.21	3.19	2.54	3.93
**D-loop Gb**		2.42	2.4	1.89	2.95

amino acid substitution models

Gene	Model	ΔAIC	-lnL
**CYTB**	**MtMam+G+F**	0.0	-3301.91
	MtMam+I+G+F	1.8	-3301.81
	MtREV+G+F	12.8	-3308.31
**COX1**	**MtREV+I+G+F**	0.0	-3067.42
	CpREV+I+G+F	4.3	-3069.56
	MtREV+G+F	8.7	-3072.76
**COX2**	**MtMam+G+F**	0.0	-1457.21
	MtMam+G	0.5	-1476.47
	MtMam+I+G+F	3.2	-1457.8
**COX3**	**MtREV+I+G+F**	0.0	-1708.45
	MtREV+G+F	3.0	-1710.94
	MtMam+I+G+F	6.2	-1711.56
**ATP6**	**MtMam+I+G+F**	0.0	-1939.17
	MtMam+G+F	2.2	-1941.25
	FLU+I+G+F	9.7	-1944.01
**ATP8**	MtMam+I+G	0.0	-751.21
	**MtMam+G** *	0.0	-752.22
	MtREV+G	3.6	-753.99
**ND1**	**MtMam+I+G+F**	0.0	-2604.52
	MtMam+G+F	1.2	-2606.15
	MtMam+I+G	11.0	-2629.04
**ND2**	**MtMam+G+F**	0.0	-4222.76
	MtMam+I+G+F	0.3	-4221.90
	MtMam+G	43.5	-4263.51
**ND3**	**MtMam+G**	0.0	-1112.99
	MtMam+I+G	2.5	-1113.21
	MtMam+G+F	22.8	-1105.36
**ND4L**	**MtMam+G+F**	0.0	-731.01
	MtMam+I+G+F *	0.2	-730.12
	FLU+G+F	4.8	-733.39
**ND4**	**MtMam+I+G**	0.0	-5124.29
	MtMam+G	2.1	-5126.35
	MtMam+G+F	11.8	-5112.17
**ND5**	**MtMam+I+G**	0.0	-8892.98
	MtMam+I+G+F	1.0	-8874.48
	MtMam+G	1.9	-8894.9
**ND6**	HIVb+I+G+F	0.0	-2248.57
	MtMam+G+F	2.3	-2250.73
	HIVb+G+F	2.6	-2250.86
	**MtREV+G+F** (boxed)	0.0	-2255.81
	MtREV+I+G+F (boxed)	0.5	-2255.07
	MtMam+G+F (boxed)	0.6	-2256.12
**CDS cc**	**MtMam+I+G+F**	0.0	-38935.34
	MtREV+I+G+F	173.3	-39022.00
	MtREV+G+F	221.0	-39046.82

nucleotide substitution models

Gene	Model	p	ΔAIC	-lnL
**12S rRNA**	TIM2+I+G	56	0.0	7339.40
	**GTR+I+G**	58	1.9	7338.37
	TIM2+G	55	15.6	7348.18
	GTR+G	57	17.6	7347.21
	TPM2uf+I+G	55	37.9	7359.33
	TVM+I+G	57	39.9	7358.36
**16S rRNA**	TIM2+I+G	56	0.0	15551.39
	**GTR+I+G**	58	0.7	15549.72
	TIM2+G	55	12.0	15558.37
	GTR+G	57	12.7	15556.73
	TVM+I+G	57	85.4	15593.06
	TPM2uf+I+G	55	87.4	15596.09
**ATP6**	TIM1+I+G	56	0.0	7923.94
	TIM3+I+G	56	1.3	7924.58
	**GTR+I+G**	58	2.5	7923.20
	TrN+I+G	55	2.7	7926.27
	TIM2+I+G	56	3.0	7925.46
	HKY+I+G	54	4.8	7928.36
**ATP8**	TrN+G	54	0.0	1857.22
	TIM2+G	55	0.8	1856.60
	**HKY+I+G**	54	2.2	1858.31
	TPM3uf+I+G	55	3.3	1857.87
	HKY+G	53	3.9	1860.18
	TPM2uf+I+G	55	4.1	1858.29
**CYTB**	TIM3+I+G	56	0.0	12768.29
	**GTR+I+G**	58	3.4	12767.98
	TPM3uf+I+G	55	3.6	12771.07
	TIM2+I+G	56	5.4	12770.97
	TPM1uf+I+G	55	5.5	12772.02
	TPM2uf+I+G	55	5.5	12772.06
**COX1**	TVM+I+G	57	0.0	15708.92
	**GTR+I+G**	58	0.8	15708.34
	TPM3uf+I+G	55	6.1	15713.98
	TIM3+I+G	56	8.1	15713.97
	TPM2uf+I+G	55	10.2	15716.00
	TIM2+I+G	56	11.2	15715.54
**COX2**	TPM1uf+G	54	0.0	6509.34
	TPM1uf+I+G	55	0.2	6508.42
	TIM1+I+G	56	0.7	6507.66
	TIM1+G	55	1.6	6509.12
	TVM+I+G	57	1.9	6507.30
	**GTR+I+G**	58	3.9	6507.29
**COX3**	TPM3uf+I+G	55	0.0	7742.41
	TVM+I+G	57	1.5	7741.17
	TIM3+I+G	56	1.6	7742.22
	**GTR+I+G**	58	3.5	7741.17
	TPM2uf+I+G	55	10.4	7747.59
	HKY+I+G	54	10.9	7748.86
**ND1**	TrN+I+G	55	0.0	11053.26
	TIM3+I+G	56	1.0	11052.77
	TIM1+I+G	56	1.6	11053.06
	TIM2+I+G	56	1.6	11053.07
	**GTR+I+G**	58	4.6	11052.57
	HKY+I+G	54	16.5	11062.49

Gene	Model	p	ΔAIC	-lnL
**ND2**	**GTR+I+G**	58	0.0	13282.69
	TIM3+I+G	56	11.9	13290.62
	GTR+G	57	20.0	13293.71
	TIM2+I+G	56	25.6	13297.49
	TVM+I+G	57	27.0	13297.17
	TPM3uf+I+G	55	27.6	13299.51
**ND3**	TIM2+I+G	56	0.0	4109.03
	TIM1+I+G	56	1.9	4109.99
	**GTR+I+G**	58	5.0	4109.55
	TrN+I+G	55	5.2	4112.62
	TIM3+I+G	56	5.5	4111.78
	TPM1uf+I+G	55	21.3	4120.69
**ND4**	TIM3+I+G	56	0.0	16946.70
	**GTR+I+G**	58	0.3	16944.87
	TIM1+I+G	56	5.1	16949.23
	TIM2+I+G	56	7.7	16950.54
	TrN+I+G	55	10.6	16952.99
	TPM3uf+I+G	55	40.4	16967.90
**ND4L**	TIM3+I+G	56	0.0	3242.87
	TPM3uf+I+G	55	0.8	3244.24
	**GTR+I+G**	58	1.9	3241.81
	TVM+I+G	57	3.9	3243.79
	TrN+I+G	55	8.8	3248.27
	TIM1+I+G	56	9.5	3247.63
**ND5**	TIM3+I+G	56	0.0	23906.81
	TPM3uf+I+G	55	0.7	23908.16
	**GTR+I+G**	58	3.4	23906.50
	TVM+I+G	57	3.6	23907.61
	TIM1+I+G	56	6.4	23910.02
	TPM1uf+I+G	55	8.9	23912.26
**ND6**	**GTR+I+G**	58	0.0	6421.86
	TIM1+I+G	56	0.1	6423.90
	TIM3+I+G	56	0.2	6423.98
	TPM1uf+I+G	55	2.6	6426.15
	TVM+I+G	57	5.1	6425.40
	TPM3uf+I+G	55	5.2	6427.48
**D-loop**	TPM3uf+G	54	0.0	16372.77
	TIM3+G	55	0.4	16371.98
	TPM3uf+I+G	55	1.2	16372.35
	TIM3+I+G	56	1.6	16371.57
	**GTR+G**	57	1.9	16370.70
	TVM+G	56	2.1	16371.83
**CDS cc**	**GTR+I+G**	58	0.0	134175.66
	TIM3+I+G	56	22.1	134188.69
	TVM+I+G	57	22.3	134187.82
	TPM3uf+I+G	55	50.7	134204.01
	TrN+I+G	55	156.6	134256.95
	TIM2+I+G	56	156.8	134256.08
**CDS|rRNA cc**	TVM+I+G	57	0.0	162809.38
	**GTR+I+G**	58	1.3	162809.01
	TIM3+I+G	56	83.8	162852.27
	TPM3uf+I+G	55	86.9	162854.84
	TPM2uf+I+G	55	137.5	162880.14
	TIM2+I+G	56	139.1	162879.91

Gene	Model	p	ΔAIC/ΔAICc	-lnL
**CDS|rRNA| D-loop cc**	**GTR+I+G**	58	0.0	182119.44
	TVM+I+G	57	0.3	182120.60
	TIM3+I+G	56	107.8	182175.34
	TPM3uf+I+G	55	110.6	182177.75
	TPM2uf+I+G	55	189.2	182217.02
	TIM2+I+G	56	189.7	182216.31
**tRNA-Ala-GCA**	**K80**	45	0.0	400.85
**tRNA-Arg-CGA**	**K80+G**	46	0.0	489.76
**tRNA-Asn-AAC**	**K80+I**	46	0.0	254.99
**tRNA-Asp-GAC**	**K80+G**	46	0.0	595.01
**tRNA-Cys-UGC**	**K80+G**	46	0.0	553.26
**tRNA-Gln-CAA**	**K80**	45	0.0	263.17
**tRNA-Glu-GAA**	**K80+G**	46	0.0	369.14
**tRNA-Gly-GGA**	**K80+G**	46	0.0	395.83
**tRNA-His-CAC**	**K80+G**	46	0.0	448.58
**tRNA-Ile-AUC**	**K80+G**	46	0.0	395.47
**tRNA-Leu-CUA**	**K80**	45	0.0	150.14
tRNA-Leu-UUA (grey)	TPM2+G	47	0.0	445.47
**tRNA-Leu-UUA**	**K80+G**	46	1.5	453.56
**tRNA-Lys-AAA**	**K80+G**	46	0.0	369.14
**tRNA-Met-AUG**	**JC+I**	45	0.0	304.70
**tRNA-Phe-UUC**	**K80+G**	46	0.0	344.75
**tRNA-Pro-CCA**	**K80+G**	46	0.0	450.96
**tRNA-Ser-AGC**	**K80+G**	46	0.0	623.07
**tRNA-Ser-UCA**	**K80+I**	46	0.0	292.72
**tRNA-Thr-ACA**	**K80+G**	46	0.0	471.08
**tRNA-Trp-UGA**	**K80+G**	46	0.0	425.22
**tRNA-Tyr-UAC**	**K80+G**	46	0.0	444.70
**tRNA-Val-GUA**	**K80+G**	46	0.0	425.33

Table S5A AIC and AICc selected substitution models (with outgroup). Best nucleotide substitution models were determined out of 88 candidate models (11 substitution schemes, G, I and F) with 8 rate categories for the gamma distribution; a maximum likelihood tree was used as base tree for likelihood calculations, SPR as tree topology search option. Best amino acid substitution models were determined out of all 120 available models (15 matrices, I, G, I+G, and F) with 8 rate categories; starting topology for likelihood calculations was either a fixed BIONJ JTT tree or a maximum likelihood tree (the top 3 scoring models of the latter are shown). Starting topology had an influence on selected models but mostly only on the ordering of the top 3, except for ND6 for which the whole fixed BIONJ top 3 are also given (boxed). In cases were the starting topology had an influence on the selected model, the fixed BIONJ top model is marked with an asterisk (*). Eventually selected models for calculations are highlighted (bold). Models were rated according to the AIC (for tRNA sequences the AIC corrected for small sample size (AICc) was applied). ΔAIC = AIC difference to top scoring model; p = number of model parameters; -lnL = log-likelihood, cc = concatenated;

Table S5B Selected substitution models. Shown are the model parameters for the AIC-selected and the actually applied (**bold**) substitution model per gene and concatenation (cc), respectively.

Sequence	Model	Partition	-lnL	p	ΔAIC	fA	fC	fG	fT	ti/tv	rAC	rAG	rAT	rCG	rCT	rGT	p-inv	alpha
**12S rRNA**	TIM2+I+G	010232	7339.40	56	0.00	0.33	0.24	0.20	0.22	-	5.03	12.13	5.03	1.00	31.82	1.00	0.37	0.53
	**GTR+I+G**	**012345**	**7338.37**	**58**	**1.94**	**0.33**	**0.25**	**0.20**	**0.22**	**-**	**6.23**	**16.54**	**7.63**	**1.69**	**43.42**	**1.00**	**0.38**	**0.54**
**16S rRNA**	TIM2+I+G	010232	15551.39	56	0.00	0.36	0.26	0.18	0.20	-	7.10	13.58	7.10	1.00	38.55	1.00	0.27	0.56
	**GTR+I+G**	**012345**	**15549.72**	**58**	**0.67**	**0.36**	**0.26**	**0.18**	**0.20**	**-**	**4.85**	**8.99**	**4.41**	**0.43**	**25.21**	**1.00**	**0.27**	**0.56**
**ATP6**	TIM1+I+G	012230	7923.94	56	0.00	0.30	0.37	0.09	0.24	-	1.00	21.17	1.85	1.85	12.68	1.00	0.38	0.52
	**GTR+I+G**	**012345**	**7923.20**	**58**	**2.53**	**0.30**	**0.38**	**0.09**	**0.24**	**-**	**0.57**	**13.65**	**1.20**	**1.08**	**8.08**	**1.00**	**0.38**	**0.52**
**ATP8**	TrN+G	010020	1857.22	54	0.00	0.29	0.37	0.08	0.27	-	1.00	9.41	1.00	1.00	7.06	1.00	-	0.32
	**HKY+I+G**	**010010**	**1858.31**	**54**	**2.19**	**0.30**	**0.36**	**0.09**	**0.25**	**3.45**	**-**	**-**	**-**	**-**	**-**	**-**	**0.23**	**0.61**
**CYTB**	TIM3+I+G	012032	12768.29	56	0.00	0.29	0.39	0.09	0.23	-	0.60	6.14	1.00	0.60	8.79	1.00	0.31	0.41
	**GTR+I+G**	**012345**	**12767.98**	**58**	**3.39**	**0.28**	**0.39**	**0.10**	**0.23**	**-**	**0.67**	**6.01**	**1.03**	**0.52**	**9.12**	**1.00**	**0.31**	**0.42**
**COX1**	TVM+I+G	012314	15708.92	57	0.00	0.28	0.32	0.12	0.27	-	0.90	8.70	1.49	0.46	8.70	1.00	0.52	0.94
	**GTR+I+G**	**012345**	**15708.34**	**58**	**0.84**	**0.29**	**0.32**	**0.13**	**0.27**	**-**	**0.93**	**8.26**	**1.58**	**0.45**	**9.50**	**1.00**	**0.52**	**0.93**
**COX2**	TPM1uf+G	012210	6509.34	54	0.00	0.34	0.31	0.10	0.25	-	1.00	22.34	2.24	2.24	22.34	1.00	-	0.16
	**GTR+I+G**	**012345**	**6507.29**	**58**	**3.90**	**0.34**	**0.31**	**0.10**	**0.25**	**-**	**1.43**	**26.79**	**3.65**	**2.27**	**27.98**	**1.00**	**0.30**	**0.31**
**COX3**	TPM3uf+I+G	012012	7742.41	55	0.00	0.31	0.36	0.10	0.23	-	0.44	6.33	1.00	0.44	6.33	1.00	0.47	0.62
	**GTR+I+G**	**012345**	**7741.17**	**58**	**3.52**	**0.31**	**0.35**	**0.11**	**0.23**	**-**	**0.60**	**6.45**	**1.10**	**0.29**	**6.70**	**1.00**	**0.48**	**0.66**
**ND1**	TrN+I+G	010020	11053.26	55	0.00	0.30	0.36	0.09	0.25	-	1.00	14.74	1.00	1.00	7.31	1.00	0.39	0.59
	**GTR+I+G**	**012345**	**11052.57**	**58**	**4.62**	**0.30**	**0.37**	**0.09**	**0.25**	**-**	**0.71**	**12.31**	**0.85**	**0.84**	**5.89**	**1.00**	**0.39**	**0.59**
**ND2**	**GTR+I+G**	**012345**	**13282.69**	**58**	**0.00**	**0.31**	**0.39**	**0.08**	**0.22**	**-**	**0.22**	**4.00**	**0.36**	**0.30**	**1.76**	**1.00**	**0.28**	**0.60**
**ND3**	TIM2+I+G	010232	4109.03	56	0.00	0.29	0.36	0.09	0.25	-	0.57	18.60	0.57	1.00	4.99	1.00	0.44	0.96
	**GTR+I+G**	**012345**	**4109.55**	**58**	**5.04**	**0.29**	**0.36**	**0.09**	**0.26**	**-**	**0.70**	**13.02**	**0.24**	**0.41**	**3.70**	**1.00**	**0.44**	**1.00**
**ND4**	TIM3+I+G	012032	16946.70	56	0.00	0.29	0.38	0.10	0.24	-	0.65	10.55	1.00	0.65	4.91	1.00	0.34	0.68
	**GTR+I+G**	**012345**	**16944.87**	**58**	**0.33**	**0.29**	**0.38**	**0.09**	**0.24**	**-**	**0.48**	**9.77**	**0.81**	**0.74**	**4.12**	**1.00**	**0.34**	**0.66**
**ND4L**	TIM3+I+G	012032	3242.87	56	0.00	0.28	0.40	0.10	0.22	-	0.36	6.77	1.00	0.36	4.24	1.00	0.43	0.78
	**GTR+I+G**	**012345**	**3241.81**	**58**	**1.88**	**0.28**	**0.40**	**0.09**	**0.22**	**-**	**0.19**	**4.96**	**0.48**	**0.28**	**2.52**	**1.00**	**0.42**	**0.72**
**ND5**	TIM3+I+G	012032	23906.81	56	0.00	0.30	0.36	0.10	0.24	-	0.69	6.01	1.00	0.69	5.10	1.00	0.25	0.66
	**GTR+I+G**	**012345**	**23906.50**	**58**	**3.38**	**0.30**	**0.36**	**0.10**	**0.24**	**-**	**0.74**	**6.57**	**1.14**	**0.81**	**5.62**	**1.00**	**0.25**	**0.66**
**ND6**	**GTR+I+G**	**012345**	**6421.86**	**58**	**0.00**	**0.19**	**0.10**	**0.32**	**0.39**	**-**	**1.69**	**16.40**	**1.95**	**3.54**	**29.58**	**1.00**	**0.21**	**0.52**
**D-loop**	TPM3uf+G	012012	16372.77	54	0.00	0.33	0.23	0.13	0.31	-	0.81	2.35	1.00	0.81	2.35	1.00	-	0.98
	**GTR+G**	**012345**	**16370.70**	**58**	**1.87**	**0.33**	**0.23**	**0.14**	**0.30**	**-**	**0.97**	**2.44**	**1.20**	**0.83**	**2.85**	**1.00**	**-**	**0.99**
**CDS cc**	**GTR+I+G**	**012345**	**134175.66**	**58**	**0.00**	**0.29**	**0.35**	**0.11**	**0.25**	**-**	**0.56**	**5.45**	**0.80**	**0.40**	**4.37**	**1.00**	**0.37**	**0.71**
**CDS|rRNA cc**	TVM+I+G	012314	162809.38	57	0.00	0.31	0.34	0.12	0.24	-	0.83	5.59	1.08	0.42	5.59	1.00	0.38	0.70
	**GTR+I+G**	**012345**	**162809.01**	**58**	**1.26**	**0.31**	**0.34**	**0.12**	**0.24**	**-**	**0.84**	**5.51**	**1.10**	**0.42**	**5.72**	**1.00**	**0.38**	**0.70**
**CDS|rRNA|D-loop cc**	**GTR+I+G**	**012345**	**182119.44**	**58**	**0.00**	**0.31**	**0.32**	**0.12**	**0.25**	**-**	**0.84**	**4.53**	**1.10**	**0.40**	**4.81**	**1.00**	**0.36**	**0.83**
**CDS aa cc**	**MtMam+I+G+F**	**-**	**38935.34**	**68**	**0.00**	**-**	**-**	**-**	**-**	**-**	**-**	**-**	**-**	**-**	**-**	**-**	**0.26**	**0.41**

Table S6 Overview on bifurcation support values. Shown are all node support values obtained as described in Methods. Slashes (/) indicate a node being absent in a particular tree; numbers in brackets represent exchanges of species between two nodes (see Figure S2b); underlined values for nodes 22 and 23 imply that in this particular tree indeed two nodes were created while they typically (i.e. in most setups) collapsed (thus, the notation "22/23" in Figure 2); missing values are marked (-). Using mixed models in MrBayes gave exactly the same results as stated for the AIC-selected models (thus these results were omitted).

tool	data set	data type	support type	node 1	2	3	4	5	6	7	8	9	10	11	12	13	14	15	16	17	18	19	20	21	22	23	24
**PhyML**	#1	nuc up	SH-like	1	1	0.99	1	1	1	-	1	0.88	0.91	1	/	1	1	1	1	1	1	0.47	1	1	1	0.15	1
	#2	nuc up	SH-like	1	1	0.31 [11]	1	1	0.98	1	1	0.31	1	1 [3]	0.98	1	1	1	1	1	1	1	0.21	1	1	1	1
	#3	nuc up	SH-like	1	1	-	1	1	1	1	1	1	1	0.55	1	1	1	1	1	1	1	0.20	1	1	1	1	1
	#3 Gb	nuc up	SH-like	1	1	0.48	1	1	1	1	1	0.13	1	1	0.98	1	1	1	1	1	1	1	0.14	1	1	1	1
	#1 aa	aa up	SH-like	1	1	1	1	1	1	0.99	1	-	0	1	/	0.89	1	1	1	0.95	1	1	0.34	0.80	1	0.19	1
**RAxML**	#1	nuc p	rapid BS	100	100	9 [11]	100	100	100	-	100	90 [13]	84 [14]	99 [3]	/	100 [9]	100 [10]	100	100	100	100	64	100	100	100	62	100
	#2	nuc p	rapid BS	100	100	100	100	-	100	100	100	49	100	100	100	100	100	100	100	100	100	26	100	100	100	100	100
	#3	nuc p	rapid BS	100	100	100	100	100	100	100	100	99	99	100	100	100	99	100	99	99	100	46	98	98	92	92	92
	#3 Gb	nuc p	rapid BS	100	100	99	100	100	100	100	100	46	100	99	100	100	100	100	100	100	100	59	100	100	96	96	100
	#1 aa	aa p	rapid BS	100	100	100 [11]	-	100	100	88	100	83 [13]	37 [14]	100	/	93 [9]	100 [10]	100	100	100	100	44	67	100	61	100	100
**MrBayes**	#1	nuc p	4M gen	100	100	100	100	100	100	100	100	100 [13]	100 [14]	100	/	100 [9]	100 [10]	100	100	100	100	99	100	100	99	99	100
	#2	nuc p	4M gen	100	100	100	100	100	100	100	100	100	100	100	100	100	100	100	100	100	100	99	100	100	100	100	100
	#3	nuc p	4M gen	100	100	100	100	100	100	100	100	100	100	100	100	100	100	100	100	100	100	98	100	100	100	100	100
	#3 Gb	nuc p	4M gen	100	100	100	100	100	100	100	100	100	100	100	100	100	100	100	100	100	100	98	100	100	100	100	100
	#1 aa	aa p	4M gen	-	100	100 [11]	100	100	100	99	100	100	77 [14]	100 [3]	/	100	100 [10]	100	100	100	100	91	97	100	74	100	100

**#1**	CDS	**BS**	bootstrap	**nuc**	nucleotide
**#2**	CDS|rRNA	**SH-like**	Shimodaira-Hasegawa-like	**aa**	amino acid
**#3**	CDS|rRNA|D-loop	**4M gen**	4 million generations	**up**	unpartitioned
**#3 Gb**	CDS|rRNA|D-loop Gblocks-filtered			**p**	partitioned
**#1 aa**	CDS amino acid				

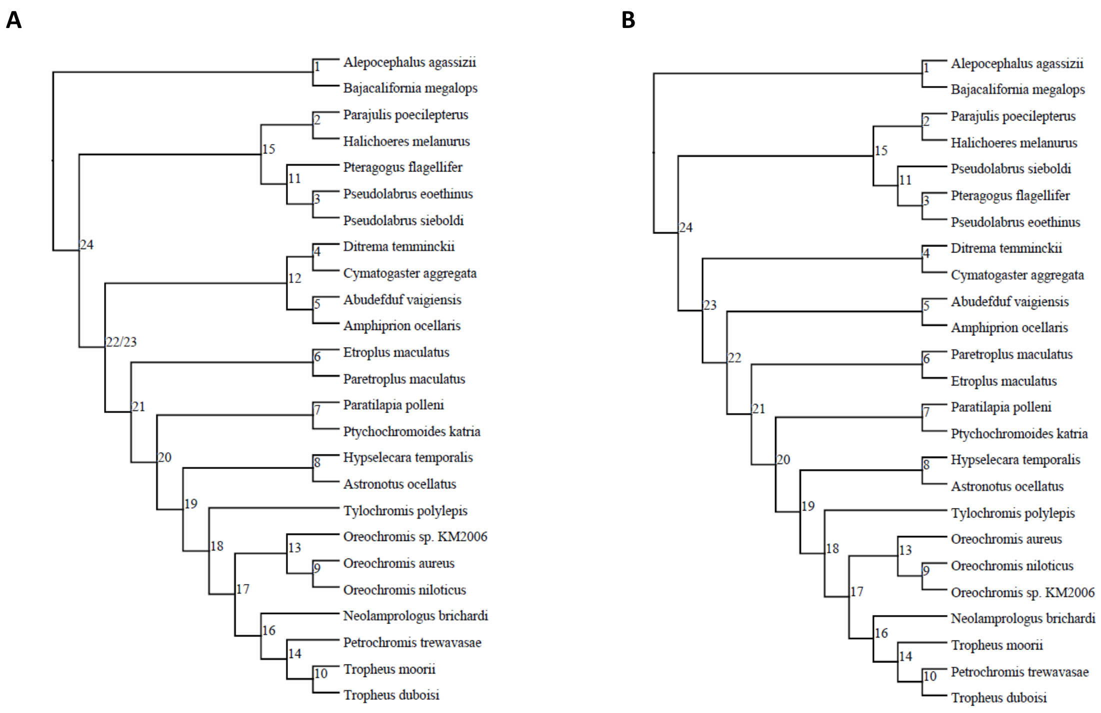

Figure S2 **Gene tree topologies.** Shown are both generated tree topologies. Topology **A** was obtained consistently for partitioned data sets #2 and #3 with different computational methods and was therefore presented as valid gene tree in **Figure 2**; topology **B** was obtained for data set #1 and includes all below mentioned inconsistencies of species-node assignments within tribes [3 ↔ 11; 9 ↔ 13; 10 ↔ 14]. Numbers at nodes relate to **Table S6**.

Using only coding sequences (data set #1) or amino acid sequences, there have been some inconsistencies: First, with data set #1 *P. trewavasae* was placed closest to *Tropheus duboisi* and *T. moorii* branched off earlier. Second, *Oreochromis aureus* and *O.* sp.-KM2006 switched places in the same way, where the order in #1 is consistent with the tree provided by He *et al.* [10], who also used only coding sequences in their analysis. Third, *Pseudolabrus sieboldi* and *Pteragogus flagellifer* switched places, putting *P. flagellifer* closer to *Pseudolabrus eoethinus* – this occurred only in the best ML tree and not in the majority rule consensus tree; it is very likely an artifact. In addition, the grouping of ((*Ditrema temminckii*, *Cymatogaster aggregata*)(*Amphiprion ocellaris*, *Abudefduf vaigiensis*)), i.e. the merging of the families Pomacentridae and Embiotocidae to sister groups, was not supported – but with weak support values for the split (see **Table S6**). These disagreements showed up similarly in the analyses based on amino acid sequences or on a codon position-wise partitioned data set #1 (**Figure S2**). In short, analysis solely based on coding sequences or amino acid sequences did not identify Pomacentridae and Embiotocidae as sister groups (with weak support for their split, though), and all other analyses did. Gblocks reduced data set #3 from 16,084 to 13,833 characters, where, as expected, mainly the D-loop region and rRNA sequences were affected from filtering (see **Table S3A**). Full and Gblocks-filtered alignments still resulted in the same topology, both under ML and BI.

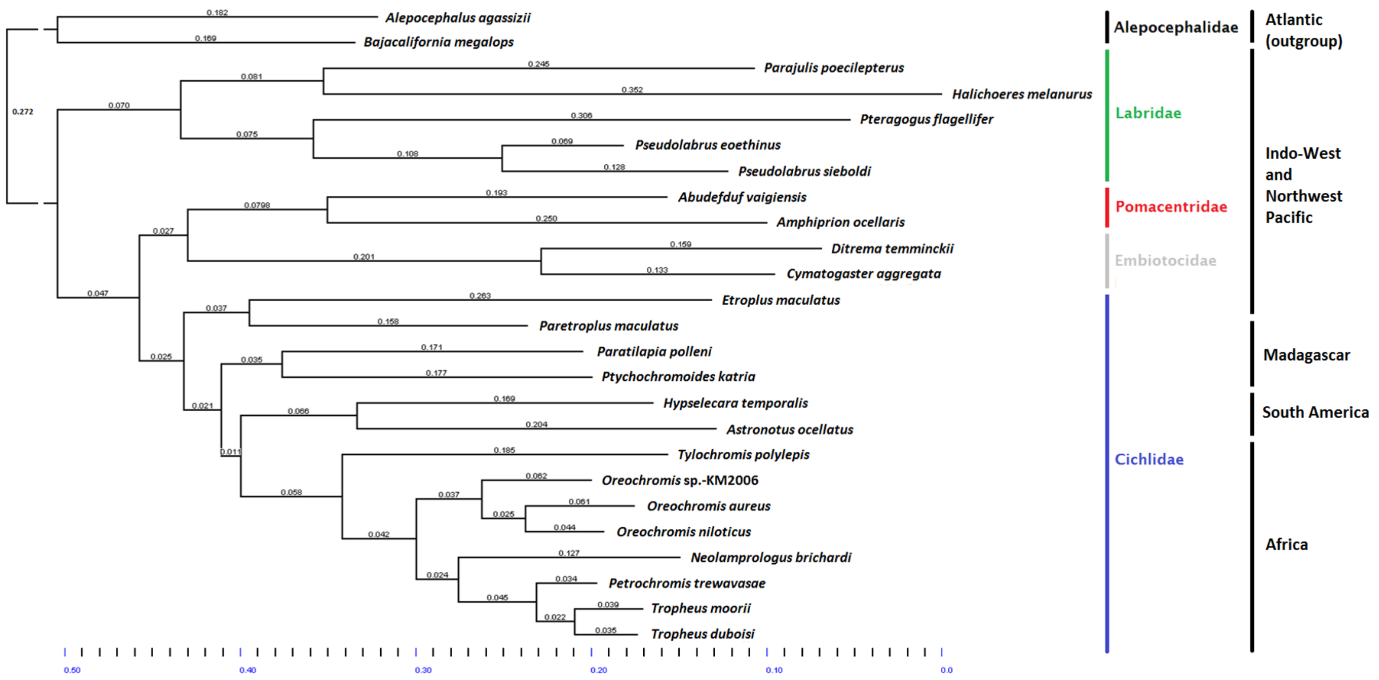

Figure S3 Phylogenetic relationships among labroid families analyzed in this study. Shown is a representative phylogram with branch lengths (expected number of substitutions per site) calculated by maximum likelihood (RAxML) on the gene wise partitioned data set #3 (i.e., all sequences except those of tRNA genes).

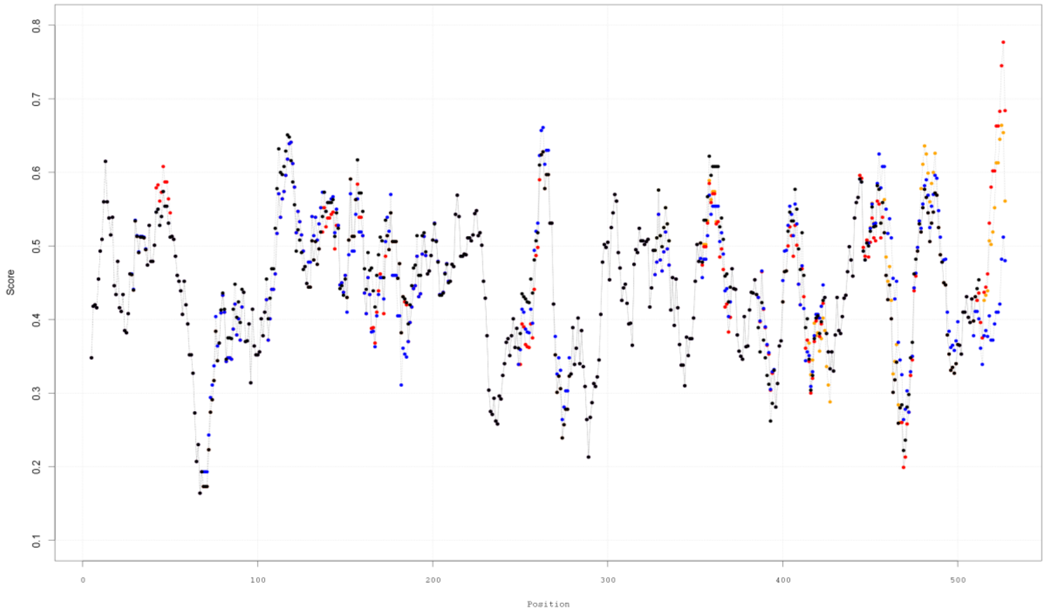

Figure S4 Amino acid accessibility of *COX-1* genes. Shown are scale profiles (% accessible) for *Tropheus duboisi* (orange), *Oreochromis aureus* (red), *Cymatogaster aggregata* (blue), and *Alepocephalus agassizii* (black); scores are normalized (0,1) with a higher score indicating higher accessibility. High accessibility of the gained C-terminal sequences is obvious (orange and red peak on the right).

Table S7 Information criterion-based selection of nucleotide substitution models (without outgroup). Best nucleotide substitution models were determined out of 88 candidate models (11 substitution schemes, G, I and F) with 8 rate categories for the gamma distribution; a maximum likelihood tree was used as base tree for likelihood calculations, best of NNI and SPR as tree topology search option. Eventually selected models for calculations are highlighted (bold); based on the results from Posada and Buckley [67], AIC/AICc or BIC were used for model selection. For comparison purposes models were rated according to AIC, BIC, DT and AICc (except for 4-fold degenerate sites); sequence length was used to approximate sample size in AICc, BIC, and DT calculations. ALT indicates the manual selection of an alternative model, performed when information criteria have chosen models with obvious misoperation of the model fitting or likelihood approach (with extremely high estimates of relative substitution rates). Abbreviations: 4fd, fourfold degenerate; cp, coding position; AIC, Akaike information criterion; AICc, AIC corrected for small sample size; BIC, Bayesian information criterion; DT, decision-theoretic performance-based approach;

Name	Criterion	Model	Partition	-lnL	Parameters	f(A)	f(C)	f(G)	f(T)	kappa	titv	rAC	rAG	rAT	rCG	rCT	rGT	p-inv	alpha
**12S rRNA**	**all**	**TIM2+I+G**	**010232**	**6631.09**	**52**	**0.33**	**0.24**	**0.20**	**0.23**	**0.00**	**0.00**	**6.54**	**15.50**	**6.54**	**1.00**	**40.61**	**1.00**	**0.36**	**0.50**
**16S rRNA**	**all**	**TIM2+I+G**	**010232**	**14239.55**	**52**	**0.36**	**0.26**	**0.18**	**0.21**	**0.00**	**0.00**	**6.92**	**12.61**	**6.92**	**1.00**	**37.62**	**1.00**	**0.27**	**0.54**
**ATP6**	**AIC/AICc**	**TIM3+I+G**	**012032**	**7306.54**	**52**	**0.30**	**0.37**	**0.09**	**0.24**	**0.00**	**0.00**	**0.59**	**11.09**	**1.00**	**0.59**	**6.53**	**1.00**	**0.39**	**0.56**
	BIC/DT	TrN+I+G	010020	7308.81	51	0.30	0.36	0.09	0.25	0.00	0.00	1.00	14.92	1.00	1.00	8.70	1.00	0.39	0.54
**ATP6_cp1**	AIC/AICc	TIM2+G	010232	1551.16	51	0.26	0.37	0.21	0.16	0.00	0.00	3.28	13.98	3.28	1.00	51.79	1.00	-	0.24
	**BIC/DT**	**TrN+G**	**010020**	**1553.37**	**50**	**0.29**	**0.36**	**0.19**	**0.16**	**0.00**	**0.00**	**1.00**	**6.77**	**1.00**	**1.00**	**22.99**	**1.00**	**-**	**0.24**
**ATP6_cp2**	AIC/AICc	TVM+G	012314	655.24	52	0.15	0.28	0.11	0.47	0.00	0.00	7.63E+02	1.39E+03	7.65E+02	2.06E+03	1.39E+03	1.00	-	0.10
	**BIC/DT**	**F81+G**	**000000**	**663.95**	**48**	**0.14**	**0.30**	**0.11**	**0.46**	**0.00**	**0.00**	**1.00**	**1.00**	**1.00**	**1.00**	**1.00**	**1.00**	**-**	**0.10**
**ATP6_cp3**	**all**	**TIM3+I+G**	**012032**	**4653.00**	**52**	**0.33**	**0.36**	**0.08**	**0.23**	**0.00**	**0.00**	**0.35**	**18.20**	**1.00**	**0.35**	**6.68**	**1.00**	**0.01**	**1.42**
**ATP6_4fd**	AIC	TPM1uf+G	012210	1710.58	50	0.35	0.40	0.06	0.19	0.00	0.00	1.00	11.63	3.15	3.15	11.63	1.00	-	5.78
	**BIC/DT**	**HKY+G**	**010010**	**1711.81**	**49**	**0.35**	**0.40**	**0.06**	**0.19**	**6.43**	**2.59**	**1.00**	**6.43**	**1.00**	**1.00**	**6.43**	**1.00**	**-**	**4.67**
**ATP8**	**all**	**TrN+G**	**010020**	**1680.45**	**50**	**0.29**	**0.35**	**0.09**	**0.28**	**0.00**	**0.00**	**1.00**	**10.99**	**1.00**	**1.00**	**8.90**	**1.00**	**-**	**0.29**
**ATP8_cp1**	**AIC**	**TPM3uf+G**	**012012**	**432.32**	**50**	**0.31**	**0.29**	**0.17**	**0.24**	**0.00**	**0.00**	**0.23**	**2.56**	**1.00**	**0.23**	**2.56**	**1.00**	**-**	**0.40**
	AICc	JC	000000	481.14	44	0.25	0.25	0.25	0.25	0.00	0.00	1.00	1.00	1.00	1.00	1.00	1.00	-	-
	BIC/DT	TPM3+G	012012	435.60	47	0.25	0.25	0.25	0.25	0.00	0.00	0.30	2.61	1.00	0.30	2.61	1.00	-	0.40
**ATP8_cp2**	**AIC**	**TPM3uf+G**	**012012**	**233.01**	**50**	**0.22**	**0.36**	**0.11**	**0.31**	**0.00**	**0.00**	**4.56**	**5.63**	**1.00**	**4.56**	**5.63**	**1.00**	**-**	**0.42**
	AICc	JC	000000	250.82	44	0.25	0.25	0.25	0.25	0.00	0.00	1.00	1.00	1.00	1.00	1.00	1.00	-	-
	BIC/DT	F81+G	000000	235.03	48	0.21	0.40	0.11	0.28	0.00	0.00	1.00	1.00	1.00	1.00	1.00	1.00	-	0.44
**ATP8_cp3**	**AIC/BIC/DT**	**TPM2uf+G**	**010212**	**874.43**	**50**	**0.31**	**0.34**	**0.06**	**0.28**	**0.00**	**0.00**	**0.31**	**5.61**	**0.31**	**1.00**	**5.61**	**1.00**	**-**	**1.07**
	AICc	TrNef	010020	908.37	46	0.25	0.25	0.25	0.25	0.00	0.00	1.00	1.67	1.00	1.00	10.65	1.00	-	-
**ATP8_4fd**	AIC	TVM+G	012314	177.74	52	0.35	0.40	0.03	0.22	0.00	0.00	1.00	5.26E+05	1.58E+05	2.71E+06	5.26E+05	1.00	-	2196.32
	BIC/DT	TPM1uf+G	012210	179.81	50	0.37	0.38	0.05	0.20	0.00	0.00	1.00	1.68E+06	6.67E+05	6.67E+05	1.68E+06	1.00	-	22.77
	**ALT**	**TIM2**	**010232**	**180.84**	**50**	**0.36**	**0.37**	**0.03**	**0.24**	**0.00**	**0.00**	**1.00E-04**	**0.69**	**1.00E-04**	**1.00**	**0.12**	**1.00**	**-**	**-**
**CYTB**	**AIC/AICc**	**TIM3+I+G**	**012032**	**11406.98**	**52**	**0.29**	**0.38**	**0.09**	**0.24**	**0.00**	**0.00**	**0.56**	**6.17**	**1.00**	**0.56**	**8.98**	**1.00**	**0.40**	**0.51**
	BIC/DT	HKY+I+G	010010	11413.03	50	0.28	0.38	0.09	0.25	10.46	5.43	1.00	10.46	1.00	1.00	10.46	1.00	0.40	0.51
**CYTB_cp1**	**AIC**	**TIM3+I+G**	**012032**	**2206.58**	**52**	**0.24**	**0.30**	**0.25**	**0.21**	**0.00**	**0.00**	**0.40**	**4.77**	**1.00**	**0.40**	**7.67**	**1.00**	**0.50**	**0.67**
	AICc	TPM3uf+G	012012	2209.04	50	0.22	0.31	0.24	0.22	0.00	0.00	0.43	6.26	1.00	0.43	6.26	1.00	-	0.19
	BIC/DT	K80+G	010010	2215.10	46	0.25	0.25	0.25	0.25	9.08	4.54	1.00	9.08	1.00	1.00	9.08	1.00	-	0.21
**CYTB_cp2**	**AIC/AICc**	**TPM3uf+I+G**	**012012**	**1013.67**	**51**	**0.19**	**0.27**	**0.14**	**0.40**	**0.00**	**0.00**	**7.08**	**15.78**	**1.00**	**7.08**	**15.78**	**1.00**	**0.46**	**0.40**
	BIC/DT	TPM3uf+G	012012	1015.55	50	0.19	0.27	0.14	0.40	0.00	0.00	6.92	15.13	1.00	6.92	15.13	1.00	-	0.16
**CYTB_cp3**	**all**	**TIM1+I+G**	**012230**	**7396.72**	**52**	**0.29**	**0.43**	**0.05**	**0.24**	**0.00**	**0.00**	**1.00**	**135.65**	**6.60**	**6.60**	**45.15**	**1.00**	**0.01**	**1.39**
**CYTB_4fd**	AIC	TPM1uf+I+G	012210	2863.20	51	0.34	0.41	0.05	0.21	0.00	0.00	1.00	11.26	3.27	3.27	11.26	1.00	0.01	6.51
	**BIC/DT**	**TPM1uf+G**	**012210**	**2865.35**	**50**	**0.34**	**0.41**	**0.05**	**0.20**	**0.00**	**0.00**	**1.00**	**11.23**	**3.36**	**3.36**	**11.23**	**1.00**	**-**	**5.50**
**COX1**	**AIC/AICc**	**TVM+I+G**	**012314**	**14491.75**	**53**	**0.29**	**0.31**	**0.12**	**0.28**	**0.00**	**0.00**	**0.98**	**9.12**	**1.59**	**0.46**	**9.12**	**1.00**	**0.53**	**0.98**
	BIC/DT	TPM3uf+I+G	012012	14497.71	51	0.30	0.31	0.12	0.28	0.00	0.00	0.57	6.84	1.00	0.57	6.84	1.00	0.53	0.92
**COX1_cp1**	**AIC**	**GTR+I+G**	**012345**	**2213.84**	**54**	**0.25**	**0.25**	**0.29**	**0.22**	**0.00**	**0.00**	**0.40**	**2.28**	**0.78**	**0.08**	**6.83**	**1.00**	**0.58**	**0.60**
	AICc	SYM+I+G	012345	2217.20	51	0.25	0.25	0.25	0.25	0.00	0.00	0.41	2.52	0.68	0.10	6.07	1.00	0.57	0.57
	BIC/DT	TIM3ef+I+G	012032	2220.33	49	0.25	0.25	0.25	0.25	0.00	0.00	0.28	2.93	1.00	0.28	7.06	1.00	0.57	0.57
**COX1_cp2**	**all**	**TPM3uf+I+G**	**012012**	**1101.81**	**51**	**0.18**	**0.27**	**0.16**	**0.40**	**0.00**	**0.00**	**10.09**	**4.77**	**1.00**	**10.09**	**4.77**	**1.00**	**0.69**	**0.17**
**COX1_cp3**	AIC/AICc	GTR+G	012345	10174.37	53	0.30	0.33	0.08	0.29	0.00	0.00	0.90	20.86	1.58	0.24	9.60	1.00	-	1.50
	**BIC/DT**	**TIM3+G**	**012032**	**10177.03**	**51**	**0.30**	**0.33**	**0.07**	**0.29**	**0.00**	**0.00**	**0.53**	**15.26**	**1.00**	**0.53**	**6.66**	**1.00**	**-**	**1.50**
**COX1_4fd**	**AIC/BIC/DT**	**TrN+I+G**	**010020**	**4963.83**	**51**	**0.33**	**0.33**	**0.07**	**0.27**	**0.00**	**0.00**	**1.00**	**6.98**	**1.00**	**1.00**	**3.24**	**1.00**	**0.02**	**5.75**
**COX2**	**AIC/AICc**	**TVM+I+G**	**012314**	**6001.92**	**53**	**0.32**	**0.31**	**0.11**	**0.26**	**0.00**	**0.00**	**4.56**	**81.97**	**12.75**	**6.96**	**81.97**	**1.00**	**0.37**	**0.39**
	BIC/DT	TPM1uf+I+G	012210	6004.79	51	0.33	0.31	0.10	0.26	0.00	0.00	1.00	21.82	2.68	2.68	21.82	1.00	0.36	0.37
**COX2_cp1**	AIC	TIM2+G	010232	1033.52	51	0.23	0.27	0.30	0.19	0.00	0.00	7.44	33.00	7.44	1.00	60.57	1.00	-	0.20
	**AICc/BIC/DT**	**TPM2+G**	**010212**	**1037.75**	**47**	**0.25**	**0.25**	**0.25**	**0.25**	**0.00**	**0.00**	**4.91**	**28.35**	**4.91**	**1.00**	**28.35**	**1.00**	**-**	**0.23**

Name	Criterion	Model	Partition	-lnL	Parameters	f(A)	f(C)	f(G)	f(T)	kappa	titv	rAC	rAG	rAT	rCG	rCT	rGT	p-inv	alpha
COX2_cp2	AIC	TVM+G	012314	481.71	52	0.26	0.24	0.12	0.38	0.00	0.00	3.99E+03	7.42E+03	4.43E+03	1.96E+04	7.42E+03	1.00	-	0.14
	AICc/BIC/DT	F81+G	000000	488.03	48	0.26	0.25	0.12	0.37	0.00	0.00	1.00	1.00	1.00	1.00	1.00	1.00	-	0.14
COX2_cp3	AIC	TIM3+I+G	012032	4013.80	52	0.33	0.34	0.06	0.27	0.00	0.00	0.39	28.05	1.00	0.39	7.98	1.00	0.02	1.12
	AICc/BIC/DT	TIM3+G	012032	4015.41	51	0.33	0.34	0.06	0.27	0.00	0.00	0.39	22.60	1.00	0.39	8.49	1.00	-	0.97
COX2_cp4fd	AIC/BIC/DT	TrN+G	010020	2483.26	50	0.29	0.47	0.05	0.19	0.00	0.00	1.00	12.89	1.00	1.00	3.68	1.00	-	3.89
COX3	all	TPM3uf+I+G	012012	7118.53	51	0.31	0.35	0.10	0.24	0.00	0.00	0.48	6.15	1.00	0.48	6.15	1.00	0.50	0.71
COX3_cp1	AIC	TVMef+I	012314	1234.07	49	0.25	0.25	0.25	0.25	0.00	0.00	0.72	3.16	0.71	0.21	3.16	1.00	0.71	-
	AICc	TPM3+I	012012	1236.44	47	0.25	0.25	0.25	0.25	0.00	0.00	0.54	3.70	1.00	0.54	3.70	1.00	0.71	-
	BIC/DT	K80+I	010010	1239.04	46	0.25	0.25	0.25	0.25	5.46	2.73	1.00	5.47	1.00	1.00	5.47	1.00	0.71	-
COX3_cp2	AIC	GTR+G	012345	614.47	53	0.21	0.26	0.16	0.37	0.00	0.00	8.66E+05	3.59E+05	2.02E+06	1.39E+06	3.33E+06	1.00	-	0.11
	AICc/DT	TIM1+G	012230	617.09	51	0.22	0.26	0.16	0.37	0.00	0.00	1.00	0.96	4.54	4.54	8.46	1.00	-	0.11
	BIC	TPM1uf+G	012210	619.86	50	0.21	0.27	0.15	0.38	0.00	0.00	1.00	7.77	5.81	5.81	7.77	1.00	-	0.10
COX3_cp3	all	TIM3+I+G	012032	4746.16	52	0.32	0.39	0.06	0.24	0.00	0.00	0.39	35.74	1.00	0.39	8.49	1.00	0.03	1.11
COX3_cp4fd	AIC	TrN+I+G	010020	2168.16	51	0.33	0.39	0.05	0.22	0.00	0.00	1.00	11.31	1.00	1.00	3.72	1.00	0.04	7.63
	BIC/DT	TrN+I	010020	2170.33	50	0.35	0.39	0.05	0.21	0.00	0.00	1.00	14.41	1.00	1.00	3.70	1.00	0.04	-
D-loop	AIC/AICc	TIM2+G	010232	14383.32	51	0.34	0.22	0.14	0.31	0.00	0.00	1.23	2.75	1.23	1.00	3.31	1.00	-	0.87
	BIC/DT	HKY+G	010010	14386.32	49	0.34	0.22	0.13	0.32	2.67	1.20	1.00	2.67	1.00	1.00	2.67	1.00	-	0.87
D-loop Gb	AIC/AICc	TPM2uf+G	010212	10539.09	50	0.32	0.22	0.13	0.33	0.00	0.00	1.24	2.93	1.24	1.00	2.93	1.00	-	0.85
	BIC/DT	HKY+G	010010	10541.49	49	0.33	0.22	0.12	0.33	2.54	1.16	1.00	2.55	1.00	1.00	2.55	1.00	-	0.84
ND1	all	TrN+I+G	010020	10136.87	51	0.30	0.36	0.09	0.26	0.00	0.00	1.00	14.88	1.00	1.00	7.25	1.00	0.41	0.63
ND1_cp1	AIC	GTR+I+G	012345	2051.50	54	0.24	0.32	0.26	0.19	0.00	0.00	0.97	4.94	1.80	0.13	11.85	1.00	0.50	1.31
	AICc	TIM2ef+I+G	010232	2058.28	49	0.25	0.25	0.25	0.25	0.00	0.00	2.92	12.04	2.92	1.00	22.12	1.00	0.45	1.01
	BIC/DT	TIM2ef+G	010232	2060.46	48	0.25	0.25	0.25	0.25	0.00	0.00	3.21	12.66	3.21	1.00	23.37	1.00	-	0.29
ND1_cp2	AIC/AICc	TIM1+G	012230	803.12	51	0.17	0.29	0.11	0.42	0.00	0.00	1.00	5.79	6.28	6.28	32.83	1.00	-	0.08
	BIC/DT	TrN+G	010020	804.75	50	0.17	0.29	0.11	0.42	0.00	0.00	1.00	1.55	1.00	1.00	8.76	1.00	-	0.08
ND1_cp3	AIC/AICc	GTR+I+G	012345	6612.09	54	0.33	0.36	0.07	0.24	0.00	0.00	5.47E+03	3.83E+05	8.89E+03	3.80E+04	7.33E+04	1.00	0.00	1.72
	BIC/DT	TrN+I+G	010020	6617.85	51	0.33	0.37	0.07	0.24	0.00	0.00	1.00	38.89	1.00	1.00	7.67	1.00	0.00	1.79
ND1_4fd	AIC	TIM1+G	012230	2573.63	51	0.33	0.43	0.06	0.18	0.00	0.00	1.00	12.38	1.75	1.75	3.69	1.00	-	9.10
	BIC/DT	TrN+G	010020	2574.68	50	0.33	0.42	0.06	0.19	0.00	0.00	1.00	9.05	1.00	1.00	2.88	1.00	-	8.48
ND2	AIC/AICc	GTR+I+G	012345	12051.51	54	0.31	0.39	0.08	0.23	0.00	0.00	0.26	4.33	0.43	0.33	2.02	1.00	0.31	0.67
	BIC/DT	TIM3+I+G	012032	12056.13	52	0.31	0.38	0.09	0.22	0.00	0.00	0.50	6.56	1.00	0.50	3.75	1.00	0.31	0.68
ND2_cp1	AIC/AICc	GTR+I+G	012345	2869.45	54	0.30	0.33	0.20	0.17	0.00	0.00	0.27	1.61	1.08	0.00	2.62	1.00	0.39	2.13
	BIC/DT	TVM+I+G	012314	2872.20	53	0.29	0.34	0.18	0.19	0.00	0.00	0.31	2.14	1.06	0.00	2.14	1.00	0.39	1.94
ND2_cp2	AIC/AICc	TVM+G	012314	1361.76	52	0.15	0.35	0.11	0.38	0.00	0.00	5.45	20.69	4.76	11.93	20.69	1.00	-	0.22
	BIC/DT	HKY+G	010010	1367.04	49	0.15	0.37	0.12	0.37	3.72	2.89	1.00	3.72	1.00	1.00	3.72	1.00	-	0.21
ND2_cp3	all	TIM2+I+G	010232	7181.06	52	0.35	0.38	0.06	0.21	0.00	0.00	0.16	12.09	0.16	1.00	2.46	1.00	0.01	1.74
ND2_4fd	AIC/BIC/DT	TrN+G	010020	2483.26	50	0.29	0.47	0.05	0.19	0.00	0.00	1.00	12.89	1.00	1.00	3.68	1.00	-	3.89
ND3	all	TIM1+I+G	012230	3754.25	52	0.28	0.35	0.09	0.28	0.00	0.00	1.00	15.16	0.19	0.19	4.18	1.00	0.42	0.84
ND3_cp1	AIC	TVMef+G	012314	794.89	49	0.25	0.25	0.25	0.25	0.00	0.00	1.55	5.66	1.42	0.01	5.66	1.00	-	0.33
	AICc/BIC/DT	TPM2+G	010212	797.11	47	0.25	0.25	0.25	0.25	0.00	0.00	3.78	14.06	3.78	1.00	14.06	1.00	-	0.33
ND3_cp2	AIC/BIC/DT	TPM2uf+G	010212	352.07	50	0.16	0.30	0.12	0.42	0.00	0.00	0.00	8.20	0.00	1.00	8.20	1.00	-	0.08
	AICc	HKY+G	010010	354.60	49	0.15	0.30	0.13	0.42	21.65	15.79	1.00	21.65	1.00	1.00	21.65	1.00	-	0.08
ND3_cp3	AIC	TIM3+G	012032	2344.95	51	0.32	0.35	0.08	0.25	0.00	0.00	5.30	110.76	1.00	5.30	37.10	1.00	-	1.47
	AICc/BIC/DT	TrN+G	010020	2346.76	50	0.32	0.36	0.08	0.24	0.00	0.00	1.00	33.56	1.00	1.00	11.55	1.00	-	1.44
ND3_4fd	AIC/BIC/DT	HKY+G	010010	805.52	49	0.27	0.48	0.06	0.19	6.15	3.05	1.00	6.15	1.00	1.00	6.15	1.00	-	2.45
ND4	all	TIM3+I+G	012032	15501.98	52	0.29	0.37	0.09	0.25	0.00	0.00	0.69	10.54	1.00	0.69	4.95	1.00	0.34	0.67
ND4_cp1	AIC	TVM+I+G	012314	3605.72	53	0.29	0.32	0.19	0.20	0.00	0.00	0.44	3.81	1.26	0.19	3.81	1.00	0.42	1.19
	AICc/BIC/DT	TPM3uf+I+G	012012	3608.12	51	0.30	0.33	0.18	0.20	0.00	0.00	0.28	3.31	1.00	0.28	3.31	1.00	0.42	1.19
ND4_cp2	all	TVM+G	012314	1791.82	52	0.16	0.29	0.15	0.41	0.00	0.00	11.91	31.84	7.36	28.36	31.84	1.00	-	0.20
ND4_cp3	AIC/AICc	TIM3+I+G	012032	9283.04	52	0.32	0.38	0.07	0.23	0.00	0.00	0.59	26.12	1.00	0.59	7.11	1.00	0.01	1.53
	BIC/DT	TrN+I+G	010020	9285.58	51	0.32	0.38	0.07	0.23	0.00	0.00	1.00	35.08	1.00	1.00	9.47	1.00	0.01	1.52
ND4_4fd	AIC	TIM1+G	012230	3049.35	51	0.29	0.46	0.05	0.20	0.00	0.00	1.00	5.67	1.56	1.56	3.29	1.00	-	11.01
	BIC/DT	TrN+G	010020	3051.19	50	0.29	0.45	0.05	0.20	0.00	0.00	1.00	4.61	1.00	1.00	2.72	1.00	-	9.91

Name	Criterion	Model	Partition	-lnL	Parameters	f(A)	f(C)	f(G)	f(T)	kappa	titv	rAC	rAG	rAT	rCG	rCT	rGT	p-inv	alpha
ND4L	AIC	TIM3+I+G	012032	2946.15	52	0.28	0.39	0.10	0.23	0.00	0.00	0.29	6.81	1.00	0.29	4.38	1.00	0.47	0.94
	AICc/BIC/DT	TPM3uf+I+G	012012	2947.21	51	0.29	0.38	0.11	0.22	0.00	0.00	0.26	4.84	1.00	0.26	4.84	1.00	0.47	0.95
ND4L_cp1	AIC	TVM+I+G	012314	557.07	53	0.18	0.35	0.25	0.22	0.00	0.00	0.34	3.23	1.31	0.00	3.23	1.00	0.58	99.84
	AICc/BIC/DT	TPM3+I	012012	565.44	47	0.25	0.25	0.25	0.25	0.00	0.00	0.11	2.59	1.00	0.11	2.59	1.00	0.58	-
ND4L_cp2	AIC	TIM3+I	012032	225.52	51	0.12	0.33	0.16	0.39	0.00	0.00	5.18E+04	1.09E+06	1.00E+00	5.18E+04	1.26E+05	1.00	0.84	-
	AICc	K80+I	010010	238.16	46	0.25	0.25	0.25	0.25	12.81	6.40	1.00	12.81	1.00	1.00	12.81	1.00	0.86	-
	BIC/DT	TrN+I	010020	226.59	50	0.12	0.33	0.16	0.39	0.00	0.00	1.00	53.72	1.00	1.00	3.94	1.00	0.84	-
ND4L_cp3	all	TrN+I+G	010020	1900.36	51	0.31	0.39	0.06	0.24	0.00	0.00	1.00	30.30	1.00	1.00	8.09	1.00	0.04	3.15
ND4L_4fd	AIC	TIM2	010232	844.72	50	0.33	0.42	0.05	0.20	0.00	0.00	0.14	5.91	0.14	1.00	0.92	1.00	-	-
	BIC/DT	TrN	010020	846.23	49	0.32	0.43	0.05	0.20	0.00	0.00	1.00	25.63	1.00	1.00	4.02	1.00	-	-
ND5	AIC/AICc	TIM3+I+G	012032	22045.96	52	0.30	0.35	0.10	0.25	0.00	0.00	0.74	6.06	1.00	0.74	5.16	1.00	0.25	0.67
	BIC/DT	TPM3uf+I+G	012012	22047.17	51	0.31	0.34	0.11	0.24	0.00	0.00	0.72	5.45	1.00	0.72	5.45	1.00	0.24	0.67
ND5_cp1	AIC/AICc	GTR+I+G	012345	5546.70	54	0.30	0.26	0.23	0.21	0.00	0.00	1.25	3.13	1.19	0.28	4.08	1.00	0.28	0.84
	BIC/DT	TPM2+I+G	010212	5563.67	48	0.25	0.25	0.25	0.25	0.00	0.00	3.20	7.62	3.20	1.00	7.62	1.00	0.26	0.80
ND5_cp2	all	TVM+G	012314	3220.25	52	0.19	0.29	0.13	0.39	0.00	0.00	21.44	34.40	14.55	21.00	34.40	1.00	-	0.28
ND5_cp3	AIC/AICc	GTR+I+G	012345	12248.76	54	0.30	0.40	0.06	0.24	0.00	0.00	0.18	12.77	0.73	0.84	4.31	1.00	0.00	1.69
	BIC/DT	TIM1+I+G	012230	12251.86	52	0.30	0.40	0.06	0.24	0.00	0.00	1.00	62.42	4.64	4.64	22.91	1.00	0.00	1.75
ND5_4fd	AIC	TIM1+I+G	012230	3559.53	52	0.31	0.43	0.06	0.21	0.00	0.00	1.00	7.11	1.61	1.61	3.97	1.00	0.01	5.01
	BIC/DT	TrN+G	010020	3563.14	50	0.30	0.42	0.06	0.22	0.00	0.00	1.00	5.99	1.00	1.00	3.17	1.00	-	4.07
ND6	AIC/AICc	TIM3+I+G	012032	5888.56	52	0.19	0.10	0.32	0.38	0.00	0.00	1.96	12.12	1.00	1.96	21.37	1.00	0.20	0.47
	BIC/DT	HKY+I+G	010010	5892.05	50	0.19	0.12	0.31	0.39	11.60	4.74	1.00	11.60	1.00	1.00	11.60	1.00	0.23	0.55
ND6_cp1	all	TPM2uf+I+G	010212	1414.79	51	0.12	0.14	0.41	0.33	0.00	0.00	5.68	26.38	5.68	1.00	26.38	1.00	0.33	1.91
ND6_cp2	AIC	GTR+G	012345	816.49	53	0.12	0.18	0.23	0.46	0.00	0.00	0.10	1.94	1.01	3.47	6.70	1.00	-	0.26
	AICc	TIM3+G	012032	819.39	51	0.11	0.19	0.25	0.46	0.00	0.00	2.58	1.79	1.00	2.58	6.70	1.00	-	0.27
	BIC/DT	HKY+G	010010	824.06	49	0.09	0.22	0.23	0.45	2.77	1.55	1.00	2.77	1.00	1.00	2.77	1.00	-	0.28
ND6_cp3	all	TrN+G	010020	3292.54	50	0.24	0.07	0.34	0.36	0.00	0.00	1.00	15.73	1.00	1.00	95.48	1.00	-	1.27
ND6_4fd	AIC/BIC/DT	TIM1+G	012230	1211.98	51	0.20	0.08	0.32	0.40	0.00	0.00	1.00	25.37	7.26	7.26	193.58	1.00	-	2.97
tRNA-Ala-GCA	AIC	TPM3+I+G	012012	391.90	48	0.25	0.25	0.25	0.25	0.00	0.00	0.00	9.44	1.00	0.00	9.44	1.00	0.17	0.97
	AICc	K80	010010	400.85	45	0.25	0.25	0.25	0.25	15.74	7.87	1.00	15.74	1.00	1.00	15.74	1.00	-	-
	BIC	TPM3+G	012012	393.23	47	0.25	0.25	0.25	0.25	0.00	0.00	0.00	8.71	1.00	0.00	8.71	1.00	-	0.44
	DT	K80+G	010010	395.39	46	0.25	0.25	0.25	0.25	14.65	7.33	1.00	14.65	1.00	1.00	14.65	1.00	-	0.47
tRNA-Arg-CGA	AIC/BIC/DT	HKY+G	010010	483.50	49	0.32	0.23	0.14	0.32	9.24	4.32	1.00	9.24	1.00	1.00	9.24	1.00	-	0.56
	AICc	K80+G	010010	490.90	46	0.25	0.25	0.25	0.25	9.01	4.51	1.00	9.01	1.00	1.00	9.01	1.00	-	0.50
tRNA-Asn-AAC	AIC/BIC/DT	TPM3+I	012012	252.14	47	0.25	0.25	0.25	0.25	0.00	0.00	0.00	6.11	1.00	0.00	6.11	1.00	0.67	-
	AICc	K80+I	010010	254.99	46	0.25	0.25	0.25	0.25	11.31	5.65	1.00	11.31	1.00	1.00	11.31	1.00	0.67	-
tRNA-Asp-GAC	AIC	TPM3uf+G	012012	585.88	50	0.35	0.20	0.18	0.26	0.00	0.00	0.15	8.88	1.00	0.15	8.88	1.00	-	0.35
	AICc	K80+G	010010	595.01	46	0.25	0.25	0.25	0.25	11.70	5.85	1.00	11.70	1.00	1.00	11.70	1.00	-	0.36
	BIC/DT	TPM2+G	010212	591.30	47	0.25	0.25	0.25	0.25	0.00	0.00	1.78E+04	1.05E+05	1.78E+04	1.00	1.05E+05	1.00	-	0.38
tRNA-Cys-UGC	AIC	TPM2uf+G	010212	544.80	50	0.17	0.23	0.27	0.34	0.00	0.00	10.13	32.10	10.13	1.00	32.10	1.00	-	0.35
	AICc	K80+G	010010	553.19	46	0.25	0.25	0.25	0.25	6.89	3.45	1.00	6.89	1.00	1.00	6.89	1.00	-	0.36
	BIC/DT	TPM2+G	010212	549.57	47	0.25	0.25	0.25	0.25	0.00	0.00	4.84	19.72	4.84	1.00	19.72	1.00	-	0.36
tRNA-Gln-CAA	AIC/BIC/DT	TIM2ef+G	010232	251.83	48	0.25	0.25	0.25	0.25	0.00	0.00	15.39	44.31	15.39	1.00	3.75	1.00	-	0.44
	AICc	K80	010010	263.17	45	0.25	0.25	0.25	0.25	4.28	2.14	1.00	4.28	1.00	1.00	4.28	1.00	-	-
tRNA-Glu-GAA	AIC	TPM3uf+G	012012	361.22	50	0.26	0.16	0.25	0.34	0.00	0.00	0.00	14.88	1.00	0.00	14.88	1.00	-	0.31
	AICc	K80+G	010010	369.21	46	0.25	0.25	0.25	0.25	20.95	10.48	1.00	20.95	1.00	1.00	20.95	1.00	-	0.30
	BIC/DT	TPM3+G	012012	364.90	47	0.25	0.25	0.25	0.25	0.00	0.00	0.00	10.68	1.00	0.00	10.68	1.00	-	0.31
tRNA-Gly-GGA	AIC	TPM3uf+G	012012	383.42	50	0.31	0.22	0.17	0.30	0.00	0.00	1.39E+06	3.84E+06	1.00	1.39E+06	3.84E+06	1.00	-	0.31
	AICc/BIC/DT	TPM3+G	012012	387.26	47	0.25	0.25	0.25	0.25	0.00	0.00	1.01E+06	2.86E+06	1.00	1.01E+06	2.86E+06	1.00	-	0.31
	ALT	TPM1uf+G	012210	389.84	50	0.32	0.25	0.16	0.27	0.00	0.00	1.00	3.62	0.08	0.08	3.62	1.00	-	0.29
tRNA-His-CAC	AIC	TPM2uf+G	010212	440.73	50	0.29	0.17	0.21	0.33	0.00	0.00	6.35E+03	4.33E+04	6.35E+03	1.00	4.33E+04	1.00	-	0.52
	AICc	K80+G	010010	448.58	46	0.25	0.25	0.25	0.25	10.93	5.47	1.00	10.93	1.00	1.00	10.93	1.00	-	0.48
	BIC/DT	TPM2+G	010212	445.04	47	0.25	0.25	0.25	0.25	0.00	0.00	6.67E+03	4.08E+04	6.67E+03	1.00	4.08E+04	1.00	-	0.46

Name	Criterion	Model	Partition	-lnL	Parameters	f(A)	f(C)	f(G)	f(T)	kappa	titv	rAC	rAG	rAT	rCG	rCT	rGT	p-inv	alpha
**tRNA-Ile-AUC**	AIC	SYM+G	012345	385.87	50	0.25	0.25	0.25	0.25	0.00	0.00	0.42	2.93	2.74	0.00	9.68	1.00	-	0.25
	AICc	K80+G	010010	395.63	46	0.25	0.25	0.25	0.25	6.95	3.48	1.00	6.95	1.00	1.00	6.95	1.00	-	0.20
	**BIC/DT**	**TIM3ef+G**	**012032**	**388.13**	**48**	**0.25**	**0.25**	**0.25**	**0.25**	**0.00**	**0.00**	**0.09**	**1.59**	**1.00**	**0.09**	**5.17**	**1.00**	**-**	**0.26**
**tRNA-Leu-CUA**	AIC	TrNef+I	010020	144.53	47	0.25	0.25	0.25	0.25	0.00	0.00	1.00	1.08E+06	1.00	1.00	5.11E+06	1.00	0.84	-
	AICc	K80	010010	150.14	45	0.25	0.25	0.25	0.25	6.48E+04	3.24E+04	1.00	6.48E+04	1.00	1.00	6.48E+04	1.00	-	-
	BIC/DT	K80+G	010010	145.82	46	0.25	0.25	0.25	0.25	9.60E+05	4.80E+05	1.00	9.60E+05	1.00	1.00	9.60E+05	1.00	-	0.05
	**ALT**	F81+G	000000	154.88	48	0.29	0.23	0.20	0.27	0.00	0.00	1.00	1.00	1.00	1.00	1.00	1.00	-	0.05
**tRNA-Leu-UUA**	all	TPM2+G	010212	446.18	47	0.25	0.25	0.25	0.25	0.00	0.00	1.11E+04	4.19E+04	1.11E+04	1.00	4.19E+04	1.00	-	0.39
	**ALT**	TrN+G	010020	449.94	50	0.36	0.22	0.18	0.24	0.00	0.00	1.00	6.56	1.00	1.00	10.20	1.00	-	0.38
**tRNA-Lys-AAA**	AIC	TIM2ef+G	010232	362.71	48	0.25	0.25	0.25	0.25	0.00	0.00	9.79	25.42	9.79	1.00	79.17	1.00	-	0.27
	AICc	K80+G	010010	368.88	46	0.25	0.25	0.25	0.25	8.22	4.11	1.00	8.22	1.00	1.00	8.22	1.00	-	0.27
	**BIC/DT**	**TPM2+G**	**010212**	**364.54**	**47**	**0.25**	**0.25**	**0.25**	**0.25**	**0.00**	**0.00**	**11.59**	**51.82**	**11.59**	**1.00**	**51.82**	**1.00**	**-**	**0.29**
**tRNA-Met-AUG**	AIC/BIC/DT	TPM2+G	010212	294.48	47	0.25	0.25	0.25	0.25	0.00	0.00	9.58E+05	1.48E+06	9.58E+05	1.00	1.48E+06	1.00	-	0.39
	**AICc**	**JC+I**	**000000**	**305.00**	**45**	**0.25**	**0.25**	**0.25**	**0.25**	**0.00**	**0.00**	**1.00**	**1.00**	**1.00**	**1.00**	**1.00**	**1.00**	**0.58**	**-**
**tRNA-Phe-UUC**	AIC	TrN+G	010020	340.60	50	0.36	0.22	0.22	0.20	0.00	0.00	1.00	6.20	1.00	1.00	14.53	1.00	-	0.53
	**AICc/BIC/DT**	**K80+G**	**010010**	**344.75**	**46**	**0.25**	**0.25**	**0.25**	**0.25**	**8.89**	**4.44**	**1.00**	**8.89**	**1.00**	**1.00**	**8.89**	**1.00**	**-**	**0.52**
**tRNA-Pro-CCA**	AIC	TPM1uf+I+G	012210	443.47	51	0.23	0.15	0.29	0.33	0.00	0.00	1.00	40.24	3.90	3.90	40.24	1.00	0.45	2.87
	**AICc/BIC/DT**	**K80+G**	**010010**	**451.05**	**46**	**0.25**	**0.25**	**0.25**	**0.25**	**15.92**	**7.96**	**1.00**	**15.92**	**1.00**	**1.00**	**15.92**	**1.00**	**-**	**0.42**
**tRNA-Ser-AGC**	AIC	TIM2ef+G	010232	617.79	48	0.25	0.25	0.25	0.25	0.00	0.00	4.97	19.66	4.97	1.00	31.03	1.00	-	0.81
	AICc	K80+G	010010	623.05	46	0.25	0.25	0.25	0.25	8.39	4.19	1.00	8.39	1.00	1.00	8.39	1.00	-	0.83
	**BIC/DT**	**TPM2+G**	**010212**	**618.82**	**47**	**0.25**	**0.25**	**0.25**	**0.25**	**0.00**	**0.00**	**5.84**	**28.46**	**5.84**	**1.00**	**28.46**	**1.00**	**-**	**0.82**
**tRNA-Ser-UCA**	**AIC/BIC/DT**	**TPM3+I**	**012012**	**290.08**	**47**	**0.25**	**0.25**	**0.25**	**0.25**	**0.00**	**0.00**	**0.00**	**8.66**	**1.00**	**0.00**	**8.66**	**1.00**	**0.73**	**-**
	AICc	K80+I	010010	293.01	46	0.25	0.25	0.25	0.25	14.45	7.23	1.00	14.45	1.00	1.00	14.45	1.00	0.73	-
**tRNA-Thr-ACA**	AIC	SYM+G	012345	463.15	50	0.25	0.25	0.25	0.25	0.00	0.00	4.79	6.88	0.03	0.00	18.14	1.00	-	0.25
	AICc	K80+G	010010	471.08	46	0.25	0.25	0.25	0.25	8.23	4.12	1.00	8.23	1.00	1.00	8.23	1.00	-	0.26
	**BIC**	**TIM1ef+G**	**012230**	**466.26**	**48**	**0.25**	**0.25**	**0.25**	**0.25**	**0.00**	**0.00**	**1.00**	**2.54**	**0.00**	**0.00**	**6.45**	**1.00**	**-**	**0.25**
	DT	TPM1+G	012210	468.61	47	0.25	0.25	0.25	0.25	0.00	0.00	1.00	4.17	0.01	0.01	4.17	1.00	-	0.26
**tRNA-Trp-UGA**	AIC	TIM2ef+G	010232	418.24	48	0.25	0.25	0.25	0.25	0.00	0.00	18.79	43.95	18.79	1.00	102.14	1.00	-	0.34
	**AICc**	**K80+G**	**010010**	**425.22**	**46**	**0.25**	**0.25**	**0.25**	**0.25**	**7.65**	**3.82**	**1.00**	**7.65**	**1.00**	**1.00**	**7.65**	**1.00**	**-**	**0.33**
	BIC/DT	TPM2+G	010212	419.51	47	0.25	0.25	0.25	0.25	0.00	0.00	99.96	336.48	99.96	1.00	336.48	1.00	-	0.31
**tRNA-Tyr-UAC**	**AIC/BIC/DT**	**TPM3+G**	**012012**	**442.47**	**47**	**0.25**	**0.25**	**0.25**	**0.25**	**0.00**	**0.00**	**0.07**	**7.89**	**1.00**	**0.07**	**7.89**	**1.00**	**-**	**0.20**
	AICc	K80+G	010010	444.70	46	0.25	0.25	0.25	0.25	13.29	6.64	1.00	13.29	1.00	1.00	13.29	1.00	-	0.20
**tRNA-Val-GUA**	AIC	TIM2ef+G	010232	417.87	48	0.25	0.25	0.25	0.25	0.00	0.00	9.60	22.24	9.60	1.00	41.09	1.00	-	0.79
	AICc	K80+G	010010	425.33	46	0.25	0.25	0.25	0.25	5.35	2.68	1.00	5.36	1.00	1.00	5.36	1.00	-	0.89
	**BIC/DT**	**TPM2+G**	**010212**	**419.16**	**47**	**0.25**	**0.25**	**0.25**	**0.25**	**0.00**	**0.00**	**10.15**	**31.04**	**10.15**	**1.00**	**31.04**	**1.00**	**-**	**0.86**

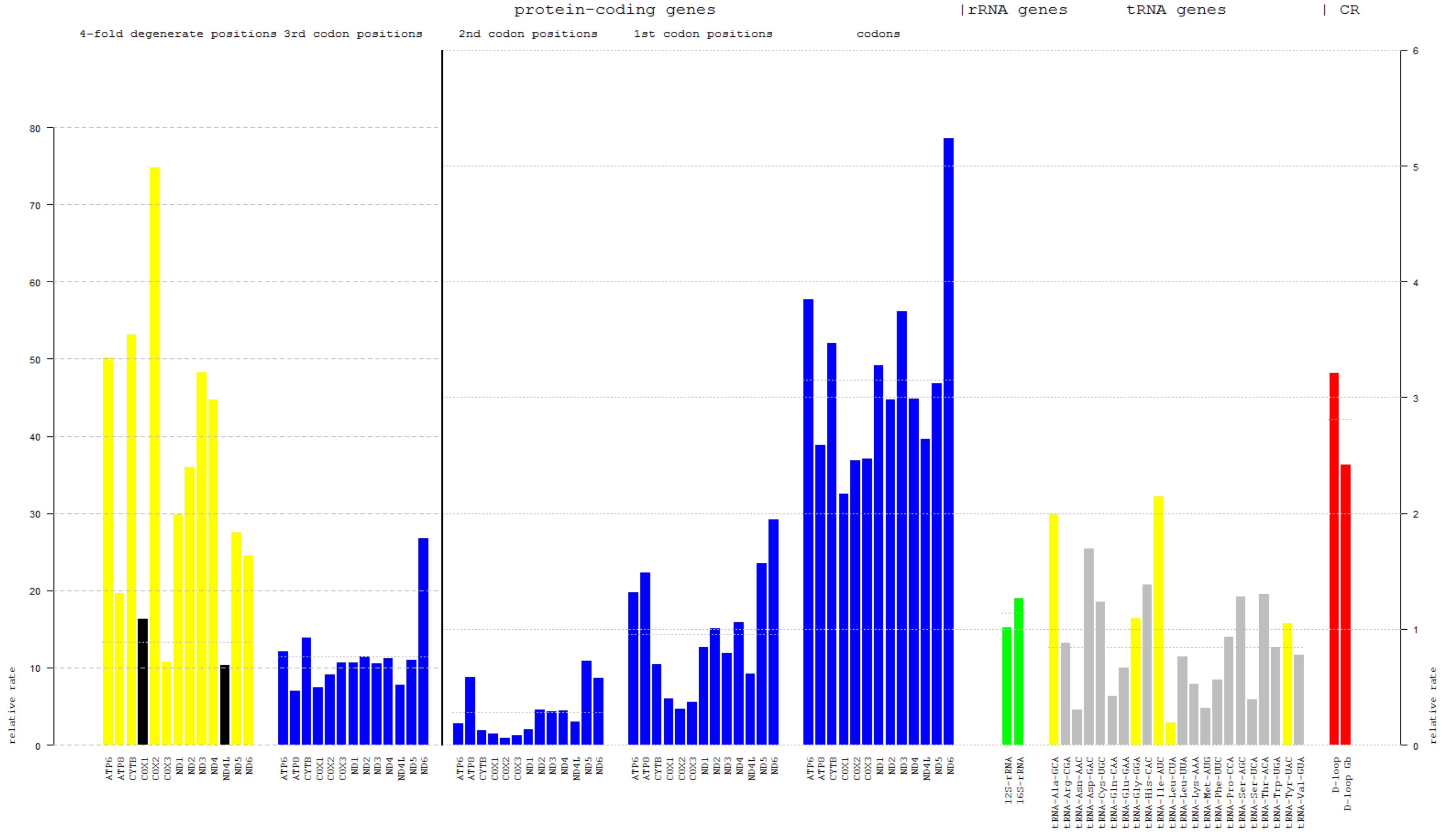

Figure S5 Relative rate of molecular evolution. Bars represent the mean rates estimated in a Bayesian MCMC analysis (BEAST 1.7.4), where *mean rate* is defined as the total number of substitutions per site divided by the total amount of time that the tree represents, or the mean of r_i (rate on i-th branch) weighted by t_i (the length of time of the i-th branch) (i.e., $\frac{\sum r_i \cdot t_i}{\sum t_i}$) (for individual bar heights see **Table S4b**). Group mean values are indicated **(**from left to right: **13.34**, **11.49**, **0.28**, **0.95**, **3.14**, **1.14**, 0.84). D-loop (**3.21**) represents the full non-coding region, whereas D-loop Gb (**2.42**) is based on a Gblocks-filtered multiple sequence alignment. Yellow bars refer to unreliable results as identified by the respective posterior distributions (skewed, long-tailed to the right; under the same gamma prior as used for all other genes or partitions).

Table S8 Molecular clock hypothesis tests. Molecular clock tests were performed for all genes using BEAST 1.7.4. Strict and lognormal relaxed clocks were tested on a tree model based on a Yule process tree prior; UPGMA starting trees were used. Two independent MCMC chains were set to 5 MIO steps being logged every 100th step. Parameter distributions were evaluated using Tracer 1.5. The clock-like behavior was validated by means of the standard deviation of the uncorrelated lognormal relaxed clock (UCLD stdev). If the UCLD stdev parameter estimate is close to 0.0 then the data is quite clock-like, if it is greater than 1.0 then the data exhibits substantial rate heterogeneity among lineages. ATP8, ND5, and some tRNAs show increased rate heterogeneity. The YBR is the rate of lineage birth in the Yule model of speciation. In the present setting this parameter estimates the number of lineages born from a parent lineage per substitution per site; all RNA genes exhibit relatively high birth rates. If the covariance (COV) between parent and child branch rates in a tree is significantly positive then branches with fast rates are followed by branches with fast rates; if the COV distribution spans zero (what is the case for all examined genes), then branches with fast rates and slow rates are next to each other (there is no strong evidence of autocorrelation of rates in the phylogenies). Abbreviations: COV, covariance; CV, coefficient of variation; RH, root height; YBR, Yule birthrate; shown are mean values only; all presented values have an effective sample size (ESS) > 200;

		lognormal relaxed molecular clock						strict molecular clock		
**Name**	**Model**	**UCLD stdev**	**COV**	**CV**	**RH**	**tree likelihood**	**YBR**	**RH**	**tree likelihood**	**YBR**
**12S rRNA**	GTR+I+G	**0.379**	-3.728 E-2	0.374	**0.272**	-6665.58	10.35	**0.259**	-6690.84	10.5
**16S rRNA**	GTR+I+G	**0.374**	2.935 E-2	0.360	**0.300**	-14272.62	7.87	**0.280**	-14314.02	8.12
**ATP6**	GTR+I+G	**0.332**	-7.439 E-3	0.330	**0.939**	-7333.67	2.61	**0.883**	-7348.09	2.66
**ATP8**	TN93+G	**0.802**	4.205 E-2	0.873	**0.587**	-1713.12	4.36	**0.590**	-1728.30	4.67
**CYTB**	GTR+I+G	**0.423**	-1.186 E-2	0.432	**0.767**	-11440.26	2.79	**0.820**	-11471.88	2.82
**COX1**	GTR+I+G	**0.243**	2.796 E-2	0.234	**0.452**	-14532.32	4.55	**0.429**	-14545.83	4.66
**COX2**	GTR+I+G	**0.185**	-2.928 E-3	0.181	**0.534**	-6040.78	4.15	**0.527**	-6044.91	4.15
**COX3**	TN93+I+G	**0.249**	-9.219 E-3	0.245	**0.533**	-7154.25	4.29	**0.523**	-7161.80	4.32
**ND1**	TN93+I+G	**0.325**	-5.436 E-2	0.316	**0.833**	-10163.76	2.86	**0.798**	-10181.73	2.96
**ND2**	GTR+I+G	**0.215**	-2.314 E-2	0.210	**0.651**	-12076.06	3.30	**0.630**	-12087.83	3.34
**ND3**	GTR+I+G	**0.308**	-1.402 E-2	0.303	**0.797**	-3787.24	2.78	**0.788**	-3795.26	2.76
**ND4**	GTR+I+G	**0.215**	-1.704 E-2	0.209	**0.856**	-15530.66	3.12	**0.855**	-15544.59	3.11
**ND4L**	TN93+I+G	**0.121**	-2.434 E-2	0.118	**0.605**	-2979.89	3.89	**0.606**	-2980.39	3.88
**ND5**	GTR+I+G	**0.836**	-9.101 E-2	0.867	**0.983**	-22084.53	2.93	**1.024**	-22255.75	3.18
**ND6**	GTR+I+G	**0.198**	-3.137 E-2	0.194	**1.375**	-5992.28	2.33	**1.420**	-5925.07	2.33
**D-loop**	GTR+G	**0.424**	-2.757 E-2	0.438	**0.678**	-14413.17	3.26	**0.723**	-14466.90	3.26
**D-loop Gb**	TN93+G	**0.430**	1.909 E-3	0.434	**0.505**	-10569.99	4.28	**0.540**	-10617.89	4.24
**tRNA-Ala**	TN93+G	**1.349**	-1.795 E-2	1.865	**0.345**	-423.83	11.69	**0.504**	-433.34	13.52
**tRNA-Arg**	HKY+G	**0.544**	1.332 E-2	0.581	**0.242**	-520.59	11.98	**0.263**	-524.33	11.94
**tRNA-Asn**	TN93+I	**0.296**	-2.210 E-2	0.300	**0.007**	-284.78	41.49	**0.007**	-285.85	42.11
**tRNA-Asp**	TN93+G	**0.401**	-2.316 E-2	0.410	**0.495**	-624.00	6.05	**0.516**	-626.02	6.23
**tRNA-Cys**	TN93+G	**0.241**	-1.095 E-2	0.240	**0.265**	-584.92	10.61	**0.258**	-586.06	10.94
**tRNA-Gln**	GTR+G	**0.668**	-1.212 E-2	0.726	**0.007**	-277.44	42.87	**0.007**	-281.02	42.31
**tRNA-Glu**	TN93+G	**0.327**	-1.144 E-2	0.332	**0.151**	-398.91	18.83	**0.147**	-400.33	19.30
**tRNA-Gly**	TN93+G	**0.922**	4.630 E-3	1.048	**0.219**	-428.16	13.40	**0.255**	-436.68	14.20
**tRNA-His**	TN93+G	**0.952**	8.444 E-3	1.099	**0.276**	-477.59	11.35	**0.312**	-485.00	11.91
**tRNA-Ile**	GTR+G	**0.313**	-7.436 E-3	0.317	**0.410**	-425.04	7.52	**0.351**	-424.21	8.73
**tRNA-Leu-CUA**	HKY+G	**0.265**	-2.211 E-2	0.267	**0.002**	-165.36	195.92	**0.002**	-165.63	193.00
**tRNA-Leu-UUA**	TN93+G	**0.541**	-1.002 E-2	0.560	**0.206**	-483.93	13.65	**0.195**	-488.77	14.17
**tRNA-Lys**	TN93+G	**0.265**	-2.323 E-2	0.266	**0.163**	-397.48	16.48	**0.157**	-398.61	16.93
**tRNA-Met**	TN93+G	**0.331**	5.452 E-4	0.339	**0.095**	-325.24	34.11	**0.094**	-326.14	34.85
**tRNA-Phe**	HKY+G	**0.251**	-1.730 E-2	0.251	**0.124**	-371.76	23.03	**0.124**	-372.52	23.09
**tRNA-Pro**	HKY+G	**0.588**	-7.612 E-3	0.623	**0.180**	-483.66	13.90	**0.182**	-490.28	14.37
**tRNA-Ser-AGC**	TN93+G	**0.392**	-7.983 E-3	0.398	**0.366**	-660.63	8.07	**0.348**	-665.04	8.43
**tRNA-Ser-UCA**	TN93+I	**0.423**	-1.876 E-2	0.438	**0.118**	-322.42	25.02	**0.117**	-323.92	25.40
**tRNA-Thr**	GTR+G	**0.397**	-3.751 E-3	0.412	**0.369**	-501.96	8.73	**0.362**	-502.22	9.11
**tRNA-Trp**	TN93+G	**0.828**	9.278 E-3	0.899	**0.212**	-456.95	13.67	**0.209**	-465.15	14.72
**tRNA-Tyr**	TN93+G	**0.275**	-1.297 E-2	0.277	**0.291**	-475.68	9.43	**0.288**	-476.68	9.49
**tRNA-Val**	TN93+G	**0.579**	-1.058 E-2	0.620	**0.171**	-457.33	18.27	**0.187**	-461.87	18.44

Table S9 Selection analyses of protein-coding genes. Several methods have been used to estimate the proportions of negatively or positively selected and neutrally evolving sites (see references in Methods). The REL (random effects likelihood) approach is an extension of the codon-based selection analyses pioneered by Nielsen and Yang, allowing synonymous rate variation; it is often the only method that can infer selection from small (≤ 15 sequences) or low divergence alignments, but is also susceptible to high rates of false positives. REL models variation in nonsynonymous and synonymous rates across sites according to a predefined distribution, with the selection pressure at an individual site inferred using an empirical Bayes approach. The FEL (fixed effects likelihood) method directly estimates nonsynonymous and synonymous substitution rates at each site; it is expected to give good site-by-site substitution rate estimates. Both methods incorporate flexible models of nucleotide substitution bias and variation in both nonsynonymous and synonymous substitution rates across sites. MEME (mixed effects model of evolution) combines fixed effects at the level of a site with random effects at the level of branches; this model is a generalization of FEL with the central extension that MEME models variable dN/dS ratios (ω) across lineages at an individual site whereas FEL applies the same ω to all branches. Unlike most other methods available, MEME can find signatures of episodic selection, even when the majority of lineages are subject to negative selection. Finally, FUBAR (fast unbiased approximate Bayesian) was applied on the data sets to also utilize a Bayesian MCMC-based approach, which is statistically more robust than REL. FEL and FUBAR results generally show very good agreement in the site-wise results (data not shown; nearly all FUBAR predictions of neutrally evolving sites are also predicted by FEL) but FEL stably estimates more codons as evolving neutrally; REL results do not correlate well. There is only a single codon (407 in *COX1*) which is jointly predicted (FEL, FUBAR and MEME) as being under positive selection. The numbers presented in the FUBAR columns may be regarded as estimation of the minimal proportions of neutrally evolving sites, allowing for stochastic substitution processes.

gene	substitution model	# sites total	substitutions/site REL | FEL | FUBAR	REL (>=50)			FEL (<=0.1)			FUBAR (>=0.9)			MEME (<=0.1)
				neg	neutral	pos	neg	neutral	pos	neg	neutral	pos	pos
**ATP6**	012213	227	1.91 | 1.10 | 3.30	223	1	3	209	18	0	**215**	**12**	**0**	codons: 65, 183
**ATP8**	010010	55	1.52 | 1.04 | 3.13	35	19	1	46	9	0	**45**	**10**	**0**	-
**CYTB**	012343	380	2.04 | 1.04 | 3.13	380	0	0	347	32	1	**364**	**16**	**0**	codon: 96
**COX1**	010232	531	1.55 | 0.88 | 2.63	499	30	2	485	45	1	**503**	**27**	**1**	codons: 407, 467, 484, 520, 527, 529
**COX2**	010232	230	1.64 | 0.82 | 2.46	230	0	0	208	22	0	**219**	**11**	**0**	codons: 144, 167
**COX3**	012343	261	1.57 | 0.84 | 2.52	213	47	1	236	25	0	**248**	**13**	**0**	-
**ND1**	012313	324	1.78 | 1.04 | 3.12	324	0	0	303	21	0	**313**	**11**	**0**	-
**ND2**	012313	349	1.88 | 1.19 | 3.58	297	51	1	318	31	0	**327**	**22**	**0**	codons: 237, 310, 321
**ND3**	012213	117	1.82 | 1.12 | 3.35	111	5	1	105	12	0	**107**	**10**	**0**	codon: 8
**ND4**	012313	460	1.89 | 1.14 | 3.43	399	58	3	410	50	0	**425**	**35**	**0**	codons: 18, 117, 132, 380, 391, 425
**ND4L**	010212	98	1.54 | 0.98 | 2.95	96	1	1	85	13	0	**89**	**9**	**0**	-
**ND5**	012314	621	2.06 | 1.24 | 3.73	556	65	0	516	105	0	**562**	**59**	**0**	codons: 218,279,292,566,577,584,606,611,621
**ND6**	012030	176	2.37 | 1.26 | 3.79	176	0	0	144	32	0	**149**	**27**	**0**	codons: 100, 103

no rates with dN>dS were inferred for this data sets, suggesting that all sites are under purifying selection

no sites with evidence of episodic diversifying selection

Significance levels

Bayes Factor	50	(REL)
Posterior probability	0.9	(FUBAR)
p-value	0.1	(FEL, MEME)

ATP synthase F0 subunit 6

(a)

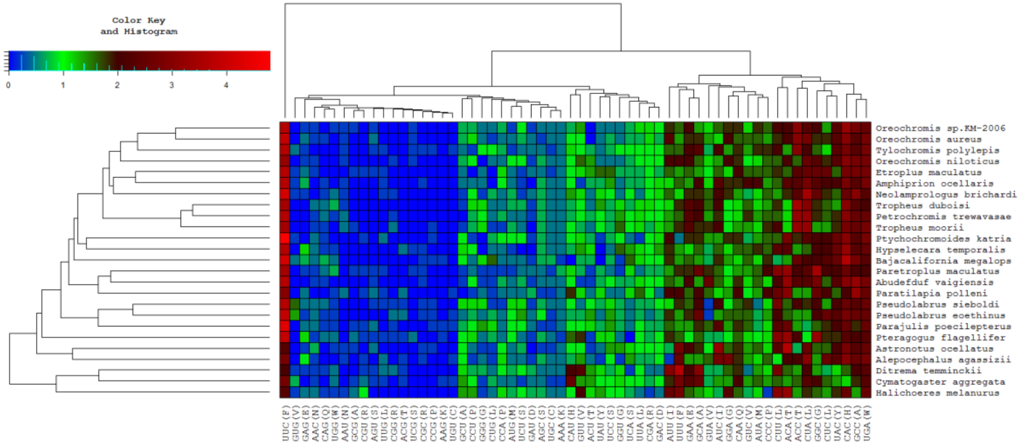

(b)

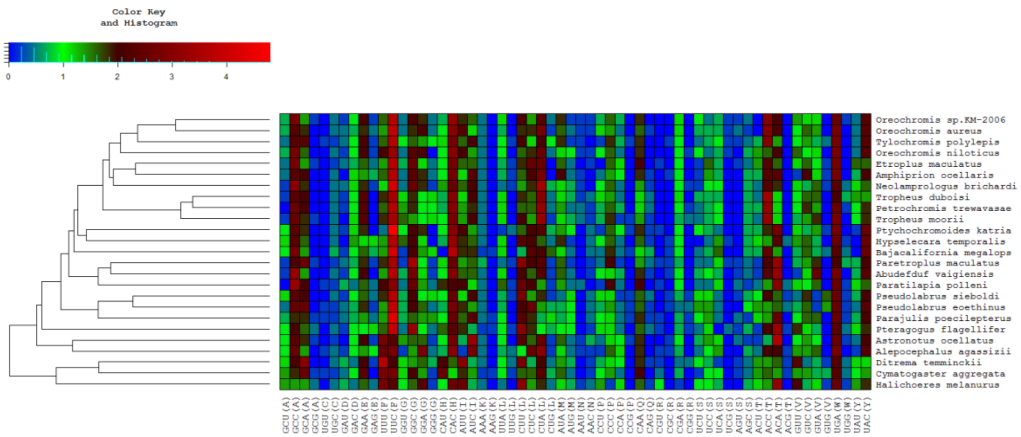

ATP synthase F0 subunit 8

(a)

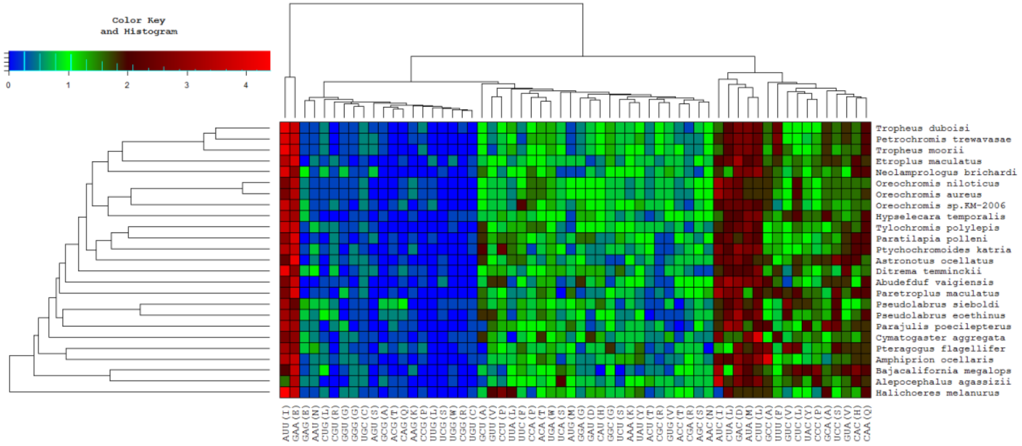

(b)

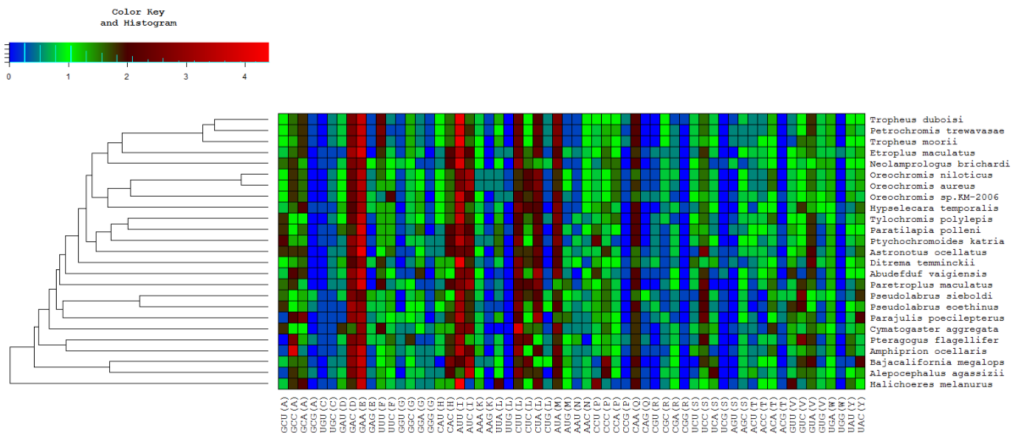

Figure S6 Codon usage. Shown is the codon distribution of all protein-coding genes for all considered species. Color key: The green region around one implies that a codon was observed about 1 time in 60 codons (matching the probability under uniform distribution; omitting the 4 stop codons), higher red and lower blue values indicate deviations of uniform distribution (as factors); thereby also amino acid distribution is visible. **(a)** Hierarchical clustering (Lance-Williams; average linkage method) facilitates the visual identification of deviations. **(b)** Synonymous codons are listed sequentially to allow for quick evaluation of relative synonymous codon usage.

(a)

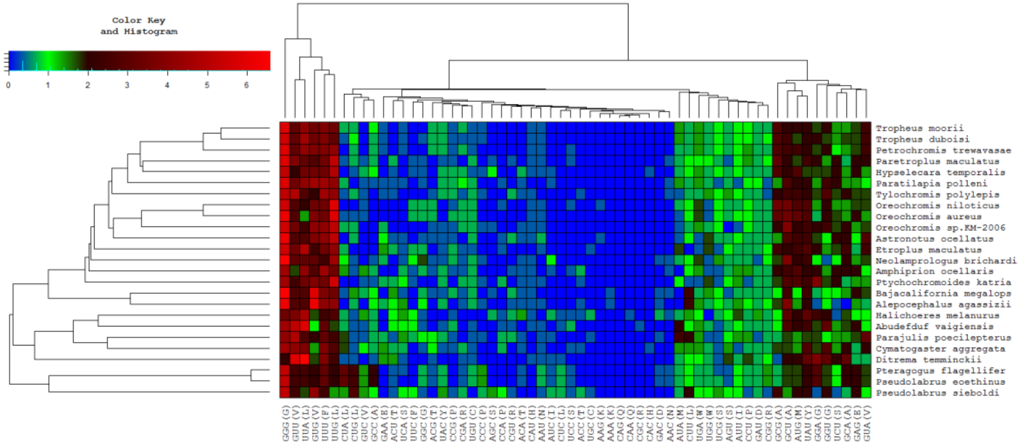

Cytochrome b

(b)

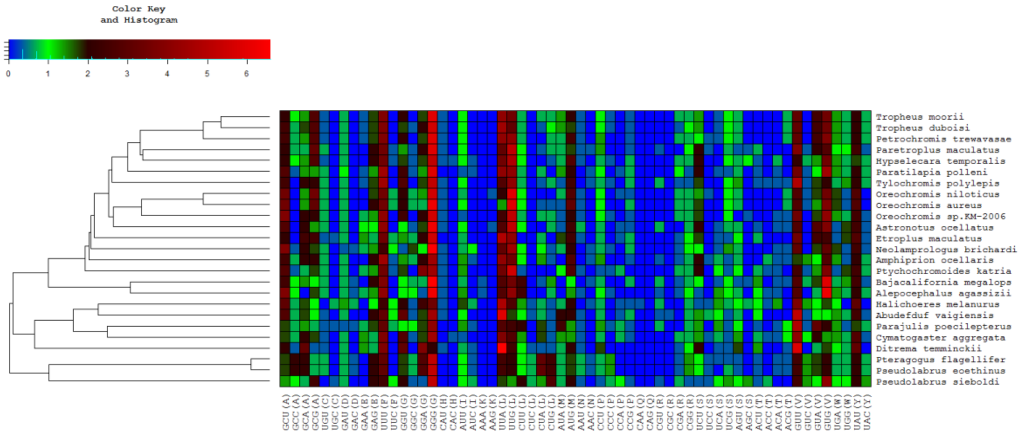

(a)

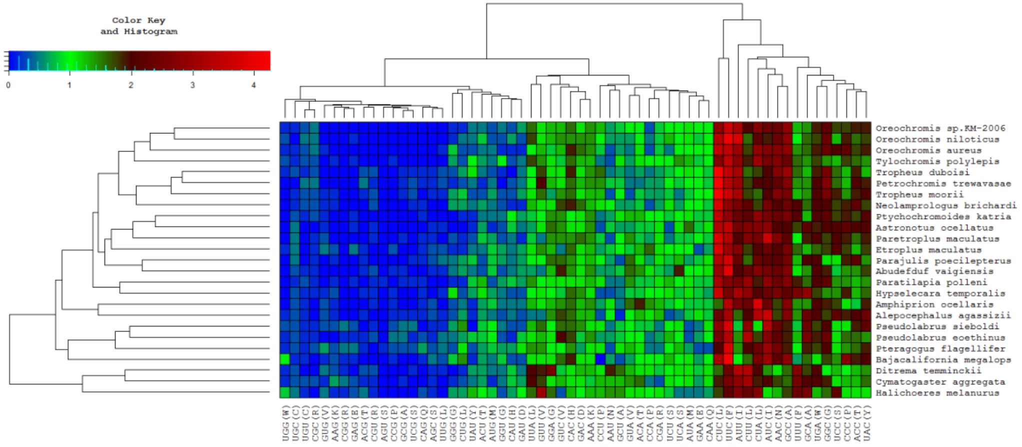

Cytochrome c oxidase subunit 1

(b)

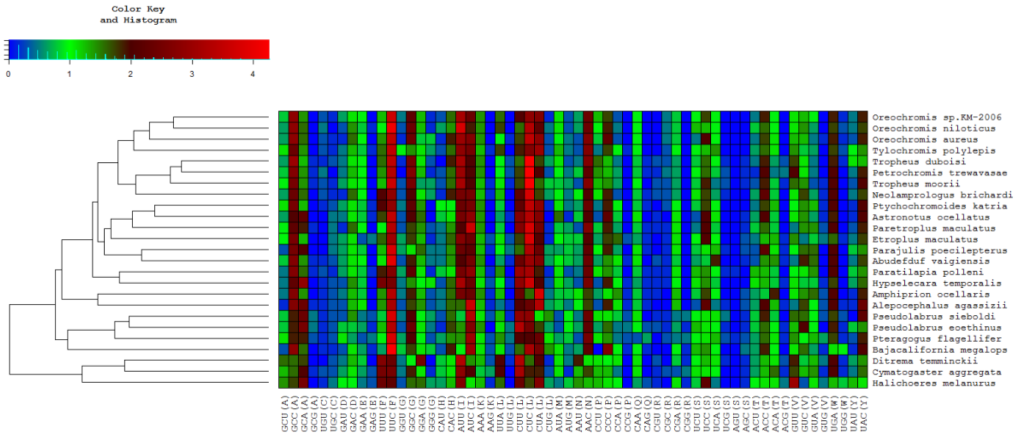

Figure S6 Codon usage. Shown is the codon distribution of all protein-coding genes for all considered species. Color key: The green region around one implies that a codon was observed about 1 time in 60 codons (matching the probability under uniform distribution; omitting the 4 stop codons), higher red and lower blue values indicate deviations of uniform distribution (as factors); thereby also amino acid distribution is visible. **(a)** Hierarchical clustering (Lance-Williams; average linkage method) facilitates the visual identification of deviations. **(b)** Synonymous codons are listed sequentially to allow for quick evaluation of relative synonymous codon usage.

(a)

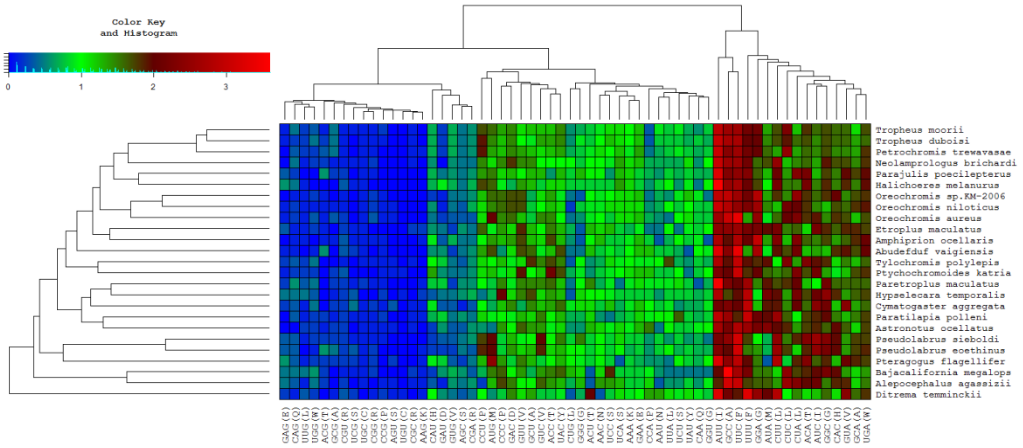

Cytochrome c oxidase subunit 2

(b)

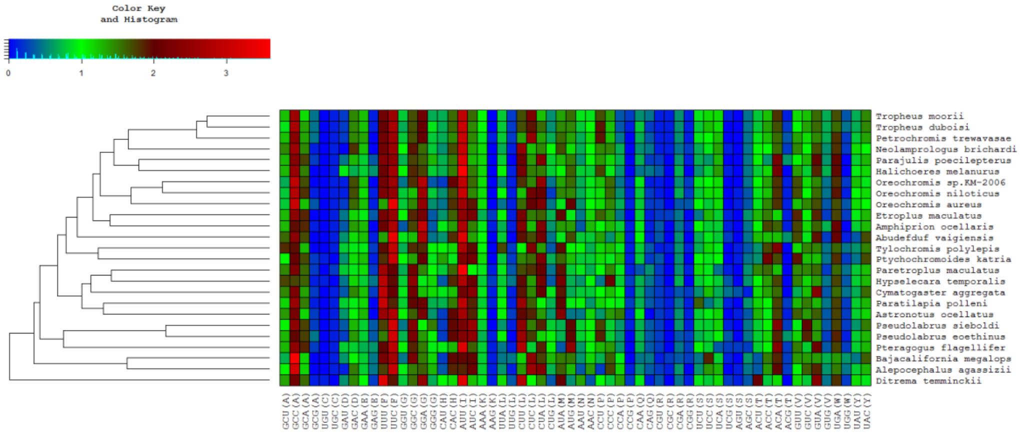

(a)

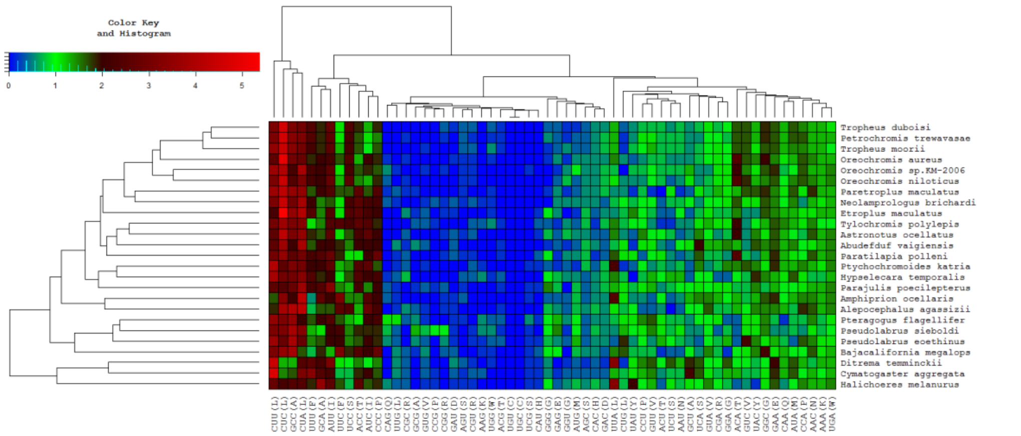

Cytochrome c oxidase subunit 3

(b)

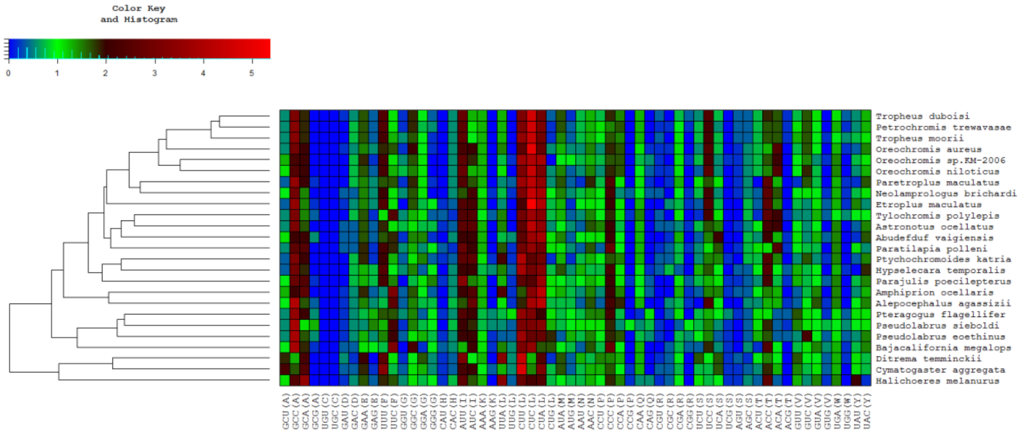

Figure S6 Codon usage. Shown is the codon distribution of all protein-coding genes for all considered species. Color key: The green region around one implies that a codon was observed about 1 time in 60 codons (matching the probability under uniform distribution; omitting the 4 stop codons), higher red and lower blue values indicate deviations of uniform distribution (as factors); thereby also amino acid distribution is visible. **(a)** Hierarchical clustering (Lance-Williams; average linkage method) facilitates the visual identification of deviations. **(b)** Synonymous codons are listed sequentially to allow for quick evaluation of relative synonymous codon usage.

(a)

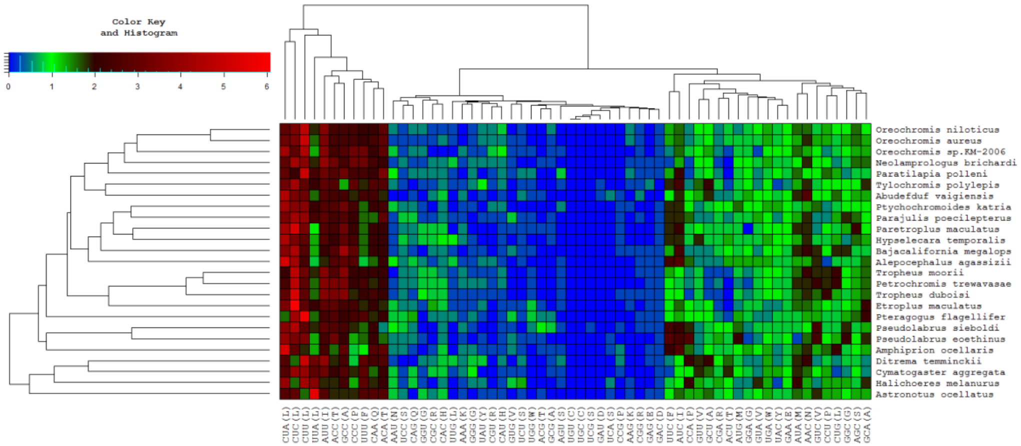

(a)

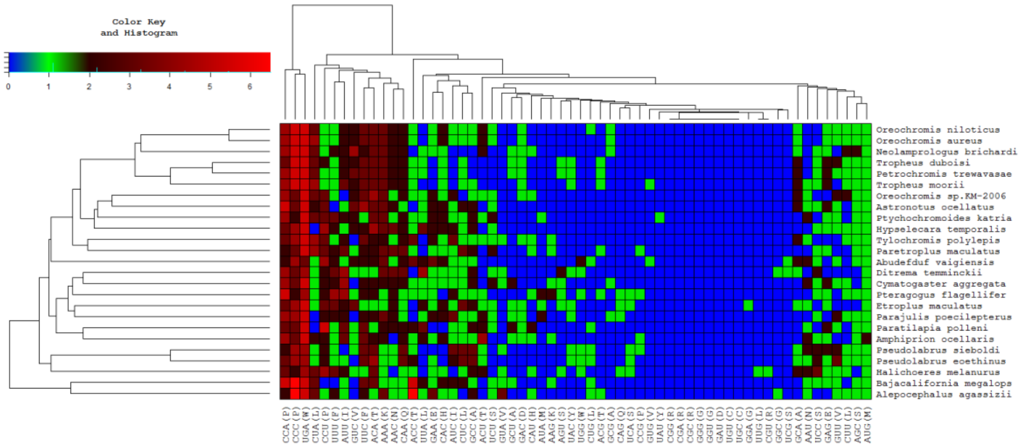

NADH dehydrogenase subunit 1

NADH dehydrogenase subunit 2

(b)

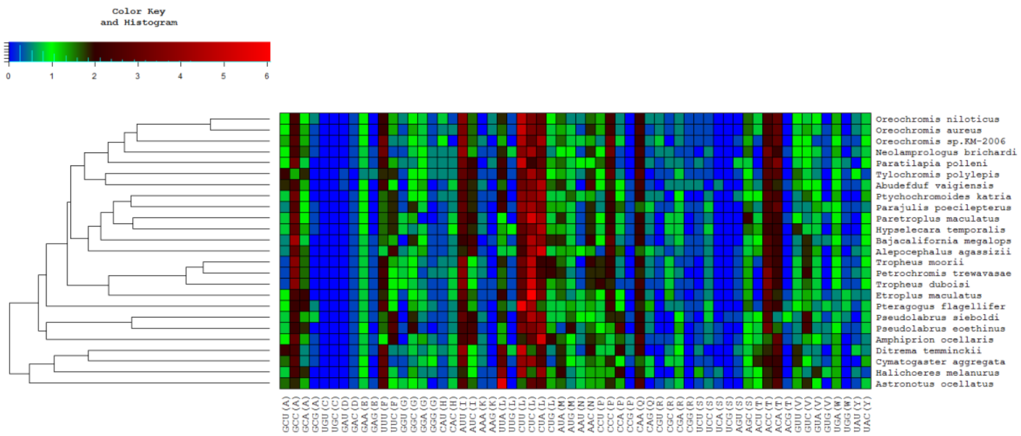

(b)

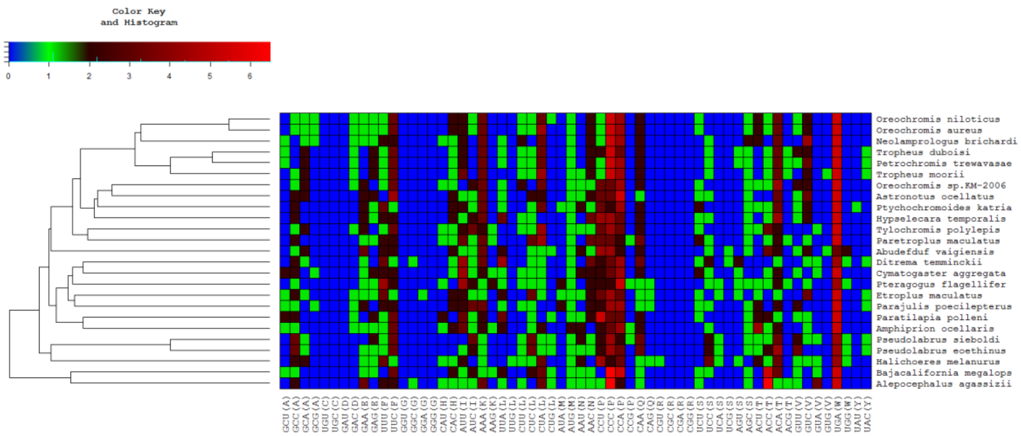

Figure S6 Codon usage. Shown is the codon distribution of all protein-coding genes for all considered species. Color key: The green region around one implies that a codon was observed about 1 time in 60 codons (matching the probability under uniform distribution; omitting the 4 stop codons), higher red and lower blue values indicate deviations of uniform distribution (as factors); thereby also amino acid distribution is visible. **(a)** Hierarchical clustering (Lance-Williams; average linkage method) facilitates the visual identification of deviations. **(b)** Synonymous codons are listed sequentially to allow for quick evaluation of relative synonymous codon usage.

NADH dehydrogenase subunit 3

(a)

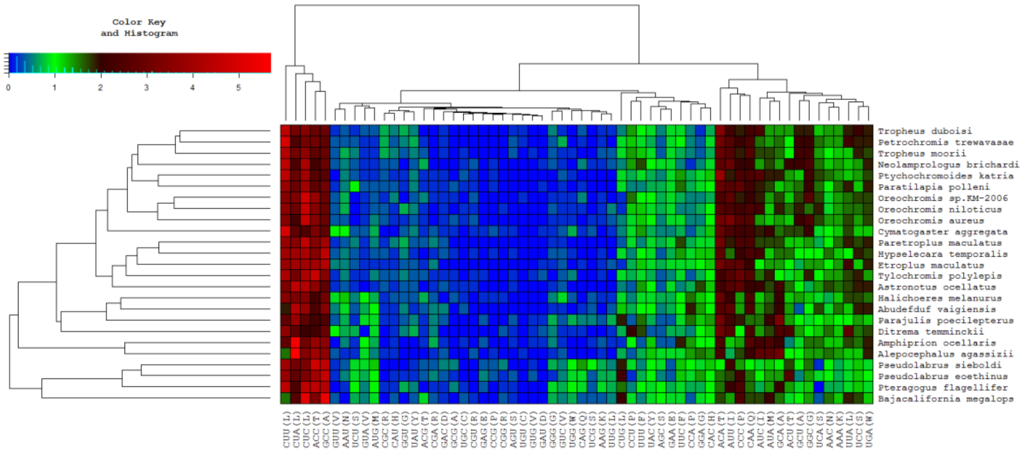

(b)

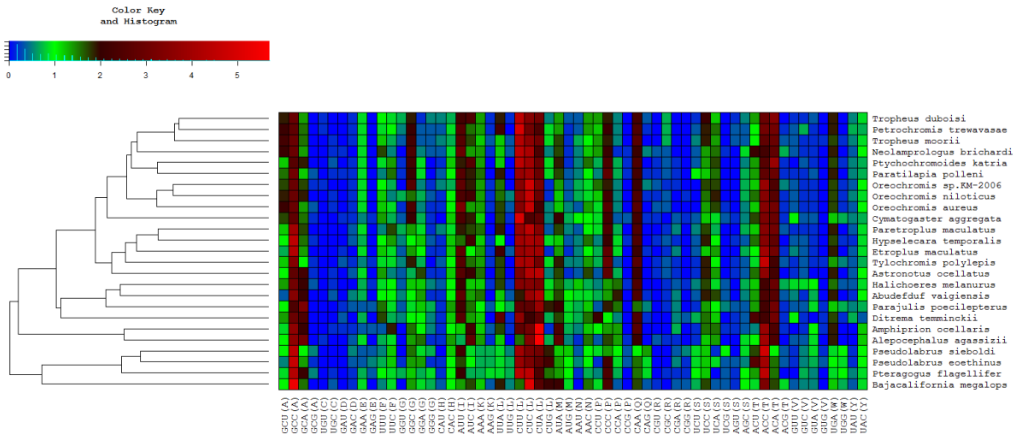

NADH dehydrogenase subunit 4

(a)

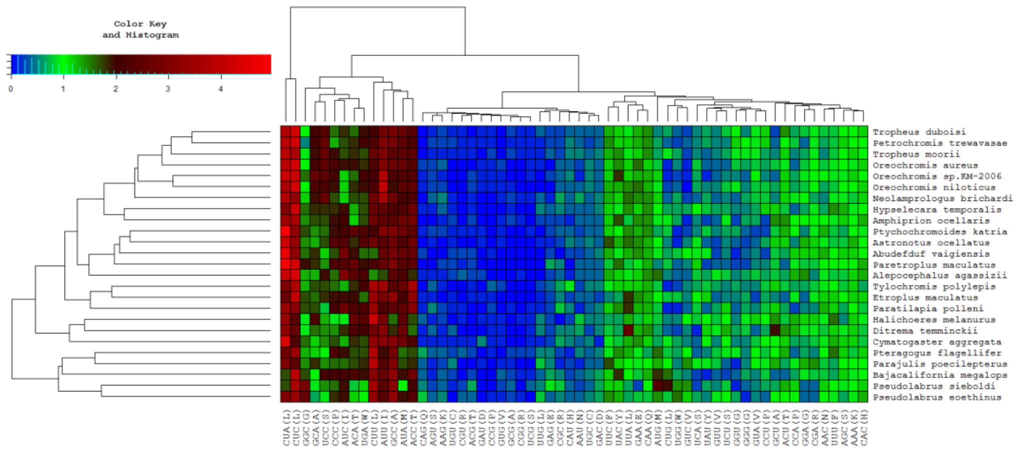

(b)

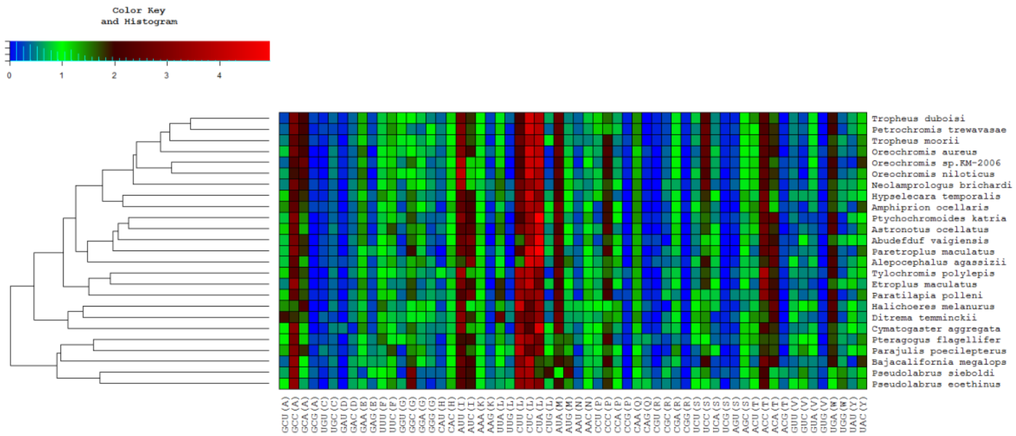

Figure S6 Codon usage. Shown is the codon distribution of all protein-coding genes for all considered species. Color key: The green region around one implies that a codon was observed about 1 time in 60 codons (matching the probability under uniform distribution; omitting the 4 stop codons), higher red and lower blue values indicate deviations of uniform distribution (as factors); thereby also amino acid distribution is visible. **(a)** Hierarchical clustering (Lance-Williams; average linkage method) facilitates the visual identification of deviations. **(b)** Synonymous codons are listed sequentially to allow for quick evaluation of relative synonymous codon usage.

(a)

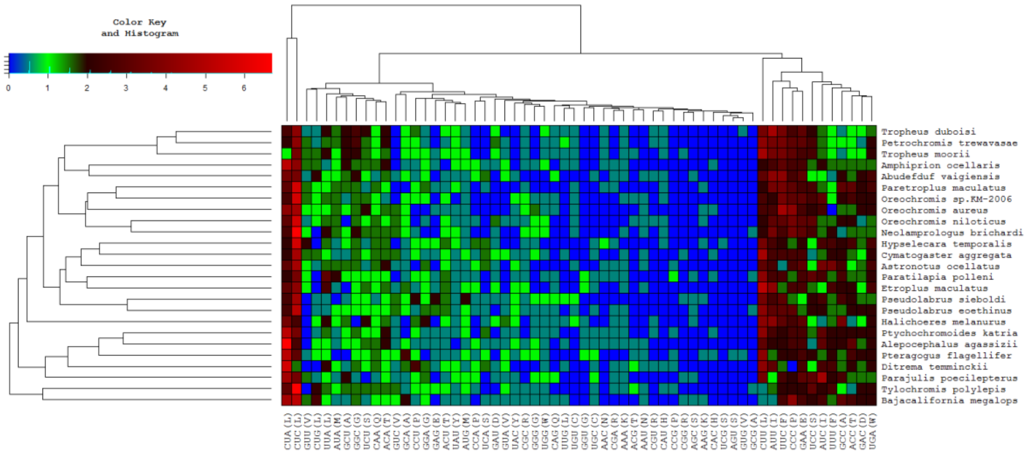

NADH dehydrogenase subunit 4L

(b)

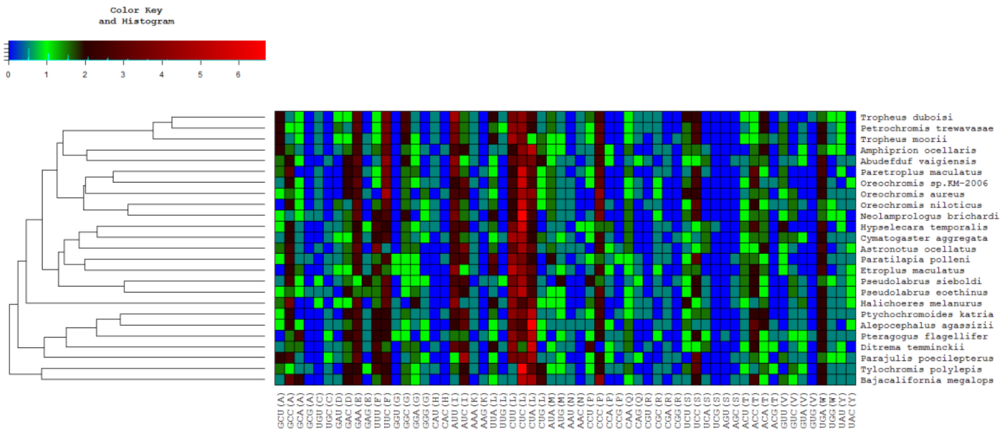

(a)

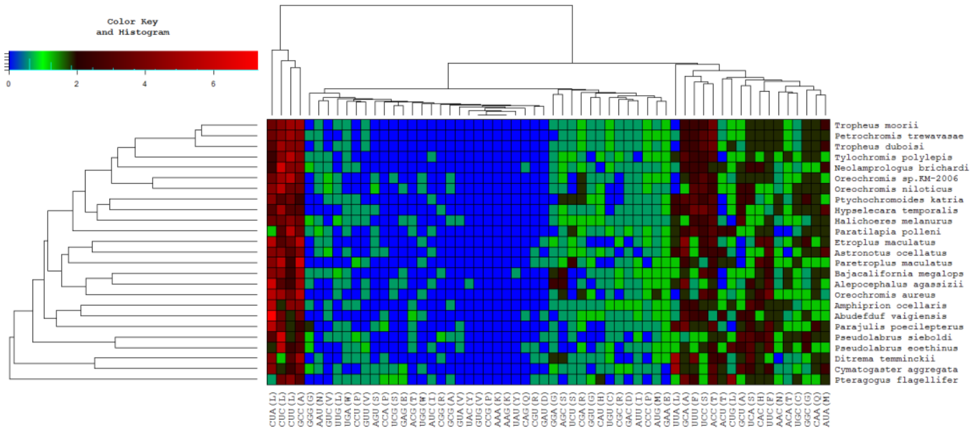

NADH dehydrogenase subunit 5

(b)

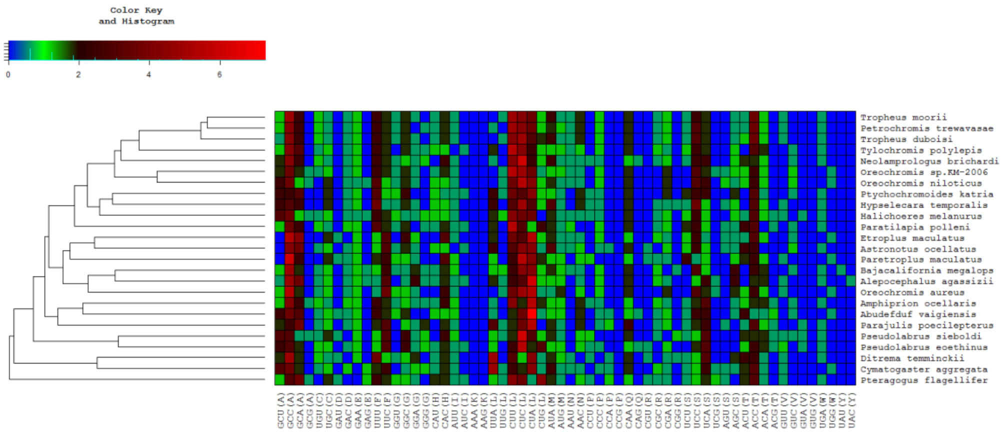

Figure S6 Codon usage. Shown is the codon distribution of all protein-coding genes for all considered species. Color key: The green region around one implies that a codon was observed about 1 time in 60 codons (matching the probability under uniform distribution; omitting the 4 stop codons), higher red and lower blue values indicate deviations of uniform distribution (as factors); thereby also amino acid distribution is visible. **(a)** Hierarchical clustering (Lance-Williams; average linkage method) facilitates the visual identification of deviations. **(b)** Synonymous codons are listed sequentially to allow for quick evaluation of relative synonymous codon usage.

(a)

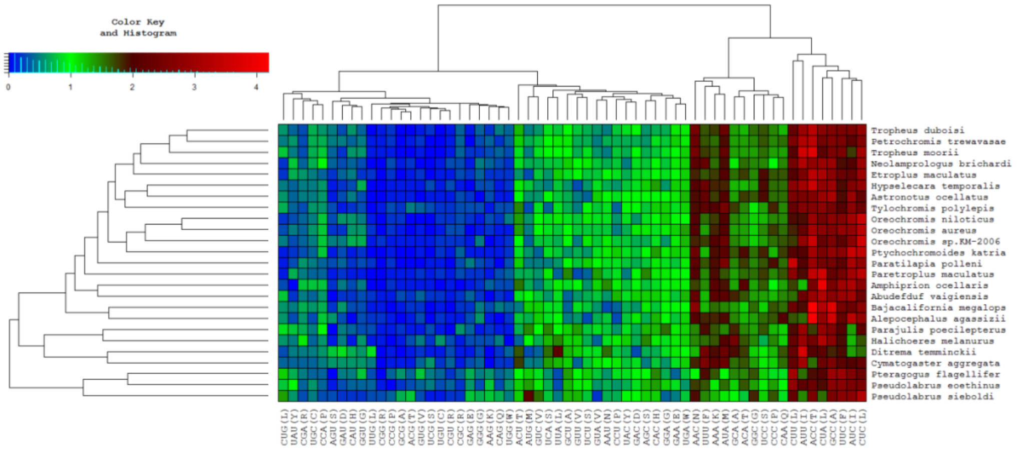

NADH dehydrogenase subunit 6

(b)

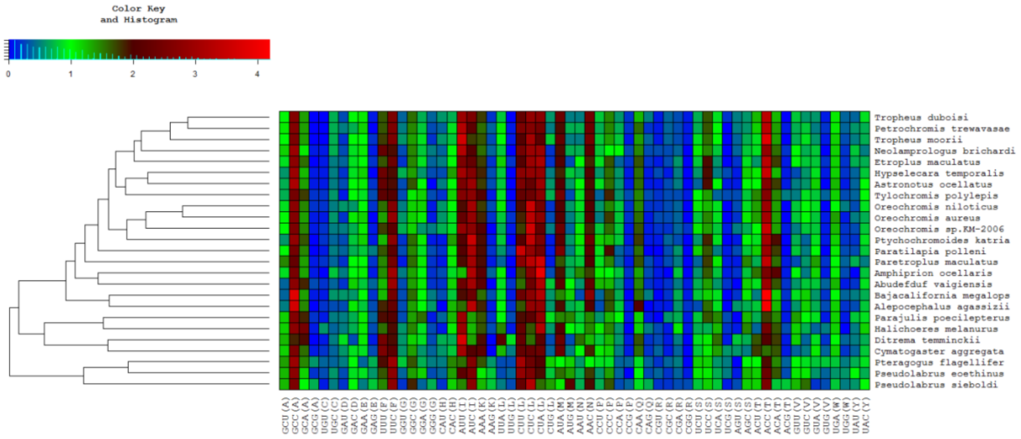

Figure S6 Codon usage. Shown is the codon distribution of all protein-coding genes for all considered species. Color key: The green region around one implies that a codon was observed about 1 time in 60 codons (matching the probability under uniform distribution; omitting the 4 stop codons), higher red and lower blue values indicate deviations of uniform distribution (as factors); thereby also amino acid distribution is visible. **(a)** Hierarchical clustering (Lance-Williams; average linkage method) facilitates the visual identification of deviations. **(b)** Synonymous codons are listed sequentially to allow for quick evaluation of relative synonymous codon usage.
